# Effectiveness and Adherence of Pharmacological vs. Non-Pharmacological Technology-Supported Smoking Cessation Interventions: An Umbrella Review

**DOI:** 10.3390/healthcare13080953

**Published:** 2025-04-21

**Authors:** Federica Di Spirito, Maria Pia Di Palo, Marina Garofano, Rosaria Del Sorbo, Gianluca Allegretti, Iman Rizki, Marianna Bartolomeo, Massimo Giordano, Massimo Amato, Alessia Bramanti

**Affiliations:** Department of Medicine, Surgery and Dentistry, University of Salerno, Via S. Allende, 84081 Baronissi, Italy; mgarofano@unisa.it (M.G.); rdelsorbo@unisa.it (R.D.S.); dott.allegrettigianluca@gmail.com (G.A.); i.rizki@studenti.unisa.it (I.R.); mbartolomeo@unisa.it (M.B.); masgiordano@unisa.it (M.G.); mamato@unisa.it (M.A.); abramanti@unisa.it (A.B.)

**Keywords:** smoking cessation, smoke, effectiveness, adherence, humans, health, technology, pharmaceutical preparations, medication adherence

## Abstract

**Background**: Smoking cessation has a crucial public health role. To overcome non-technological and technology-based smoking cessation intervention limitations, technology-supported programs were developed. **Objectives**: The present umbrella review aimed to evaluate the long-term effectiveness (≥6 months) of pharmacological vs. non-pharmacological technology-supported smoking cessation interventions on adult daily smokers and the related human health benefits. **Methods**: Following PRISMA guidelines, the protocol was registered on PROSPERO (CRD42024601824). Fifty systematic reviews were included, evaluated through AMSTAR-2, and qualitatively synthesized. **Results**: A total of 69,269 smokers underwent pharmacological (39,367) and non-pharmacological (29,902) technology-supported interventions. The biochemically-verified effectiveness assessed as continuous abstinence rates (CARs) and seven-day point prevalence abstinence (PPA) of pharmacological vs. non-pharmacological at 6 and 12 months were, respectively, CARs 9.06% vs. 14.85%, 7-day PPA 17.37% vs. 17.15%; CARs 8.51% vs. 9.08%, 7-day PPA 14.00% vs. 5.63%. The 6-month adherence rates were higher in the non-pharmacological group (41.37% vs. 83.43%). **Conclusions**: Non-pharmacological technology-supported interventions showed similar effectiveness and higher adherence at 6 months. At 12 months, the CARs were similar despite lower adherence. Adherence quality and consistency may be important for sustained success, probably due to the “reverse causality”. Non-pharmacological interventions showed similar effectiveness, lower costs, and shorter durations than pharmacological interventions.

## 1. Introduction

The World Health Organization (WHO) defines tobacco smoke as an epidemic that is one of the biggest problems for public health, killing 7 million people who directly smoke tobacco and 1.3 million people due to second-hand tobacco smoke every year around the world [1].

The WHO estimates that there are 1.3 billion tobacco smokers around the world and half of them die due to tobacco-related noncommunicable diseases [2], such as cardiovascular diseases [3], pneumological diseases [4], metabolic diseases [5], and oral diseases [6]. Furthermore, many studies report an association between smoking, impaired quality of life of smokers, and mental health issues, often in a dose-dependent form [7].

Therefore, smoking cessation strategies play a crucial role in public health, with both short- and long-term benefits reported for individuals who quit smoking [8]. A six-month period of abstinence is considered indicative of long-term smoking cessation, as it provides a reliable estimate of the proportion of individuals likely to maintain abstinence in subsequent years [8]. The long-term effectiveness of smoking cessation interventions has been estimated at approximately 50% of the continuous abstinence rate achieved at six months [8].

Multiple studies have demonstrated the effectiveness of non-pharmacological behavioral interventions in supporting smoking cessation, including in-person strategies such as brief behavioral advice from physicians [9] and the use of printed educational materials. Additional benefits have been observed with more intensive behavioral interventions, such as individual or group counselling sessions [10,11]. However, these intensive approaches typically involve greater costs and time commitments for healthcare providers, as well as longer waiting times for individuals seeking to quit smoking.

Pharmacological therapy for smoking cessation is employed to reduce the reinforcing effects of nicotine and alleviate withdrawal symptoms [12]. The most commonly used pharmacological treatments in cessation programs include nicotine replacement therapy administered via transdermal patches, gums, or tablets; bupropion, an antidepressant that enhances dopaminergic and noradrenergic activity; and varenicline, a partial nicotinic receptor agonist that stimulates dopamine release within the mesolimbic system [12].

In parallel with advances in digital health technologies and the growing implementation of telemedicine, telecommunications systems are increasingly being used to deliver medical counseling, diagnosis, treatment, and monitoring, particularly for individuals in remote or underserved areas [13]. Within this context, several digital interventions have been developed to support smoking cessation, reflecting the broader shift toward technology-enabled healthcare delivery.

The WHO clinical treatment guideline for tobacco cessation in adults [14] includes specific recommendations for behavioral support delivered through digital means. These digital interventions encompass a range of technologies, including telephone counseling, text messaging, mobile applications, artificial intelligence-based software, web-based resources, and email. While such approaches offer promising avenues for extending access to cessation support, particularly where in-person care is limited, current evidence on their effectiveness remains mixed [15]. Nevertheless, the WHO recommends that digital tobacco cessation interventions may be offered to adult smokers who are motivated to quit, either as standalone self-management tools or as adjuncts to other evidence-based smoking cessation strategies [14]. Such technology-based approaches offer clear advantages, including free access from home or public devices, availability on smartphones, and use at any time of day, every day. However, their uptake may be limited among older adults or individuals from lower-income backgrounds, who may have reduced access to or familiarity with digital technologies [16].

To address the limitations of both traditional in-person behavioral strategies and technology-based behavioral interventions, a variety of hybrid or technology-supported behavioral programs have been introduced. These programs combine non-technological and technology-supported behavioral programs in heterogeneous ways to enhance engagement and accessibility [17,18,19,20]. Despite evidence supporting the effectiveness of combining pharmacological treatments with in-person behavioral support to improve cessation outcomes [12], the long-term efficacy of integrating technological means alongside pharmacological or other non-pharmacological behavioral interventions remains insufficiently investigated [21]. In fact, the WHO [14] acknowledges that digital interventions may serve as valuable complements to other recommended cessation strategies, such as pharmacological therapy, but emphasizes the need for further research to identify the most effective combinations and delivery formats.

Moreover, evaluating the impact of smoking cessation interventions requires careful consideration of treatment adherence, which plays a critical role in determining overall effectiveness. Importantly, the relationship between adherence and efficacy may be bidirectional—a phenomenon referred to as “reverse causality” [19,22]. For example, poor adherence can diminish treatment success, and early relapse or perceived failure may reduce motivation to adhere to the intervention further, leading to lower treatment engagement over time. Failure to account for this dynamic can result in an overestimation of the causal effect of adherence on treatment efficacy [19].

In light of these considerations, the primary aim of the present umbrella review was to evaluate the long-term effectiveness of and adherence (at least 6 months) to different pharmacological and non-pharmacological technology-supported smoking cessation interventions. The secondary aim was to assess the related benefits for human health, providing deeper insights into cardiovascular, pulmonary, metabolic, psychological, and oral health.

## 2. Materials and Methods

### 2.1. Study Protocol

The protocol of the present umbrella review was registered in the International Prospective Register of Systematic Reviews PROSPERO (registration number CRD42024601824), and it was developed before beginning the literature searches, data extraction, collection, and synthesis, according to the Preferred Reporting Items for Systematic Reviews and Meta-Analyses (PRISMA) statement [23].

The research questions, the search strategy, and the eligibility criteria were established in compliance with the PICO model [24]. The research questions were: “What is the long-term effectiveness (at least 6 months) of technology-supported pharmacological vs. non-pharmacological smoking cessation interventions? And what are the related benefits for human health?”, focusing on the following:

- Population (P): adult subjects (≥18 years of age) who are current daily smokers of heated tobacco products as defined by the WHO [25];
- Intervention (I): pharmacological technology-supported smoking cessation interventions (any) from all types of providers and in all settings;
- Comparison (C): non-pharmacological technology-supported smoking cessation interventions (any) from all types of providers and in all settings;
- Outcome(s) (O):
  - Primary outcome(s): continuous abstinence rates (CARs) and/or point prevalence abstinence (PPA) rates at least 6 months after the start of the intervention, measured by self-reported and/or biochemically verified (e.g.,: carbon monoxide or cotinine test) as recommended by the Society for Research on Nicotine and Tobacco and the Russell Standard [26,27];
  If reported, we also extracted the secondary outcome(s):
  - Secondary outcome(s): adherence to, satisfaction with, and acceptability of smoking cessation interventions; medical (cardiovascular, pneumological, metabolic, and psychological) and oral (periodontal and peri-implant, mucosal lesions) parameters before and after smoking cessation interventions.

### 2.2. Search Strategy

The electronic and manual search was carried out without date restrictions through 10 October 2024 to retrieve systematic reviews with or without meta-analysis in the English language concerning pharmacological or non-pharmacological technology-supported smoking cessation interventions (any) reporting primary smoking cessation outcomes at least 6 months after the start of the intervention.

The electronic search was conducted across the MEDLINE/PubMed, Scopus, Web of Science (Core Collection) databases, and the PROSPERO register, by three independent reviewers (F.D.S., M.P.D.P., and A.B.), combining keywords with Boolean operators, as follows: (“smoking cessation” OR “quitting smoking” OR “giving up smoking” OR “stopping smoking” OR “ex-smokers”) AND (“cell-phone” OR smartphone OR “computers” OR “computer handheld” OR “online systems” OR “technology” OR “social media” OR “mobile applications” OR “text messaging” OR “virtual reality” OR “augmented reality” OR telemedicine OR “internet-based intervention” OR multimedia OR “electronic mail”) AND (“systematic review”).

The only filters electronically applied according to the options for each database were: “Systematic review” and “English” on MEDLINE/PubMed; “Review” and “English” on Scopus and Web of Science; and “English” on PROSPERO.

### 2.3. Study Selection and Eligibility Criteria

The records of the electronic search were collected after the definition of the eligibility criteria by three independent reviewers (F.D.S., M.P.D.P., and A.B.) and were screened. Any issue of disagreement was discussed by involving a fourth author (M.A.). The records collected in duplicate were eliminated. The remaining titles and abstracts were screened, and the records not in accordance with our purpose were considered not eligible and eliminated. The full texts of the remaining unclear records were obtained to evaluate their compliance with the eligibility criteria and those not compliant with the inclusion criteria were eliminated.

An additional manual search was carried out as already described for the electronic search by consulting the reference lists of the included systematic reviews to find additional eligible records.

If any full text of the unclear records was not available, the authors were contacted to obtain the full text.

All references of the included systematic reviews were collected using the 2.80.1 version of the Mendeley Reference Manager tool.

The inclusion criteria were systematic reviews with or without meta-analysis in humans and published in the English language without date restrictions that evaluated the effectiveness in adult subjects (≥18 years of age) who were current daily smokers of heated tobacco, were not drinkers of alcohol or who had other substance abuse disorders, of pharmacological or non-pharmacological technology-supported smoking cessation interventions (any) and reporting primary smoking cessation outcomes at least 6 months after the start of the intervention. No restrictions concerning the number or the design of the studies included in each systematic review, the sample size, subjects’ comorbidities, the number of smoked cigarettes or the type of smoked heated tobacco, or the pharmacological or non-pharmacological technology-supported smoking cessation interventions were applied.

The exclusion criteria were not systematic reviews with or without meta-analysis; earlier versions of updated systematic reviews were excluded (only the most recent version was included); not English language; pediatric subjects (<18 years of age), pregnant or lactating women, subjects drinking alcohol or who had other substance abuse disorders; not current daily smokers; not heated tobacco; smoking cessation interventions not supported by technology; smoking cessation interventions based only on technology tools; and pharmacological or non-pharmacological technology-supported smoking cessation interventions (any) reporting primary smoking cessation outcomes for less than 6 months after the start of the intervention. Pharmacological or non-pharmacological technology-supported smoking cessation interventions were excluded if the technology-based programs were used only to assess the primary smoking cessation outcome at the follow-ups.

### 2.4. Data Extraction and Collection

Three independent reviewers (F.D.S., M.P.D.P., and A.B.) extracted and collected the data from the included systematic reviews using the standardized data extraction form compliant with the model proposed for intervention reviews of non-randomized clinical trials (RCTs) and RCTs [28]. Any issue of disagreement was discussed by involving a third author (M.A.).

The following data were extracted and collected from each included systematic review with or without meta-analysis:

- Study characteristics: first author, year of publication, journal, number and design of the studies included, meta-analysis (yes/no), quality assessment, funding information;
- Population characteristics: sample size, mean and range age, gender ratio, comorbidities, mean number of smoked cigarettes/days, severity of nicotine addiction, motivation to quit smoking;
- Intervention: type of smoking cessation intervention (pharmacological or non-pharmacological technology-supported intervention), type of technology-supported interventions, type of pharmacological intervention (if any), type of non-pharmacological intervention (if any), intervention duration;
- Outcome(s):
  - Primary outcome(s): CARs, PPA, number of smoked cigarettes/day, reasons for failure (if any);
  - Secondary outcome(s): adherence to, satisfaction with, and acceptability of smoking cessation interventions; medical (cardiovascular, pneumological, metabolic, and psychological) and oral (periodontal and peri-implant, mucosal lesions) parameters before and after smoking cessation interventions.

If smoking cessation outcomes were available for both complete cases and intention-to-treat (ITT) analysis, only ITT rates were extracted to analyze all participants included in the original groups and whether or not they completed the intervention and the follow-up assessments. The ITT analysis has been recommended for studies that intend to evaluate the effectiveness of an intervention [29]. In accordance with the ITT analysis, participant dropouts during the smoking cessation interventions or those lost to follow-up assessments were considered smokers.

Only the data concerning pharmacological or non-pharmacological technology-supported smoking cessation interventions provided for at least 6 months for adult subjects (≥18 years of age) who were current daily smokers were extracted and collected.

### 2.5. Data Synthesis

The extracted and collected data from the included systematic reviews were qualitatively synthesized using Microsoft Excel software 2019 (Microsoft Corporation, Redmond, Washington, DC, USA) through a descriptive statistical analysis:

- To evaluate the long-term effectiveness (at least 6 months) of pharmacological or non-pharmacological technology-supported smoking cessation interventions;
- To compare the long-term effectiveness (at least 6 months) of pharmacological vs. non-pharmacological technology-supported smoking cessation interventions;
- To evaluate the adherence to, satisfaction with, and acceptability of pharmacological or non-pharmacological technology-supported smoking cessation interventions;
- To compare the adherence to, satisfaction with, and acceptability of pharmacological vs. non-pharmacological technology-supported smoking cessation interventions;
- To evaluate the long-term effectiveness (at least 6 months) of smoking cessation on medical (cardiovascular, pneumological, metabolic, and psychological) and oral (periodontal and peri-implant, mucosal lesions) parameters before and after smoking cessation interventions of pharmacological or. non-pharmacological technology-supported smoking cessation interventions.
- To compare the long-term effectiveness (at least 6 months) of smoking cessation on medical (cardiovascular, pneumological, metabolic, and psychological) and oral (periodontal and peri-implant, mucosal lesions) parameters before and after smoking cessation interventions pharmacological vs. non-pharmacological technology-supported smoking cessation intervention.

### 2.6. Quality Assessment

The systematic reviews with or without meta-analysis included in the present study were qualitatively assessed by three independent reviewers (F.D.S., M.P.D.P., and A.B.) on 18 November 2024, using the critical appraisal tool for systematic reviews of RCTs and non-RCTs: the Assessing the Methodological quality of Systematic Reviews 2 (AMSTAR-2) [30]. Any issue of disagreement was discussed by involving a fourth author (M.A.).

## 3. Results

### 3.1. Study Selection

The electronic search via databases and register generated a total of 931 records, in particular, 195 from PubMed/MEDLINE, 268 from Scopus, 146 from Web of Science, and 322 from the PROSPERO register. The screening of the record’s title allowed the identification of 234 duplicate records that were removed. The screening of the remaining 697 record’s titles and abstracts allowed the identification of 411 not relevant records that were removed.

The screening of the remaining 286 records was conducted by reading the full texts. The full text of one record was not available; the authors were contacted to obtain the full text but due to a lack of response the record was excluded. Another 237 records were excluded according to the inclusion and exclusion criteria for the following reasons: not smoking cessation outcome data or inability to extract smoking cessation outcome data ≥ 6 months (*n* = 69); systematic review ongoing (*n* = 65); not technological smoking cessation intervention or inability to extract data from a technological-supported intervention (*n* = 60); range age not specified, or age population < 18 years old, or inability to extract data from ≥18 years old (*n* = 29); not a systematic review or was a systematic review subsequently updated (*n* = 18); inability to extract data from the technological-supported intervention performed in the ≥18 years old group (*n* = 1); not systematic review in the English language (*n* = 1); pregnant women (*n* = 1); not smokers of heated tobacco (*n* = 1).

The study selection process of the records obtained from the electronic search via databases identified a total of 40 systematic reviews [31,32,33,34,35,36,37,38,39,40,41,42,43,44,45,46,47,48,49,50,51,52,53,54,55,56,57,58,59,60,61,62,63,64,65,66,67,68,69,70] that were included in the present study.

The same methodology for the study selection process was applied to the records identified by a manual search of the reference lists of the 40 systematic reviews [31,32,33,34,35,36,37,38,39,40,41,42,43,44,45,46,47,48,49,50,51,52,53,54,55,56,57,58,59,60,61,62,63,64,65,66,67,68,69,70] included via the electronic search.

The manual search via the reference lists generated a total of 2318 records. The screening of their titles allowed the identification of 364 duplicate records that were removed. The screening of the remaining 1954 record’s titles and abstracts allowed the identification of 1879 not relevant records that were removed.

The screening of the remaining 75 records was conducted by reading the full texts that were retrieved for all records; therefore, no contact with the authors was necessary. A total of 65 records were excluded according to the inclusion and exclusion criteria for the following reasons: not smoking cessation outcome data ≥ 6 months (*n* = 20); not a systematic review or was a systematic review subsequently updated (*n* = 15); range age not specified or inability to extract data from ≥18 years old (*n* = 14); not technological smoking cessation intervention or inability to extract data from technological-supported intervention (*n* = 14); inability to extract data from the technological-supported intervention performed in the ≥18 years old group (*n* = 2).

The study selection process of the records obtained from the manual search via databases identified a total of 10 systematic reviews [71,72,73,74,75,76,77,78,79,80] that were included in the present study.

**Figure 1:**
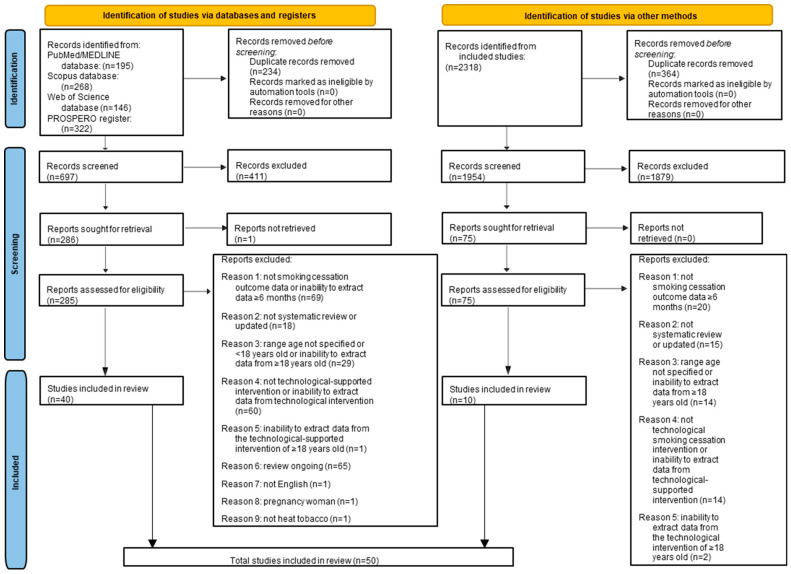
The study selection process of records retrieved through electronic and manual searches illustrated in a PRISMA 2020 flowchart.

Finally, 50 systematic reviews [31,32,33,34,35,36,37,38,39,40,41,42,43,44,45,46,47,48,49,50,51,52,53,54,55,56,57,58,59,60,61,62,63,64,65,66,67,68,69,70,71,72,73,74,75,76,77,78,79,80] were included in the present study, as shown in Figure 1.

### 3.2. Study Characteristics and Qualitative Synthesis

Data from the 50 included systematic reviews [31,32,33,34,35,36,37,38,39,40,41,42,43,44,45,46,47,48,49,50,51,52,53,54,55,56,57,58,59,60,61,62,63,64,65,66,67,68,69,70,71,72,73,74,75,76,77,78,79,80] on the long-term effectiveness of pharmacological or non-pharmacological technology-supported smoking cessation intervention in adult daily smokers (≥18 years old) were extracted into two tables: Table S1 in Supplementary File S1, which reports the study and population characteristics, smoking cessation intervention and related effectiveness, adherence, satisfaction, acceptability and changes in human health; and Table S2 in Supplementary File S2, which reports the smoking cessation intervention characteristics.

The design of the 165 studies included in the 50 systematic reviews [31,32,33,34,35,36,37,38,39,40,41,42,43,44,45,46,47,48,49,50,51,52,53,54,55,56,57,58,59,60,61,62,63,64,65,66,67,68,69,70,71,72,73,74,75,76,77,78,79,80] (22 systematic reviews without meta-analysis [32,35,37,38,39,40,42,45,47,48,49,52,54,56,57,58,59,63,66,70,73,80], and 28 with meta-analysis [31,33,34,36,41,43,44,46,50,51,53,55,60,61,62,64,65,67,68,69,71,72,74,75,76,77,78,79]) were 154 randomized controlled trials, 7 pilot studies, 3 cohort studies, and 1 observational study.

A total of 69,269 adult daily smokers underwent a pharmacological or non-pharmacological technology-supported smoking cessation intervention and were included in the present umbrella review [31,32,33,34,35,36,37,38,39,40,41,42,43,44,45,46,47,48,49,50,51,52,53,54,55,56,57,58,59,60,61,62,63,64,65,66,67,68,69,70,71,72,73,74,75,76,77,78,79,80].

#### 3.2.1. Population Characteristics

The mean age was reported for 21,509 smokers and was 37.69 years old [32,33,34,39,40,41,43,44,45,47,48,50,51,53,54,55,56,59,61,63,64,66,67,68,69,70,71,72,73,74]; the gender ratio was 1 male to 1.33 females and was reported for 34,272 subjects (14,700 males and 19,572 females) [32,33,34,35,36,37,39,40,41,43,44,45,46,47,48,49,50,51,53,55,61,63,64,66,67,68,69,70,71,72,74,75,79].

Of the 69,269 smokers, 11.88% (*n* = 8227) were hospitalized patients for not defined diseases [42,46,53,64]; 2.02% (*n* = 1396) were affected by Human Immunodeficiency Virus (HIV) [34,36,37,43,50,52,53,64,67,68,71,79]; 1.83% (*n* = 1269) were affected by psychiatric disorders [34,47,59]; 1.43% (*n* = 989) were hospitalized patients pre not-defined surgery [31,46,57,65]; 1.00% (*n* = 690) were cancer survivors [53,64,71]; 0.61% (*n* = 424) were hospitalized patients for cardiac diseases [31,42]; 0.27% (*n* = 184) were hospitalized cancer patients for pre resection surgery [57]; 0.23% (*n* = 162) were hospitalized for psychiatric disorders [34,47]; 0.18% (*n* = 125) were hospitalized for pre-cardiac surgery [46]; 0.14% (*n* = 96) were hospitalized for pre-orthopedic or general surgery [46,65]; and 0.06% (*n* = 42) were affected by chronic obstructive pulmonary diseases [64].

#### 3.2.2. Characteristics of Smoking Behaviors

The mean number of cigarettes smoked per day was registered before the smoking cessation intervention for 6636 smokers and was 16.60 cigarettes per day [35,41,45,47,50,51,53,61,63,64,66,67,68,70,71,72,74,75], while after the intervention it was 7.69 for 1505 smokers [47,80].

To assess the nicotine severity dependence, the Fargström Test for Nicotine Dependence (FTND) and the Heaviness Smoking Index (HIS) were used. The mean score of the FTND was 4.78 before the intervention for 3260 smokers [40,41,47,50,51,53,59,61,62,63,64,67,68,71,72,74,76], and 0.81 for 205 smokers after the intervention [34,47,50,53,64,71]; the HIS was used for 242 smokers and the mean score was 2.71 before the intervention [33], while no data was available after the intervention.

#### 3.2.3. Health Status

No data on cardiovascular parameters were available before and after the smoking cessation interventions. However, cardiovascular complications occurred in 2% of 96 patients who stopped smoking before orthopedic or general surgery [46,65].

As pneumological parameters, the mean ratio between the forced expiratory volume in one second (FEV1) and the forced vital capacity (FVC) registered at the end of the smoking intervention of 42 patients affected by chronic obstructive pulmonary disease amounted to FEV1/FVC < 70% [64]. Furthermore, no pneumological complications occurred in 96 patients who stopped smoking before orthopedic or general surgery [46,65].

As psychological parameters, the Beck Depression Inventory and the Brief Psychiatric Rating Scale were administered to 226 smokers and were significantly improved from baseline to 36 months, while the Short Form Survey on General Functioning and the Global Assessment of Functioning were not significantly improved at any time points [62,76].

No data on metabolic or oral parameters were evaluated before or after the smoking cessation interventions.

#### 3.2.4. Technology-Supported Smoking Cessation Intervention Characteristics and Outcomes

The smoking cessation interventions’ duration was 1 month for 268 (0.39%) smokers [34,53,64,71], 4–6 weeks for 418 (0.60%) [66,75], 6 weeks for 652 (0.94%) [32,39,53], 7 weeks for 866 (1.25%) [31], 2 months for 699 (1.01%) [31,36,37,46,50,54,65,73,79], 3 months for 2472 (3.57%) [42,49,52,53,56,64,68,69,70,71,72,74,80], 4 months for 3094 (4.47%) [47,66,67,80], 6 months for 3615 (5.22%) [31,32,33,42,43,44,47,53,64,71,80], and 12 months for 4622 (6.67%) [34,40,42,46,50,53,64,70,71,74,75]. For the other participants, the smoking cessation intervention duration was not specified.

The smoking cessation interventions’ effectiveness was evaluated as CARs or PPA, both self-reported and biochemically verified by cotinine or carbon monoxide (CO) tests at different time points.

The effectiveness assessed as CARs self-reported was: CARs at 6 months was 1413 (8.84%) for 15,990 smokers [41,42,49,53,58,64,67,71,72,75]; CARs at 12 months was 77 (11.79%) for 653 smokers [45,53].

The effectiveness assessed as CARs biochemically verified by cotinine or CO tests was: 4 months CARs at 6 months was 1446 (53.16%) for 2720 smokers [31,32,33,37,43,44]; 5 months CARs at 6 months was 22 (23.40%) for 94 smokers [31]; 5.5 months CARs at 6 months was 24 (19.05%) for 126 smokers [36,37,79]; 6 months CARs at 6 months was 279 (9.26%) for 3014 smokers [31,33,43,63,66,67]; 6 months CARs at 12 months was 20 (15.15%) for 132 smokers [64]; 10 months CARs at 12 months was 1104 (47.12%) for 2343 smokers [32,33,43,44]; and 12 months CARs at 12 months was 288 (8.64%) for 3334 former smokers [31,34,37,41,56,63,77,79].

The effectiveness assessed as PPA self-reported was: PPA at 6 months (time not defined) was 40 (17.54%) for 228 smokers [49,67]; 2 days PPA at 6 months was 20 (9.80%) for 204 [66]; 7 days PPA at 6 months was 4056 (17.49%) for 23,185 smokers [31,38,41,42,44,50,51,53,54,59,61,64,66,67,68,70,71,72,73,75,80]; 30 days PPA at 6 months was 3607 (22.20%) for 1650 smokers [34,42,45,53,59,60,64,71]; PPA at 12 months (time not defined) was 128 (13.87%) for 923 smokers [46,48,49,65,67,69,77]; 7 days PPA at 12 months was 1953 (22.87%) for 8541 smokers [41,42,44,50,51,53,61,62,64,70,71,72,74,75,76,78,80]; 30 days PPA at 12 months was 171 (8.44%) for 2025 smokers [42,78]; 30 days PPA at 15 months was 66 (9.57%) for 690 smokers [53,64,71]; 7 days PPA at 18 months was 18 (7.96%) for 226 smokers [62,76]; 7 days PPA at 24 months was 303 (19.71%) for 1537 smokers [62,75,76]; 7 days PPA at 30 months was 18 (7.96%) for 226 smokers [62,76]; 7 days PPA at 36 months was 18 (7.96%) for 226 smokers [62,76].

The effectiveness assessed as PPA biochemically verified by cotinine or CO tests was: 2 days PPA at 6 months was 55 (13.75%) for 400 smokers [40,50,53,64,74]; 7 days PPA at 6 months was 1224 (17.34%) for 7060 smokers [31,32,34,35,36,37,38,42,45,47,50,53,55,56,57,59,63,64,66,67,68,69,71,72,74,79,80]; 30 days PPA at 6 months was 649 (15.49%) for 4190 smokers [31,42,44,50,53,55,64,66]; 7 days PPA at 7 months was 24 (24.00%) for 100 smokers [32,39]; 7 days PPA at 9 months was 60 (20.69%) for 290 smokers [34,50,53,64,71]; PPA at 12 months (time not defined) was 52 (15.66%) for 332 smokers [46,48,65,69]; 7 days PPA at 12 months was 664 (10.06%) for 6602 smokers [34,37,41,42,47,50,53,63,64,67,71,75,77,79,80]; 30 days PPA at 12 months was 69 (11.50%) for 300 smokers [42]; 7 days PPA at 18 months was 71 (18.25%) for 389 smokers [34,47].

Other effectiveness rates assessed as PPA but in which the methods of verification (self-reported/cotinine or CO test) were not reported were: PPA at 7 months was 21 (28.38%) for 74 smokers [70]; PPA at 12 months was 213 (23.10%) for 922 smokers [46,70,77]; 7 days PPA at 12 months was 102 (34.46%) for 296 smokers [46].

The adherence rate assessed as the number of subjects who completed the smoking cessation programs was recorded for 19,503 subjects at 6 months and was 47.73% (*n* = 9309) [34,36,37,41,42,44,50,53,55,64,66,67,68,71,72,74,79]; for 3772 subjects at 12 months and was 77.62% (*n* = 2928) [41,46,49,50,51,53,61,64,65,67,71,72,74,75]; and for 690 subjects at 15 months 55.36% (*n* = 382) [53,64,71].

The satisfaction rate was evaluated using the Client Satisfaction Questionnaire in 126 smokers, and the mean score was 29.25 [36,37,79].

No data on acceptability was registered.

Table 1 summarizes the population characteristics of the pharmacological technology-supported group and the non-pharmacological technology-supported group, as well as the related changes in human health.

Table 2 summarizes the effectiveness, adherence, satisfaction, and acceptability of the pharmacological technology-supported group and the non-pharmacological technology-supported group.

### 3.3. Pharmacological Therapy Smoking Cessation Interventions

A total of 39,367 adult daily smokers underwent a pharmacological technology-supported smoking cessation intervention and were included in the present umbrella review.

#### 3.3.1. Population Characteristics

The mean age was reported for 8136 smokers and was 30.16 years [33,34,41,43,44,45,47,50,51,53,56,59,61,64,66,67,69,71,72,74] and the gender was recorded for 20,485 smokers and was 1 male to 1.48 females (8271 males and 12,214 females) [32,33,34,36,37,41,43,44,45,47,50,51,53,61,64,66,67,71,72,74,79].

Of the 39,367 smokers, 11.14% (*n* = 4384) were hospitalized patients for not defined diseases [42,46]; 3.55% (*n* = 1396) were affected by HIV [34,36,37,43,50,52,53,64,67,68,71,79]; 3.22% (*n* = 1269) were affected by psychiatric disorders [34,47,59]; 1.84% (*n* = 725) were hospitalized patients pre not-defined surgery [46,57,65]; 1.75% (*n* = 690) were cancer survivors [53,64,71]; 0.47% (*n* = 184) were hospitalized cancer patients for pre resection surgery [57]; 0.41% (*n* = 162) were hospitalized for psychiatric disorders [34,47]; 0.24% (*n* = 96) were hospitalized for pre-orthopedic or general surgery [46,65]; and 0.06% (*n* = 42) were affected by chronic obstructive pulmonary diseases [64].

#### 3.3.2. Characteristics of Smoking Behaviors

The mean number of cigarettes smoked per day was registered before the smoking cessation intervention for 5112 smokers and was 15.64 cigarettes per day [41,47,50,51,53,61,64,66,71,72,74], while after the intervention, it was 7.69 for 1505 smokers [47,80].

To assess the nicotine severity dependence, the FTND and the HIS were used. The mean score of the FTND was 5.09 before the intervention for 2400 smokers [41,47,50,51,53,59,61,62,64,71,72,74,76], and 0.81 for 205 smokers after the intervention [34,47,50,53,64,71]; the HSI was used for 242 smokers, and the mean score was 2.71 before the intervention [33], while no data were available after the intervention.

#### 3.3.3. Health Status

No data on cardiovascular parameters were available before and after the smoking cessation interventions. However, cardiovascular complications occurred in 2% of 96 patients who stopped smoking before pre-orthopedic or general surgery [46,65].

As pneumological parameters, the mean ratio between the forced expiratory volume in one second (FEV1) and the forced vital capacity (FVC) registered at the end of the intervention of 42 patients affected by chronic obstructive pulmonary disease amounted to FEV1/FVC < 70% [64]. Furthermore, no pneumological complications occurred in 96 patients who stopped smoking before pre-orthopedic or general surgery [46,65].

As psychological parameters, the Beck Depression Inventory and the Brief Psychiatric Rating Scale were administered to 226 smokers and were significantly improved from baseline to 36 months, while the Short Form Survey on General Functioning and the Global Assessment of Functioning were not significantly improved at any time points [62,76].

No data on metabolic or oral parameters were evaluated before or after the smoking cessation interventions.

#### 3.3.4. Pharmacological Technology-Supported Intervention Characteristics and Outcomes

The smoking cessation interventions’ duration was 1 month for 268 (0.68%) smokers [34,53,64,71], 7 weeks for 731 (1.86%) [31], 2 months for 453 (1.15%) [31,36,37,46,65,79], 3 months for 2420 (6.15%) [42,49,52,53,56,64,68,69,70,71,72,74,80], 4 months for 3064 (7.78%) [47,66,80], 6 months for 3196 (8.12%) [31,32,33,43,44,47,53,64,71], and 12 months for 3970 (10.08%) [34,42,50,53,64,70,71,75]. For the other participants, the smoking cessation intervention duration was not specified.

The effectiveness of the smoking cessation interventions was evaluated as CARs or PPA, both self-reported and biochemically verified by cotinine or CO tests at different time points.

The effectiveness assessed as CARs self-reported was: CARs at 6 months was 1045 (7.60%) for 13,742 smokers [41,42,49,53,64,67,71,72]; CARs at 12 months was 54 (53.47%) for 101 smokers [45].

The effectiveness assessed as CARs biochemically verified by cotinine or CO tests was: 4 months CARs at 6 months was 1308 (57.17%) for 2288 smokers [32,33,43,44]; 5.5 months CARs at 6 months was 24 (19.05%) for 126 smokers [36,37,79]; 6 months CARs at 6 months was 264 (9.06%) for 2913 smokers [31,33,43,66,67]; 10 months CARs at 12 months was 1072 (46.85%) for 2288 smokers [32,33,43,44]; 12 months CARs at 12 months was 220 (8.51%) for 2585 smokers [31,34,37,41,56,79].

The effectiveness assessed as PPA self-reported was: 7 days PPA at 6 months was 2951 (15.78%) for 18,702 smokers [41,42,50,51,53,59,61,64,68,70,71,72,75]; 30 days PPA at 6 months was 1171 (25.06%) for 4673 smokers [34,42,53,59,64,71]; PPA at 12 months (time not defined) was 36 (37.50%) for 96 smokers [46,65]; 7 days PPA at 12 months was1519 (23.02%) for 6599 smokers [41,50,51,53,61,62,64,70,71,72,74,75,76,78,80]; 30 days PPA at 12 months was 76 (12.67%) for 600 smokers [42]; 30 days PPA at 15 months was 66 (9.57%) for 690 smokers [53,64,71]; 7 days PPA at 18 months was 18 (7.96%) for 226 smokers [62,76]; 7 days PPA at 24 months was 18 (7.96%) for 226 smokers [62,76]; 7 days PPA at 30 months was 18 (7.96%) for 226 smokers [62,76]; 7 days PPA at 36 months was 18 (7.96%) for 226 smokers [62,76].

The effectiveness assessed as PPA biochemically verified by cotinine or CO tests was: 7 days PPA at 6 months was 1064 (17.37%) for 6127 smokers [32,34,36,37,42,47,50,53,56,57,59,64,67,68,69,71,72,74,79,80]; 30 days PPA at 6 months was 597 (15.49%) for 3854 smokers [31,42,44,50,66]; 7 days PPA at 9 months was 60 (20.69%) for 290 smokers [34,50,53,64,71]; PPA at 12 months (time not defined) was 44 (19.82%) for 222 smokers [46,65]; 7 days PPA at 12 months was 489 (14.00%) for 3495 smokers [34,37,41,47,50,53,64,71,79,80]; 30 days PPA at 12 months was 69 (11.50%) for 300 smokers [42]; 7 days PPA at 18 months was 71 (18.25%) for 389 smokers [34,47].

Other effectiveness rates assessed as PPA but in which the methods of verification (self-reported/cotinine or CO test) were not reported were: PPA at 7 months was 11 (50.00%) for 22 smokers [70]; PPA at 12 months was 48 (20.00%) for 240 smokers [46,70]; 7 days PPA at 12 months was 64 (42.38%) for 151 smokers [46].

The adherence rate assessed as the number of subjects who completed the smoking cessation programs was recorded for 16,552 subjects at 6 months and was 41.37% (*n* = 6847) [34,36,37,41,44,50,53,64,67,68,71,72,74,79]; for 2401 subjects at 12 months and was 83.92% (*n* = 2015) [41,46,50,51,53,61,64,65,71,72,74]; and for 690 subjects at 15 months and was 55.36% (*n* = 382) [53,64,71].

The satisfaction rate was evaluated using the Client Satisfaction Questionnaire for 126 smokers and the mean score was 29.25. The satisfaction rate was evaluated using the Client Satisfaction Questionnaire for 126 smokers, and the mean score was 29.25 [36,37,79].

No data on acceptability was registered.

The Supplementary File S3 summarizes each pharmacological smoking cessation intervention performed and the related outcomes.

### 3.4. Non-Pharmacological Therapy Smoking Cessation Interventions

A total of 29,902 adult daily smokers underwent a non-pharmacological technology-supported smoking cessation intervention and were included in the present umbrella review.

#### 3.4.1. Population Characteristics

The mean age was reported for 13,373 smokers and was 42.27 years old [32,39,40,45,48,50,53,54,55,63,64,66,67,68,69,70,73,74]; the gender ratio was 1 male to 1.14 females and was reported for 13,787 subjects (6429 males and 7358 females) [32,35,39,40,45,46,48,49,50,53,55,63,64,66,67,68,69,70,74,75].

Of the 29,902 smokers, 12.85% (*n* = 3843) were hospitalized patients for not-defined diseases [42,53,64], 1.42% (*n* = 424) for cardiac diseases [31,42], 0.88% (*n* = 264) for pre not-defined surgery [31,46], and 0.42% (*n* = 125) for pre-cardiac surgery [46].

#### 3.4.2. Characteristics of Smoking Behaviors

The mean number of cigarettes smoked per day was registered before the smoking cessation intervention for 1524 smokers and was 19.84 cigarettes per day [35,45,63,66,67,68,70,75], while no data were available after the intervention.

To assess the nicotine severity dependence, the FTND was used. The mean score of the FTND was 3.92 before the intervention for 860 smokers [40,50,53,63,64,67,68,74], while no data were available after the intervention.

#### 3.4.3. Health Status

No data on cardiovascular, pneumological, psychological, metabolic or oral parameters were available before and after the smoking cessation interventions.

#### 3.4.4. Non-Pharmacological Technology-Supported Intervention Characteristics and Outcomes

The smoking cessation interventions’ duration was 4–6 weeks for 418 (1.40%) [66,75], 6 weeks for 652 (2.18%) [32,39,53], 7 weeks for 135 (0.45%) [31], 2 months for 246 (0.82%) [50,54,73], 3 months for 52 (0.18%) [70], 4 months for 30 (0.10%) [67], 6 months for 419 (1.40%) [31,42,80], and 12 months for 652 (2.18%) [40,42,46,50,53,64,74]. For the other participants, the smoking cessation intervention duration was not specified.

The smoking cessation interventions’ effectiveness was evaluated as CARs or PPA, both self-reported and biochemically verified by cotinine or CO tests at different time points.

The effectiveness assessed as CARs self-reported was: CARs at 6 months was 368 (16.37%) for 2248 smokers [58,75]; CARs at 12 months was 23 (4.17%) for 552 smokers [53].

The effectiveness assessed as CARs biochemically verified by cotinine or CO tests was: 4 months CARs at 6 months was 138 (31.94%) for 432 smokers [31,32,37]; 5 months CARs at 6 months was 22 (23.40%) for 94 smokers [31]; 6 months CARs at 6 months was 15 (14.85%) for 101 smokers [31,63]; 6 months CARs at 12 months was 20 (15.15%) for 132 smokers [64]; 10 months CARs at 12 months was 32 (58.18%) for 55 smokers [32]; 12 months CARs at 12 months was 68 (9.08%) for 749 smokers [31,63,77].

The effectiveness assessed as PPA self-reported was: PPA at 6 months (time not defined) was 40 (17.54%) for 228 smokers [49,67]; 2 days PPA at 6 months was 20 (9.80%) for 204 smokers [66]; 7 days PPA at 6 months was 1105 (24.65%) for 4483 smokers [31,38,42,44,50,53,54,64,66,67,70,73,75,80]; 30 days PPA at 6 months was 2436 (21.04%) for 11,577 smokers [42,45,60]; PPA at 12 months (time not defined) was 92 (11.12%) for 827 smokers [48,49,67,69,77]; 7 days PPA at 12 months was 434 (22.35%) for 1942 smokers [42,44]; 30 days PPA at 12 months was 171 (6.67%) for 1425 smokers [78]; 7 days PPA at 24 months was 285 (21.74%) for 1311 smokers [75].

The effectiveness assessed as PPA biochemically verified by cotinine or CO tests was: 2 days PPA at 6 months was 55 (13.75%) for 400 smokers [40,50,53,64,74]; 7 days PPA at 6 months was 160 (17.15%) for 933 smokers [31,35,38,42,45,53,55,63,64,66,67,68]; 30 days PPA at 6 months was 52 (15.48%) for 336 smokers [53,55,64,66]; 7 days PPA at 7 months was 24 for 100 smokers (24.00%) [32,39]; PPA at 12 months (time not defined) was 8 (7.27%) for 110 smokers [48,69]; 7 days PPA at 12 months was 175 (5.63%) for 3107 smokers [42,63,67,75,77].

Other effectiveness rates assessed as PPA but in which the methods of verification (self-reported/cotinine or CO test) were not reported were: PPA at 7 months was recorded for 52 smokers and amounted to 10 (19.23%) former smokers [70]; PPA at 12 months for 682 smokers and amounted to 165 (24.19%) former smokers [46,70,77]; 7 days PPA at 12 months for 145 smokers and amounted to 38 (26.21%) former smokers [46].

The adherence rate assessed as the number of subjects who completed the smoking cessation programs was recorded for 2951 subjects at 6 months and was 83.43% (*n* = 2462) [37,42,53,55,64,66,67,68]; for 1371 subjects at 12 months and was 66.59% (*n* = 913) [49,64,67,75].

No data on satisfaction or acceptability were registered.

The Supplementary File S3 summarizes each non-pharmacological smoking cessation intervention performed and the related outcomes.

### 3.5. Quality Assessment

The assessment of the risk of bias of the included systematic reviews [31,32,33,34,35,36,37,38,39,40,41,42,43,44,45,46,47,48,49,50,51,52,53,54,55,56,57,58,59,60,61,62,63,64,65,66,67,68,69,70,71,72,73,74,75,76,77,78,79,80] and the related quality was reported in Table S3 in Supplementary File S4.

## 4. Discussion

The present umbrella review primarily aimed to evaluate the long-term effectiveness (at least 6 months) of different pharmacological and non-pharmacological technology-supported smoking cessation interventions in 69,269 adults (≥18 years old) daily smokers of heated tobacco, of which 39,367 were involved in a pharmacological technology-supported program and 29,902 in a non-pharmacological one.

A total of 50 systematic reviews were included [31,32,33,34,35,36,37,38,39,40,41,42,43,44,45,46,47,48,49,50,51,52,53,54,55,56,57,58,59,60,61,62,63,64,65,66,67,68,69,70,71,72,73,74,75,76,77,78,79,80] comprising 46 different pharmacological and 32 non-pharmacological smoking cessation interventions. The related effectiveness was assessed heterogeneously, with variations in both methodology and timing, including self-reported or biochemically verified by cotinine or CO tests as CARs or PPA, at different time points. Thus, the possibility of conducting a meta-analysis to compare different subgroups of programs was precluded due to the heterogeneity of the smoking cessation interventions and their timing in the effectiveness assessment.

### 4.1. Population Characteristics

Attempts at smoking cessation were associated with population characteristics, such as sociodemographic factors, age, and level of education [81].

The mean age reported in the present umbrella review was 37.69 years old, particularly 30.16 in the pharmacological technology-supported smoking cessation group and 42.27 in the non-pharmacological one. The study’s population was restricted to adults (≥18 years old) daily smokers of heated tobacco to reduce the confounding variables associated with the population characteristics. Indeed, adolescents typically have sporadic smoking patterns often smoking only during the weekend or a few times every week compared to adults [82]. The assessment of smoking cessation effectiveness requires established and regular smoking behavior, which is in turn linked to a high risk of nicotine dependence, making behavioral changes more associated with the smoking cessation programs than with social influences as occur for adolescents [83]. Furthermore, due to the delayed manifestations of smoking on human health, adolescents demonstrated lower susceptibility to quitting smoking combined with the belief that they would be able to quit at any time [82]. In contrast, previous studies showed that only about 4% of adolescents between 12 and 19 years of age quit smoking successfully each year [82] and that smoking patterns in adolescents are characterized by frequent remissions and spontaneous relapses for a long time, which can make it difficult to assess smoking cessation programs’ effectiveness [83,84].

Despite the age restriction (≥18 years old) applied in the present study, the mean age reported (37.69 years) is relatively low. Comparing the reported mean age to previous studies that evaluated which age group had the highest proportion of using telemedicine services, our finding about the technology-supported smoking cessation interventions is in accordance with the study of Bhatia et al. [85], which registered that overall telemedicine use primarily occurred among 18 to 49 year-old subjects, even if the 50–70-year-old age group reported higher overall healthcare use in general [86].

Regarding the smokers’ gender ratio reported in the present umbrella review, a slightly higher proportion of females than males was reported (1 male to 1.33 females), both in the pharmacological technology-supported group (1 male to 1.48 females) and non-pharmacological (1 male to 1.14 females) group. Previous studies demonstrated that both males and females had similar quit rates and interest in quitting [87]. However, females reported a 30% lower rate than males of quitting successfully [87]. This could be partially explained by considering that nicotine replacement therapy (NRT) is less effective in females than males [88] due to the differences in smoking motivations: male smoking behavior is driven mainly by a higher nicotine pleasurable response than females, resulting in a better response to NRT; while female smoking behavior is driven mainly by unfavorable social and economic inequalities [87]. The investigated smoking cessation programs through the technology were in line with the suggestion to promote interventions as gender-neutral, also in addition to pharmacological therapy, to address socio-contextual influences and to improve quitting effectiveness among females [89]. The growing interest in gender-related discrepancies in the effectiveness of smoking cessation programs is highlighted by the inclusion of three systematic reviews [36,37,79] comprising studies targeting exclusively the female gender. Further gender studies, especially using gender-tailored smoking cessation programs and technology supports, could aid in implementing smoking cessation intervention designs in a gender-tailored manner [87].

Concerning the comorbidities reported in the study populations, it is interesting to note that subjects with psychiatric disorders were enrolled in pharmacological technology-supported programs (3.63% of subjects who underwent pharmacological programs had psychiatric disorders), while no psychiatric subjects were involved in the non-pharmacological group. This finding is in accordance with the WHO “Guidelines for the management of physical health conditions in adults with severe mental disorders”, which recommended a combination of pharmacological (NRT, bupropion, varenicline) and non-pharmacological smoking cessation interventions in subjects with severe mental illness [90]. Furthermore, the WHO declared that multimodal approaches including behavioral, pharmacological interventions and technological supports should be promising for subjects with mental illness, but were yet to be studied systematically [90]. The findings of the present umbrella review showed lower 7-day PPA at 6 months (17.37%) effectiveness rates of technology-supported multimodal pharmacological treatments in subjects with psychiatric disorders than the overall average of the investigated population. Finally, a comparison of the data recorded on the effectiveness biochemically verified as 7-day PPA at 6 months showed that the type of technological support did not appear to significantly influence the efficacy of the smoking cessation programs in psychiatric subjects. The 7-day PPA at 6 months recorded in subjects with mental illnesses was in fact between a minimum rate of 12.21% (technologically supported by telephone and quit line counselling) and 16.33% (technologically supported by a quit line only) (Supplementary File S3).

Another noteworthy finding is that, among the 22.73% of subjects with comorbidities in the pharmacological group, 14.1% were hospitalized, whereas in the non-pharmacological group, all the subjects affected with comorbidities (15.57%) were hospitalized. This could be related to the knowledge that hospitalization was considered a golden moment for smokers to try quitting; in fact, smoking cessation interventions started before an elective surgery registered higher effectiveness in previous studies [57,91].

### 4.2. Characteristics of Smoking Behaviors

Attempts at smoking cessation were associated with population characteristics, such as sociodemographic factors, age, and level of education, but also with the smoker’s status, particularly nicotine dependence [81]. Nicotine dependence assessment helps to define the smoking cessation plan and predict the associated risk of withdrawal symptoms [92]. An attempt to quit smoking has been linked to lower nicotine dependence [81].

The Fagerström Test for Nicotine Dependence (FTND) is a detailed, sensitive, and validated tool used to measure the level of nicotine dependence [93]. The weighted average FTND score registered in the smokers of the present study (4.78 assessed in 3260 smokers) before both pharmacological (5.09 assessed in 2400 smokers) and non-pharmacological (3.92 assessed in 860 smokers) technology-supported smoking cessation interventions was higher than other mean FTND recorded over the years in smokers of different countries, such as Norway and Germany (2.8 in the 1990s), USA (4.0 in the 1990s), and China (3.1 in 2013) [94,95]. This finding is in line with previous speculation, which stated that even if the prevalence of smokers decreased worldwide over the years, it was hypothesized that in future years, smokers might have higher smoking dependence scores because smokers with difficulties in quitting may continue to smoke [94].

The mean FTND score after the pharmacological technology-supported smoking cessation intervention was reduced from 5.09 to 0.81 (assessed in 205 smokers), while no data were recorded after the non-pharmacological one. This finding showed that pharmacological technology-supported smoking cessation was successful in reducing the average level of nicotine dependence from a value indicative of moderate nicotine dependence, tending toward strong dependence (values greater than six), to very low dependence (values less than three) [95].

The Heaviness of Smoking Index (HSI), which is used as a quick alternative to the FTND and was recommended to assess nicotine dependence for large-population-based studies due to its simplicity and quicker use [93], is based only on two main questions of the FTND (based on six questions) related to the number of cigarettes smoked per day and when smokers light their first cigarette after waking up [93]. In the present study, the HSI was used for 242 smokers of the pharmacological technology-supported smoking cessation intervention and the mean score was 2.71 before the intervention, a value also indicative of moderate nicotine dependence (HSI score 2–4 [96]), while no data were available after the intervention.

The number of cigarettes smoked per day is considered less reliable than the assessment of nicotine dependence due to the self-reported nature based on a single question [92]. Furthermore, the strength of cigarettes differs among brands, and nicotine metabolism varies among smokers [92].

A range of 11–20 cigarettes per day was associated with 1 point on the FTND test, with a minimum of 0 to 3 points for this question [95]. In the present study, considering the available findings reported for the pharmacological technology-supported intervention, the mean number of cigarettes smoked per day decreased from 15.64 (recorded for 5112 smokers) before the smoking cessation intervention to 7.69 (recorded for 1505 smokers) after the intervention.

These findings show the potential of pharmacological technology-supported smoking cessation intervention to reduce smoking among adult daily smokers with moderate nicotine dependence, switching from smoking about 15 cigarettes per day into low dependent light smokers (ten or fewer cigarettes smoked per day [92]), even when the smoker did not quit at the end of the intervention. Consequently, even if the program did not lead to success, it increased the likelihood of quitting smoking in the following years if further action and prevention measures were taken in a suitable timeframe. However, it should not be underestimated that light smokers experience nicotine withdrawal and that even a single symptom can compromise quitting [92,97].

### 4.3. Pharmacological and Non-Pharmacological Technology-Supported Intervention Characteristics

The WHO “Clinical treatment guideline for tobacco cessation in adults” [14] strongly recommends digital tobacco cessation interventions (such as mobile phone app, text-messages, or internet-based interventions) stand-alone or as support for tobacco users who want to quit smoking. In addition, the WHO strongly recommends pharmacological therapy for smokers interested in quitting, in particular varenicline, NRT, or bupropion, which are considered the first-line options [14].

This umbrella review showed that technological and pharmacological interventions were heterogeneously combined in 46 different interventions. Similarly, non-pharmacological technology-supported programs were heterogeneously combined with others in 32 different interventions. Despite the wide intervention diversity, the population is almost equally distributed between the groups in which the technology-supported smoking cessation interventions were associated (39,367; 56.83%) or not (29,902; 43.17%) with pharmacological therapy.

Among technological smoking cessation interventions, the WHO judged mobile phone text-messages as the technology with the strongest evidence currently [14], which was used in the present study in 5 of the 46 pharmacological technology-supported smoking cessation interventions and 4 of the 32 non-pharmacological ones.

Among the pharmacological therapy associated with technology interventions, only the pharmacological therapy considered first-line by the WHO was reported, while cytisine was never used in the included studies (Supplementary File S2). The WHO did not consider cytisine as a first-line medication even if its effectiveness is comparable, due to the related moderate evidence certainty; it is legally available in only a few countries and has greater doses and regimen variability [14].

In the present umbrella review, no traditional, complementary, or alternative therapy for smoking cessation interventions, such as acupuncture, laser therapy, hypnotherapy, yoga, traditional herbal medicine, or mindfulness meditation, were reported as support for the technology interventions. Moreover, in the clinical treatment guideline, the WHO judged the current evidence about traditional, complementary, and alternative therapies as insufficient to make a recommendation [14].

Concerning the smoking cessation intervention’s duration, despite the low number of included systematic reviews that specified this characteristic, pharmacological technology-supported programs tended to last longer (Table 2). In fact, there was an increasing rate of duration when considering 3 months (6.15%), 4 months (7.78%), 6 months (8.12%), and 12 months (10.08%). This should be partially linked with the recommended pharmacological duration of treatment, which was defined as 3 months for nicotine gum or lozenge; up to 6 months for nicotine nasal spray, nicotine oral inhaler, or bupropion; and 3–6 months for varenicline [98]. A small percentage of reported cases were also recorded at 7 weeks (1.86%), which matches the recommended duration of nicotine patch use (between 6 to 10 weeks) [98].

In contrast, technology-supported non-pharmacological interventions tend to have more flexibility in program duration, suggesting that the intervention duration may not be strictly defined and higher rates of shorter durations were registered than for pharmacological programs (Table 2).

### 4.4. Pharmacological vs. Non-Pharmacological Technology-Supported Smoking Cessation Effectiveness

The WHO considered both CARs and PPA assessed at least 6 months before the interventions’ starting, as critical long-term quit rates, an outcome crucial to decision-making for all smoking cessation interventions [14]. Biochemical verification of smoking cessation effectiveness was recommended in clinical trials when feasible to increase scientific rigor [99]. However, taking into account the biochemical verification limits related to higher costs and the need to organize an analysis plan to assess the biochemical measurements, self-reported abstinence was considered an adequate tool, especially in large-population-based trials [99]. As may be expected, the self-reported abstinence rates recorded in the present umbrella review are higher than those biochemically verified. The previous meta-analysis of Patrick et al. [100] compared the self-reported vs. the biochemically verified abstinence rates, showing that about 11% of self-reported former smokers were not confirmed biochemically as abstinent.

Analyzing and comparing the registered biochemically verified long-term PPA of the present umbrella review, it can be noted that the effectiveness rates are comparable between the pharmacological technology-supported smoking cessation interventions and the non-pharmacological ones: the 7 days PPA at 6 months was 17.37% vs. 17.15%; the 30 days PPA at 6 months was 15.49% vs. 15.48% at 6 months; and the 7 days PPA at 12 months was 14.00% vs. 5.63%, respectively.

Analyzing and comparing the registered biochemically verified long-term CARs of the present umbrella review, it can be noted that the effectiveness rates tend to be greater in the pharmacological group in the first treatment period, considering the first 4 months, but when considering the first 6 months, the trend reverses in favor of the non-pharmacological group, until leveling off at 12 months: the 4 months CARs at 6 months were 57.17% vs. 31.94%; the 6 months CARs at 6 months were 9.06% vs. 14.85% at 6 months; the 10 months CARs at 12 months were 46.85% vs. 58.18%; and the 12 months CARs at 12 months were 8.51% vs. 9.08%, respectively, for the pharmacological vs. non-pharmacological group.

**Figure 2:**
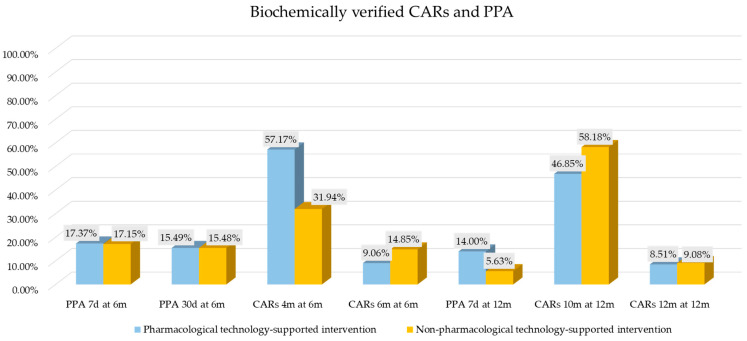
Biochemically verified long-term effectiveness assessed as CARs and PPA over time of pharmacological vs. non-pharmacological technology-supported smoking cessation interventions.

Figure 2 compares the biochemically verified effectiveness of pharmacological vs. non-pharmacological technology-supported smoking cessation interventions.

Biochemically verified PPA rates (both 7 days and 30 days) at 6 months of the pharmacological technology-supported group were similar to those in the non-pharmacological group, suggesting that both interventions were similarly effective in supporting abstinence at 6 months. Additionally, the 4-month CARs were significantly higher in the pharmacological group, indicating that medications had a greater impact on maintaining subjects’ continuous abstinence in the early stages. Moreover, as also mentioned above, all the recommended pharmacological treatment durations were completed within the first 6 months [101].

The significantly reduced abstinence 6-month CARs at 6 months compared to the 4-month CARs at 6 months for both the pharmacological and non-pharmacological groups could indicate that the first two months are those characterized by a higher rate of relapse, or that in the first two months, there would be a gradual reduction in the number of cigarettes smoked until reaching the first relapse.

At 12 months, the combined PPA and CARs data showed that both groups had similar rates of CARs, with the non-pharmacological group slightly higher. This suggests that, despite differences in short-term (7 days) abstinence rates of PPA, both smoking cessation strategies had comparable long-term effectiveness in terms of maintaining continuous abstinence.

The superiority of the pharmacological group at 12 months for PPA could indicate that pharmacological treatment was more effective in promoting short periods (7 days) of abstinence, but this difference did not translate into a lasting advantage in maintaining long-term CARs.

### 4.5. Adherence to Technological-Supported Smoking Cessation Interventions

Patients’ nonadherence is a clinical practice challenge that in smoking cessation programs contributes to lower effectiveness [102,103]. In the present umbrella review, the adherence rate assessed as the number of subjects who completed the smoking cessation programs was 47.73% at 6 months (assessed for 19,503 subjects), 77.62% at 12 months (assessed for 3772 subjects), and 55.36% at 15 months (assessed for 690 subjects).

The adherence rate at 6 months was higher in the non-pharmacological technology-supported smoking cessation group (83.43%) than the pharmacological one (41.37%). Data from several randomized controlled studies demonstrated the efficacy of NRT, varenicline, and bupropion in improving adherence to smoking cessation programs [19]. However, other population studies showed that pharmacotherapy may have had less adherence outside the clinical trials, probably due to the smoker’s failure to adhere to pharmacological recommendations in the “real world” as adequate doses and continued treatment, especially in the early phases [19]. Some reasons reported for nonadherence to pharmacological treatments included medication cost, adverse effects, and no patients’ perceived need to take drugs to quit smoking [19].

Comparing the adherence in relation to the continuous effectiveness of the pharmacological vs. non-pharmacological technology-based interventions at 6 months, the data showed that greater adherence (41.37% vs. 83.43%) was correlated with greater 6-month CARs biochemically verified effectiveness at 6 months (9.06% vs. 14.85%) for non-pharmacological interventions. A double interpretation of these results is possible: on the one hand, non-adherence could be the cause of the relapses; on the other hand, non-adherence could be the consequence of the relapses. By not considering this second option, called “reverse causality”, the risk is to overestimate the effect of adherence on the effectiveness [19]. Indeed, in the first case, non-adherence to the treatment would lead to treatment failure; whereas in the second case, relapse would lead to a mistrust of the treatment, resulting in a loss of adherence to it [19].

The adherence rate at 12 months was lower in the non-pharmacological technology-supported smoking cessation group (66.59%) than the pharmacological one (83.92%). The change in trend can be explained by the fact that the lower adherence rate for smoking cessation pharmacotherapies is reported in the early stages of treatment due to the onset of adverse effects. For example, for varenicline, a third of smokers were nonadherent in the second week of the pharmacological regimen [104].

Comparing the adherence in relation to the continuous effectiveness of the pharmacological vs. non-pharmacological technology-based interventions at 12 months, the data showed that lower adherence (66.59% vs. 83.92%) was correlated with greater 12-month CARs biochemically verified at 12 months effectiveness (8.51% vs. 9.08%).

Despite the lower adherence in the non-pharmacological group, treatment continuous effectiveness remains relatively higher, suggesting that in the long term, the quality and constancy of adherence found in the non-pharmacological group (6-month adherence of 83.54% and 12-month adherence of 66.59%) may be a more important factor than the quantity and inconstancy of adherence found in the pharmacological group.

**Figure 3:**
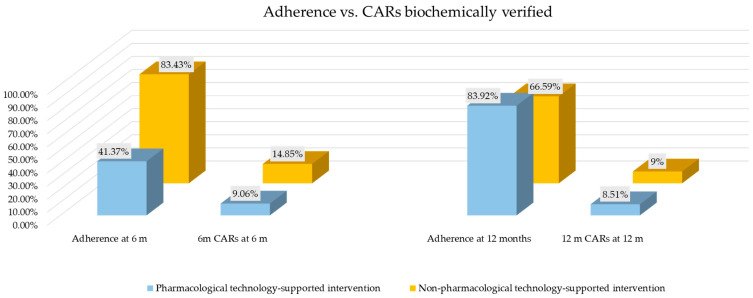
Adherence vs. CARs biochemically verified over time of pharmacological vs. non-pharmacological technology-supported smoking cessation interventions.

Specifically, Figure 3 compares the biochemically verified effectiveness of pharmacological vs. non-pharmacological technology-supported smoking cessation interventions.

### 4.6. Satisfaction with Technology-Supported Smoking Cessation Interventions

The results of the present umbrella review show that the satisfaction rate was evaluated using the Client Satisfaction Questionnaire (CSQ) in 126 smokers [36,37,79] involved in pharmacological technology-supported smoking cessation interventions, while no data on satisfaction were registered for non-pharmacological ones. The mean CSQ score was 29.25 [36,37,79], indicating high satisfaction.

In particular, considering the study [105] that assessed the CSQ values, the mean CSQ score registered for the in-person counseling plus pharmacological therapy plus video counseling group was 29.6 vs. 28.9 registered for the in-person counseling plus pharmacological therapy plus telephone counseling (Supplementary File S1) [36,37,79]. The difference in the average CSQ scores was not statistically different [105]. Likewise, the difference in the adherence rate at 6 months was not statistically different between the video vs. telephone counseling groups (66.67% vs. 61.90%, respectively) [105].

In contrast, the 5.5 months CARs and the 7 days PPA verified by cotinine test at 6 months were statistically different between the video vs. the telephone groups (33.33% and 38.10% vs. 4.76% and 4.76%, respectively) (Supplementary File S1) [105].

Despite the small sample size, these findings suggest that video counseling had significantly higher effectiveness rates than telephone counseling, when combined with in-person counseling and pharmacological therapy, despite similar satisfaction and adherence rates between the two groups.

### 4.7. Smoking Cessation and Related Benefits on Human Health

The WHO estimates that there are 1.3 billion tobacco smokers around the world, and half of them die due to tobacco-related noncommunicable diseases [2], such as cardiovascular diseases (e.g., heart failure and stroke) [3], pneumological diseases (e.g., asthma, chronic obstructive pulmonary disease, and lung cancer) [4], and oral diseases (e.g., periodontal and peri-implant disease, oral potentially malignant disorders, and oral cancer) [6].

#### 4.7.1. Cardiovascular and Pneumological Health

Former smokers were associated with significantly lower cardiovascular disease risk than smokers [106]. However, the cardiovascular risk of former smokers should be compared to the never-smokers’ risk after 5–10 years since the smoking cessation [107], considering that the former smokers’ risk was still linked with the previous cumulative amount of smoked cigarettes per day and the years since quitting [106]. Smoking was also a risk factor found in about 15–20% affected by chronic obstructive pulmonary disease and in 85% of lung cancer patients [108,109], and smoking cessation was considered a key intervention to slow down the lung function decline [109].

No data on cardiovascular or pneumological parameters were available both before and after the smoking cessation interventions in the present umbrella review. However, two included systematic reviews [46,65] reported the findings of Lindström et al. [91], which recorded that cardiovascular or pneumological complications occurred respectively in 2% and 0% of 96 patients who stopped smoking before pre-orthopedic or general surgery [46,65]. The low incidence of complications could be partially explained by considering the higher oxygen delivery in a former smoker to the healing of tissue, as if pre-operative smoking cessation operated as an oxygen supplement therapy [91].

It is interesting to note how the study of Lindström et al. [91], using a pharmacological technology-supported smoking cessation intervention, reported a higher smoking cessation PPA self-reported effectiveness at 12 months (37.50%) compared to the mean 7 days and 30 days PPA self-reported effectiveness (23.02% and 12.67% respectively) of the pharmacological technology-supported smoking cessation intervention. The higher effectiveness should be reflected in the motivation to quit smoking prior to an elective surgery [91]. Thus, smoking cessation interventions seem more effective when started before surgery, and a pre-operative intervention may be a golden moment to achieve a higher effectiveness rate maintained at the 12-month follow-up [91].

#### 4.7.2. Mental Health

It is a common opinion that smoking can aid in stress management and may serve as a “self-medication” for subjects with mental health disorders [10]. However, biological explanations suggest that smoking may exacerbate mental health disorders [10]. Smoking leads to neuroadaptive changes, which can result in frequent nicotine withdrawal symptoms such as depression, anxiety, and irritability [10]. In this context, quitting smoking showed improvement effects in mental health, rather than making it worse [10,76].

In line with these findings on the role of smoking cessation on mental health, two included systematic reviews [62,76] reported the findings of Baker et al. [110], which administered the Beck Depression Inventory and the Brief Psychiatric Rating Scale to smokers with severe mental illness (such as schizophrenia spectrum, bipolar disorders, and nonorganic psychotic syndrome) and found a significant improvement from baseline to 36 months.

In contrast, the Short-Form Survey on General Functioning and the Global Assessment of Functioning (GAF) were not significantly improved at any time point [110]. However, in interpreting this result, it should be taken into account that the GAF (a tool used primarily in psychiatric patients to measure the level of psychological, social, and occupational functioning) was removed from the Diagnostic and Statistical Manual of Mental Disorders-V version published in 2013 in favor of other assessment tools [111]. Limitations ascribed to the GAF related to its ability to provide a comprehensive assessment as a single score may not sufficiently capture the full extent of the diagnosis, severity of symptoms, risk to self or others, or impairment of social functioning and self-care. These aspects may fluctuate independently over time [111].

#### 4.7.3. Oral Health

The oral mucosa is directly exposed to heated tobacco, which is a risk factor for periodontitis, peri-implantitis, and alveolar osteitis, as well as oral potentially malignant disorders and oral cancer [6]. Smoking was recognized as one of the main risk factors and as a grade modifier of periodontitis [112,113,114]. However, the molecular and biological mechanisms associated with the negative effect of smoking on periodontal and peri-implant status, and how smoking cessation could improve these periodontal parameters, are still lacking [112,113]. Therefore, a secondary objective of the present study was to evaluate periodontal changes following smoking cessation programs, but unfortunately, no oral data were found.

In contrast to periodontitis, the effect of smoking cessation on oral cancer risk is better known [6,115]. Smokers have a 5–10 times higher risk of developing oral cancer, which is reduced by half of the initial risk after 5 years of smoking cessation, to a risk equivalent to that of non-smokers after 10 years. Despite this knowledge, a recent study estimated that smoking was found in approximately 76% of oral cancer patients [115].

### 4.8. Clinical Practice Implications

The findings of the present umbrella review highlighted that both pharmacological and non-pharmacological technology-supported smoking cessation interventions had comparable long-term effectiveness, meaning that decision-making for these kinds of smoking cessation programs should be guided by smokers’ characteristics rather than an assumed superiority of one program.

Pharmacological technology-supported smoking cessation interventions should be prioritized for highly nicotine-dependent smokers [95], as they were associated with higher CARs rates in the early months, suggesting a crucial role in managing withdrawal symptoms and preventing early relapse. However, clinicians should actively improve adherence using the technology-supported systems as reminder and monitoring tools more intensively. In fact, non-adherence to pharmacological treatments is a key barrier to success. Clinicians should improve proactive adherence monitoring especially in the first required 3–6 months minimum recommended pharmacological treatment duration, when relapse rates are the highest. Furthermore, in line with the phenomenon of the “reverse causality”, early dropout from pharmacological treatments were also related to perceived lack of effectiveness or pharmacological side-effects. Behavioral in-person or based on technology-supported systems counseling on realistic expectations, and monitoring through technology of the side effects, should be integrated into clinical practice to recognize and manage at an early stage the side effects that may be the cause of some failures in smoking cessation attempts.

Non-pharmacological technology-supported smoking cessation interventions should be prioritized for smokers with previous failed quit attempts caused by non-adherence, pharmacological intense side effects, concerns about costs, or personal preferences. In fact, non-pharmacological technology-supported interventions showed similar effectiveness in the long-term with lower costs and shorter treatment durations compared to the pharmacological technology-supported interventions, which required higher drug-related costs and were less flexible due to the required 3–6 months minimum recommended treatment duration.

Finally, given that both pharmacological and non-pharmacological technology-supported interventions were effective, insurance plans and public health programs should offer financial support for digital smoking cessation interventions, which can provide a cost-efficient strategy for healthcare providers, allowing remote support without the need for frequent in-person appointments. Technology-supported smoking cessation interventions should be routinely integrated into clinical practice to improve treatment adherence to accommodate individual patient preferences and behavioral support, particularly for smokers with limited access to in-person behavioral support due to geographical or logistical barriers.

However, as a potential limitation of technology-supported smoking cessation interventions, it should be considered that in smokers with psychiatric comorbidities, the findings of the present umbrella review suggested that technology-supported multimodal pharmacological treatments showed lower effectiveness rates compared to the general population. Although the type of technological tools did not appear to significantly influence effectiveness within this specific group, it is crucial to acknowledge the potential lower overall effectiveness. Therefore, for patients with psychiatric disorders, adopting an even more personalized and intensive approach is essential. This could include more frequent and proactive monitoring of adherence, careful management of pharmacological side effects, and the integration of robust behavioral support tailored to their specific needs. The choice of the type of technological support should be guided by patient preferences and the availability of resources, keeping in mind that effectiveness may be lower compared to other patient groups.

### 4.9. Strengths, Limitations, and Future Prospectives

Based on the best of our knowledge of current evidence, the present umbrella review is a pioneer in assessing and comparing the long-term effectiveness (at least 6 months) of pharmacological vs. non-pharmacological technology-supported smoking cessation interventions and related adherence and satisfaction. The inclusion of 69,269 adult daily smokers of heated tobacco enhances the generalizability of the findings, offering a comprehensive representation of the target population and strengthening the reliability of the conclusions. Furthermore, despite the large sample size, it is important to note that several potential confounders were the exclusion criteria, such as younger age (<18 years old), not daily smokers of heated tobacco, pregnant or lactating women, subjects drinking alcohol, or those who had other substance abuse disorders. This approach ensures that the results of the present umbrella review are focused on a clear target population, reducing the confounding population variables.

Another strength of the present umbrella review is its focus on a current topic, ensuring that the included studies are up-to-date and reflect the latest research in the field. As predictable, even if the search strategy did not impose any restrictions on the article’s years of publication, all the included systematic reviews [31,32,33,34,35,36,37,38,39,40,41,42,43,44,45,46,47,48,49,50,51,52,53,54,55,56,57,58,59,60,61,62,63,64,65,66,67,68,69,70,71,72,73,74,75,76,77,78,79,80] were published in relatively recent years: in the last 16 years (from 2009 to 2024). The considerable number of systematic reviews on technology-supported smoking cessation interventions published in the past 16 years highlights the growing role of technology and reflects the increasing interest in exploring new technological frontiers utilized for public health purposes [116,117].

On the other hand, some limitations should be taken into account when interpreting the findings of the present umbrella review. Considering the quality of the included studies, the AMSTAR-2 tool showed that although 44.00% of the systematic reviews were classified as high or moderate quality, a significant proportion (56.00%) had low or critically low quality (Supplementary File S3). This raises concerns about the potential risk of bias that may undermine the strength of the conclusions’ evidence.

The lack of standardized intervals of follow-up generated heterogeneity in the smoking cessation effectiveness assessment at each time point, allowing the qualitative synthesis of data, but precluding the parallel comparison between the several subtypes of pharmacological and non-pharmacological smoking cessation interventions retrieved. In addition, the methodological heterogeneity of the effectiveness assessment (PPA/CARs and self-reported/biochemically verified) precluded quantitative analysis of the results.

Unfortunately, the current data available in the literature did not allow for the achievement of the other secondary aims of the present umbrella review, established in the drafting of the study protocol before starting the research, such as the evaluation and comparison of the long-term effectiveness on medical (cardiovascular, pneumological, metabolic, and psychological), and oral (periodontal and peri-implant, mucosal lesions) parameters before and after pharmacological vs. non-pharmacological technology-supported smoking cessation interventions, as well as the related programs’ acceptability.

It might be conceivable that greater acceptability of smoking cessation programs could potentially enhance their effectiveness, as higher user engagement and adherence. Therefore, the lack of data on acceptability limits our understanding of the impact of the users’ engagement on smoking cessation programs’ effectiveness.

Looking ahead, future research should focus on assessing the acceptability of both pharmacological and non-pharmacological technology-supported smoking cessation interventions. Exploring user acceptability should be the key to optimizing and implementing the smoking cessation programs’ design.

Finally, it would be valuable to investigate the long-term human health benefits, including improvements in medical (cardiovascular, pulmonary, and psychological) and oral (periodontal and peri-implant, mucosal lesions) parameters to provide a more comprehensive understanding of the overall impact of the technology-supported smoking cessation interventions on health and wellbeing, filling the knowledge gaps on the perspectives of cigarette smoking cessation on human health, such as concerning periodontal disease.

## 5. Conclusions

Smoking cessation pharmacological and non-pharmacological technology-supported interventions in adult daily smokers of tobacco showed similar biochemically verified PPA rates (7 day PPA was 17.37% vs. 17.15%) and CARs (9.06% vs. 14.85%) at 6 months, while at 12 months the PPA trend reversed (7 day PPA was 14.00% vs. 5.63%) but remained similar for CARs (8.51% vs. 9.08%).

These findings suggested that both interventions were similarly effective in supporting abstinence at 6 months. Additionally, the 4-month CARs were significantly higher in the pharmacological group, indicating that medications had a greater impact on maintaining subjects’ continuous abstinence in the early stages, excluding the first two months, probably characterized by a higher rate of relapse, or by a gradual reduction in the number of cigarettes smoked until reaching the first relapse.

At 12 months, both smoking cessation strategies had comparable long-term effectiveness in terms of maintaining continuous abstinence. Thus, the superiority of the pharmacological group at 12 months for PPA could indicate that pharmacological treatment was more effective in promoting short periods (7 days) of abstinence, but this difference did not translate into a lasting advantage in maintaining long-term CARs. However, these finding concerning the effectiveness over time should be interpreted considering the heterogeneity of follow-up intervals across studies, which may have influenced the assessment of the effectiveness at different time points.

In adult daily smokers who did not achieve abstinence, the pharmacological technology-supported smoking cessation intervention allowed for a reduction in the smokers’ moderate nicotine dependence and smoking about 15 cigarettes per day into low-dependent light smokers (ten or fewer cigarettes smoked per day). Consequently, even if the program did not lead to success, it increased the likelihood of quitting smoking in the following years if further action and prevention measures were taken within a reasonable timeframe.

Nonadherence to treatment is a significant challenge in smoking cessation programs, impacting effectiveness. In this umbrella review, adherence at 6 months was higher in the non-pharmacological group (83.43%) compared to the pharmacological group (41.37%), correlating with higher CARs. However, due to reverse causality, non-adherence could be both a cause and a consequence of pharmacological treatment failure, complicating the interpretation of adherence’s effect on outcomes.

At 12 months, despite lower adherence in the non-pharmacological group (66.59%) compared to the pharmacological group (83.92%), the non-pharmacological group showed comparable or higher long-term effectiveness. These findings suggest that the quality and consistency of adherence to non-pharmacological interventions may be more important for sustained success than the quantity and variability of adherence in pharmacological treatments. However, the heterogeneity in the follow-up intervals across studies to assess smoking cessation effectiveness was further accompanied by variability in the adherence assessment intervals. Consequently, the relationship between adherence and effectiveness must be interpreted with caution, as it is influenced by variations in the timing of assessments across the included studies.

Thus, non-pharmacological technology-supported interventions showed similar effectiveness at 12 months with lower costs and shorter treatment durations compared to the pharmacological technology-supported interventions, which had higher drug-related costs and were less flexible due to the required 3–6 months minimum recommended treatment duration. However, the lack of data on program acceptability prevents a full understanding of how user engagement influences intervention effectiveness.

Additionally, in the overall interpretation of these findings, it should be considered that a significant proportion (56.00%) of the studies included in this systematic review had low or critically low quality. This raises concerns about the potential risk of bias that may undermine the strength of the evidence supporting the conclusions.

Looking ahead, future high-quality research should focus on assessing the acceptability and long-term human health benefits of both pharmacological and non-pharmacological technology-supported smoking cessation interventions for understanding the overall impact of technology-supported smoking cessation interventions on health and well-being, filling the knowledge gaps.

## Figures and Tables

**Table 1:** Population characteristics.

	Pharmacological Technology-Supported Smoking Cessation Intervention		Non-Pharmacological Technology-Supported Smoking Cessation Intervention		Total	
Sample size	39,367		29,902		69,269	
Mean age	30.16 y.o. (reported for 8136/39,367)		42.27 y.o. (reported for 13,373/29,902)		37.69 y.o. (reported for 21,509/69,269)	
Gender ratio	1 M/1.48 F 8271 M/12,214 F (reported for 20,485/39,367)		1 M/1.14 F 6429 M/7358 F (reported for 13,787/29,902)		1 M/1.33 F 14,700 M/19,572 F (reported for 34,272/69,269)	
Comorbidities						
Cancer survivors	690 (1.75%)		-		690 (1.00%)	
Chronic obstructive pulmonary diseases	42 (0.11%)		-		42 (0.06%)	
HIV	1396 (3.55%)		-		1396 (2.02%)	
Psychiatric disorders	1269 (3.22%)		-		1269 (1.83%)	
Hospitalized for N/D diseases	4384 (11.14%)		3843 (12.85%)		8227 (11.88%)	
Hospitalized for psychiatric disorders	162 (0.41%)		-		162 (0.23%)	
Hospitalized for cardiac diseases	-		424 (1.42%)		424 (0.61%)	
Hospitalized for N/D pre-surgery	725 (1.84%)		264 (0.88%)		989 (1.43%)	
Hospitalized for pre-cardiac surgery	-		125 (0.42%)		125 (0.18%)	
Hospitalized for pre-orthopedic or general surgery	96 (0.24%)		-		96 (0.14%)	
Hospitalized cancer patients for pre-resection surgery	184 (0.47%)		-		184 (0.27%)	
	Before	After	Before	After	Before	After
Smoked cigarettes/day (mean)	15.64 (reported for 5112/39,367)	7.69 (reported for 1505/39,367)	19.84 (reported for 1524/29,902)	-	16.60 (reported for 6636/69,269)	7.69 (reported for 1505/69,269)
FTND (mean)	5.09 (reported for 2400/39,367)	0.81 (reported for 205/39,367)	3.92 (reported for 860/29,902)	-	4.78 (reported for 3260/69,269)	0.81 (reported for 205/69,269)
HSI (mean)	2.71 (reported for 242/39,367)	-	-	-	2.71 (reported for 242/39,367)	-

Abbreviations: years old, “y.o”; male, “M”; female, “F”; not defined, “N/D”; Fargström Test for Nicotine Dependence, “FTND”; Heaviness Smoking Index, “HSI”; Human Immunodeficiency Virus, “HIV”.

**Table 2:** Smoking cessation intervention effectiveness, adherence, satisfaction, acceptability.

	Pharmacological Technology-Supported Smoking Cessation Intervention	Non-Pharmacological Technology-Supported Smoking Cessation Intervention	Total
Sample size	39,367 (56.83%)	29,902 (43.17%)	69,269
Intervention Duration			
1 month	268 (0.68%)	-	268 (0.39%)
4–6 weeks	-	418 (1.40%)	418 (0.60%)
6 weeks	-	652 (2.18%)	652 (0.94%)
7 weeks	731 (1.86%)	135 (0.45%)	866 (1.25%)
2 months	453 (1.15%)	246 (0.82%)	699 (1.01%)
3 months	2420 (6.15%)	52 (0.18%)	2472 (3.57%)
4 months	3064 (7.78%)	30 (0.10%)	3094 (4.47%)
6 months	3196 (8.12%)	419 (1.40%)	3615 (5.22%)
12 months	3970 (10.08%)	652 (2.18%)	4622 (6.67%)
Not defined	25,265 (64.18%)	27,298 (91.29%)	52,563 (75.88%)
Effectiveness			
CARs self-reported: Number of Former smokers/Number of Smokers tested (% of former smokers)			
For 6 m at 6 m	1045/13,742 (7.60%)	368/2248 (16.37%)	1413/15,990 (8.84%)
For 12 m at 12 m	54/101 (53.47%)	23/552 (4.17%)	77/653 (11.79%)
CARs biochemically verified: Number of Former smokers/Number of Smokers tested (% of former smokers)			
For 4 m at 6 m	1308/2288 (57.17%)	138/432 (31.94%)	1446/2720 (53.16%
For 5 m at 6 m	-	22/94 (23.40%)	22/94 (23.40%)
For 5.5 m at 6 m	24/126 (19.05%)	-	24/126 (19.05%)
For 6 m at 6 m	264/2913 (9.06%)	15/101 (14.85%)	279/3014 (9.26%)
For 6 m at 12 m	-	20/132 (15.15%)	20/132 (15.15%)
For 10 m at 12 m	1072/2288 (46.85%)	32/55 (58.18%)	1104/2343 (47.12%)
For 12 m at 12 m	220/2585 (8.51%)	68/749 (9.08%)	288/3334 (8.64%)
PPA self-reported: Number of Former smokers/Number of Smokers tested (% of former smokers)			
6 m (time N/D)	-	40/228 (17.54%)	40/228 (17.54%)
2 d at 6 m	-	20/204 (9.80%)	20/204 (9.80%)
7 d at 6 m	2951/18,702 (15.78%)	1105/4483 (24.65%)	4056/23,185 (17.49%)
30 d at 6 m	1171/4673 (25.06%)	2436/11,577 (21.04%)	3607/16,250 (22.20%)
7 d at 7 m	-	-	-
12 m (time N/D)	36/96 (37.5%)	92/827 (11.12%)	128/923 (13.87%)
7 d at 12 m	1519/6599 (23.02%)	434/1942 (22.35%)	1953/8541 (22.87%)
30 d at 12 m	76/600 (12.67%)	95/1425 (6.67%)	171/2025 (8.44%)
30 d at 15 m	66/690 (9.57%)	-	66/690 (9.57%)
7 d at 18 m	18/226 (7.96%)	-	18/226 (7.96%)
7 d at 24 m	18/226 (7.96%)	285/1311 (21.74%)	303/1537 (19.71%)
7 d at 30 m	18/226 (7.96%)	-	18/226 (7.96%)
7 d at 36 m	18/226 (7.96%)	-	18/226 (7.96%)
PPA biochemically verified: Number of Former smokers/Number of Smokers tested (% of former smokers)			
2 d at 6 m	-	55/400 (13.75%)	55/400 (13.75%)
7 d at 6 m	1064/6127 (17.37%)	160/933 (17.15%)	1224/7060 (17.34%)
30 d at 6 m	597/3854 (15.49%)	52/336 (15.48%)	649/4190 (15.49%)
7 d at 7 m	-	24/100 (24.00%)	24/100 (24.00%)
7 d at 9 m	60/290 (20.69%)	-	60/290 (20.69%)
12 m (time N/D)	44/222 (19.82%)	8/110 (7.27%)	52/332 (15.66%)
7 d at 12 m	489/3495 (14.00%)	175/3107 (5.63%)	664/6602 (10.06%)
30 d at 12 m	69/600 (11.50%)	-	69/600 (11.50%)
30 d at 15 m	-	-	-
7 d at 18 m	71/389 (18.25%)	-	71/389 (18.25%)
7 d at 24 m	-	-	-
7 d at 30 m	-	-	-
7 d at 36 m	-	-	-
PPA methods N/D: Number of Former smokers/Number of Smokers tested (% of former smokers)			
7 m (time N/D)	11/22 (50.00%)	10/52 (19.23%)	21/74 (28.38%)
12 m (time N/D)	48/240 (20.00%)	165/682 (24.19%)	213/922 (23.10%)
7 d 12 m	64/151 (42.38%)	38/145 (26.21%)	102/296 (34.46%)
Adherence Number of subjects who completed the smoking cessation program/Number of smokers tested (% of adherent subjects)			
At 6 m	6847/16,552 (41.37%)	2462/2951 (83.43%)	9309/19,503 (47.73%)
At 12 m	2015/2401 (83.92%)	913/1371 (66.59%)	2928/3772 (77.62%)
At 15 m	382/690 (55.36%)	-	382/690 (55.36%)
Satisfaction			
Client Satisfaction Questionnaire (mean score)	29.25 (reported for 126/39,367)	-	29.25 (reported for 126/69,269)
Acceptability			
	-	-	-

Abbreviations: days, “d”; months, “m”; Continuous Abstinence Rates, “CARs”; Point Prevalence Abstinence, “PPA”; not defined, “N/D”; missing data, “-”.

## Data Availability

Data are available in the MEDLINE/PubMed, Web of Science, Scopus, and BioMed databases, and in the PROSPERO register.

## Supplementary Materials

The following supporting information can be downloaded at: https://www.mdpi.com/article/10.3390/healthcare13080953/s1, File S1: Data Extraction Table; File S2: Smoking cessation intervention characteristics; File S3: Pharmacological and Non-Pharmacological Technology-supported Smoking Cessation Interventions; File S4: Quality Assessment.
