# Supplementary material for: Effectiveness and Adherence of Pharmacological vs. Non-Pharmacological Technology-Supported Smoking Cessation Interventions: An Umbrella Review
Source: Healthcare (Basel). 2025 Apr 21;13(8):953. doi: 10.3390/healthcare13080953 (PMC12027406; doi:10.3390/healthcare13080953)
Supplement: Supplementary file 1 [file healthcare-13-00953-s001.zip › Supplementary File S2 Smoking cessation intervention characteristics.pdf]

Supplementary File S2: Smoking Cessation Interventions Characteristics

The Table S2 shows the technology-supported smoking cessation intervention characteristics reported in each included systematic review (alphabetically ordered).

Table S2. Technology-supported smoking cessation interventions. Sample size and smoking intervention type (pharmacological/not-pharmacological therapy), intervention characteristics, intervention frequency and duration.

Abbreviations: number, "n."; Pharmacological Therapy "PT"; Missing Data, "MD"; Not Derivable, "N/D"; Interactive Voice Response, "IVR"; Nicotine Replacement Therapy, "NRT".

Study	Sample size and Smoking intervention	Intervention Characteristics	Frequency	Duration
Akanbi M.O.,	n.53	In-person counseling	9 counseling of 15 minutes each	>6 months
2019	PT	Pharmacological therapy (bupropion)	MD	MD
		Telephone counseling	2 counseling	>6 months (concurrent)
[31]	n.53	In-person counseling	9 counseling of 15 minutes each	>6 months
	PT	Pharmacological therapy (nortriptyline)	MD	MD
		Telephone counseling	2 counseling	>6 months (concurrent)
	n.51	In-person counseling	9 counseling of 15 minutes each	>6 months
	Not PT	Telephone counseling	2 counseling	>6 months (concurrent)
	n.134	In-person counseling	3 counseling of 30 minutes each	>7 weeks
	PT	NRT (patches)	MD	6 weeks
		Telephone counseling	5 counseling of 10 minutes each	>7 weeks (concurrent)
	n.135	In-person counseling	3 counseling of 30 minutes each	>7 weeks
	Not PT	Telephone counseling	5 counseling of 10 minutes each	>7 weeks (concurrent)
	n.111	In-person counseling	6 counseling	>2 months
	PT	NRT (gum)	MD	2 weeks
		Telephone counseling	2 counseling of 10-15 minutes each	>2 months (concurrent)
	n.120	In-person counseling	6 counseling	>2 months
	PT	NRT (gum)	MD	2 weeks
		Telephone counseling	6 counseling of 10-15 minutes each	>2 months (concurrent)
	n.94	In-person counseling	1 counseling of 20 minutes	MD
	Not PT	Telephone counseling	9 counseling of 10 minutes each	MD
	n.141	In-person counseling	1 counseling of 30 minutes	MD
	Not PT	Telephone counseling	7 counseling of 10 minutes each	>6 months
	n.160	In-person counseling by cardiologists	2 counseling of 10-45 minutes each	MD
	Not PT	Telephone counseling by cardiologists	15 counseling	MD
	n.160	In-person counseling by cardiologists	2 counseling of 10-45 minutes each	MD

	Not PT	Telephone counseling by cardiologists	15 counseling	MD
	n.181<br>Not PT	In-person counseling<br>Telephone counseling	1 counseling of 1 minute<br>5 counseling of 1 minute	MD<br>>12 months
Barroso-Hurtado M., 2021<br><br>[32]	n.285<br>PT	In-person counseling<br>Mobile app (CureApp smoking cessation)<br>NRT<br>Chatbot<br>Mobile carbon monoxide checker	5 counseling<br>MD<br>MD<br>MD<br>MD	12 weeks (concurrent)<br>24 weeks (concurrent)<br>MD<br>24 weeks (concurrent)<br>24 weeks (concurrent)
	n.287<br>PT	In-person counseling<br>NRT<br>Mobile app (CureApp smoking cessation)	5 counseling<br>MD<br>MD	12 weeks (concurrent)<br>MD<br>24 weeks (concurrent)
	n.50<br>Not PT	In-person group counseling<br>Mobile phone App (SmartQuit)	90 minutes each<br>MD	6 weeks<br>MD
	n.20<br>PT	In-person counseling<br>Pharmacological therapy<br>Telephone counseling<br>Mobile phone App (mCM app)	7 counseling<br>MD<br>1 counseling<br>MD	MD<br>MD<br>MD<br>MD
	n.55<br>Not PT	In-person counseling<br>Mobile phone App (CASC)	MD<br>MD	MD<br>MD
Bendotti H., 2023<br><br>[33]	n.242<br>PT	Pharmacological therapy<br>Chatbot	MD<br>Daily contact and at any time based on the patient's choice	MD<br>MD
	n.285<br>Not PT	In-person counseling<br>Mobile app (CureApp smoking cessation)<br>NRT<br>Chatbot<br>Mobile carbon monoxide checker	5 counseling<br>MD<br>MD<br>MD<br>MD	12 weeks (concurrent)<br>24 weeks (concurrent)<br>MD<br>24 weeks (concurrent)<br>24 weeks (concurrent)
	n.287<br>PT	In-person counseling<br>NRT<br>Mobile app (CureApp smoking cessation)	5 counseling<br>MD<br>MD	12 weeks (concurrent)<br>MD<br>24 weeks (concurrent)
Boland C.V., 2018<br><br>[34]	n.58<br>PT	NRT (smokers of 5 or more cigarettes)<br>Computer-based internet counseling	MD<br>5 counseling	Up to 10 weeks<br>Up to 12 months
	n.113<br>PT	In-person counseling<br>NRT (patches)<br>Computer-delivered tailored printed materials<br>Telephone counseling	2 counselling 90 minutes each<br>MD<br>MD<br>16 counseling 10 minutes each	MD<br>10 weeks<br>At 3 and 6 months<br>At 3 and 6 months
	n.280<br>PT	NRT<br>Printed materials<br>Computer-based internet video counseling (Polycom PVX)	MD<br>MD<br>4 counseling at baseline, 1, 4 and 8 week	MD<br>MD<br>8 weeks

	n.286<br>PT	NRT	MD	MD
		Printed materials	MD	MD
		Telephone counseling	4 counseling at baseline, 1, 4 and 8 week	8 weeks
	n.67<br>PT	Pharmacological therapy for highly addicted smokers	MD	MD
		NRT for highly addicted smokers	MD	MD
		Interactive tailored website resources	MD	MD
		Telephone/e-mail counseling by a nurse	MD	>1 month
	n.78<br>PT	Pharmacological therapy for highly addicted smokers	MD	MD
		NRT for highly addicted smokers	MD	MD
		Telephone/e-mail counseling by a nurse	Up to 5 sessions	MD
	n.163<br>PT	In-person counseling	15 minutes at 3, 6, 12 months	MD
		NRT	MD	Up to 10 weeks
		Pharmacological therapy (bupropion) if failed using NRT	MD	MD
		Computerized tailored feedback to motivate smokers' cessation and based on the stage of smoking changes	MD	MD
	n.49<br>PT	In-person counseling	15-30 minutes	MD
		NRT	MD	10 weeks
		Computer-delivered tailored printed materials	at 3 and 6 months	MD
Brown J.,<br>2013<br><br>[35]	n.48<br>Not PT	In-person counseling	1 counseling of 15 minutes	MD
		Printed materials	MD	MD
		Email	20 emails	MD
Brown N.,<br>2017<br><br>[80]	n.256<br>PT	NRT (if needed)<br>Telephone counseling	7 counseling	>3 months
	n.154<br>PT	In-person counseling at home	2 counseling	MD
		NRT	MD	8 weeks
		Telephone counseling	6 counseling 5-10 minutes each	>4 months
	n.32<br>Not PT	In-person counseling for smokers and their children	5 counseling for smokers	>6 months
			6 counseling 25 minutes each for children	>6 months
		Printed materials	1 lecture	MD
		Mobile phone text-message	5/day for 4 weeks and 3/week for 16 weeks	20 weeks
Byambasuren O.,<br>2023<br><br>[36]	n.21<br>PT	NRT (patches)	MD	8 weeks
		Video counseling delivered via telephone	10-30 minutes for each counseling	8 weeks (concurrent)
	n.21<br>PT	NRT (patches)	MD	8 weeks
		Telephone counseling	10-30 minutes for each counseling	8 weeks (concurrent)
Byaruhanga J.,<br>2020<br><br>[37]	n.57<br>Not PT	In-person counseling	5 counseling at 2, 4, 8, 12 and 24 weeks	24 weeks
		Mobile phone app (CASC)	MD	MD
		Mobile carbon monoxide checker	MD	MD
	n.280	NRT	MD	MD

	PT	Printed materials	MD	MD
		Computer-based internet video counseling (Polycom PVX)	4 counseling at baseline, 1, 4 and 8 week	8 weeks
	n.286	NRT	MD	MD
	PT	Printed materials	MD	MD
		Telephone counseling	4 counseling at baseline, 1, 4 and 8 week	8 weeks
	n.21	NRT (patches)	MD	8 weeks
	PT	Video counseling delivered via telephone	10-30 minutes for each counseling	8 weeks (concurrent)
	n.21	NRT (patches)	MD	8 weeks
	PT	Telephone counseling	10-30 minutes for each counseling	8 weeks (concurrent)
Cartujano-Barrera F., 2022 [38]	n.141	In-person counseling	MD	MD
	Not PT	Telephone counseling	7 counseling after hospital discharge	MD
Chhabra D., 2023 [39]	n.50	In-person group counseling	90 minutes each	6 weeks
	Not PT	Mobile phone App (SmartQuit)	MD	MD
Crilly P., 2020 [40]	n.80	In-person counseling by a pharmacist	1 counseling of 2 minutes	MD
	Not PT	Internet-based photo-aging software (APRIL)	MD	12 months
da Silva Teixeira R., 2023 [41]	n.197	NRT	MD	MD
	PT	Website resources	MD	54 weeks
		Mobile phone text-message	3 text-message/day	MD
		IVR	Daily interaction	MD
		Quitline	24h/day	From the 15th day
		Email	Daily	First 6 weeks
	n.1159	Pharmacological therapy/NRT	MD	MD
	PT	Interactive internet-based resource	MD	MD
		Internet-based chat group (peer-to-peer web forum and an expert-moderated forum)	MD	MD
	n.1159	Pharmacological therapy/NRT	MD	MD
	PT	Interactive internet-based resource	MD	MD
		Internet-based chat group (peer-to-peer web forum)	MD	MD
		Physical exercise program	MD	MD
	n.280	NRT	MD	MD
	PT	Printed materials	MD	MD
		Computer-based internet video counseling (Polycom PVX)	4 counseling at baseline, 1, 4 and 8 week	8 weeks
	n.286	NRT	MD	MD

	PT	Printed materials<br>Telephone counseling	MD<br>4 counseling at baseline, 1, 4 and 8 week	MD<br>8 weeks
do Amaral L.M., 2020<br><br>[42]	n.54<br>Not PT	In-person counseling of 20 minutes during hospitalization<br>Telephone counseling post-discharge	MD<br>at 1-4-8-12-16-20-26 weeks post-discharge	>26 weeks post-discharge
	n.696<br>Not PT	In-person counseling<br>Telephone counseling	N/D<br>N/D	N/D
	n.50<br>Not PT	In-person group counseling<br>IVR post-discharge<br>Additional counseling as needed	MD<br>at 3-14-30 days<br>MD	N/D
	n.198<br>PT	Pharmacological therapy<br>NRT<br>IVR<br>Fax	MD<br>NRT twice<br>at 2-14-30-60-90 days<br>MD	for 30 days<br>for 90 days<br>MD<br>MD
	n.199<br>PT	Pharmacological therapy<br>NRT<br>Quitline	MD<br>MD<br>Patient's choice	MD<br>MD<br>MD
	n.317<br>PT	NRT (patches)<br>Telephone advice	MD<br>MD	MD<br>MD
	n.597<br>PT	In-person counseling during hospitalization<br>Pharmacological therapy<br>IVR	MD<br>MD<br>at 4, 14, 28 and 49 days	>49 days
	n.301<br>Not PT	In-person counseling during hospitalization<br>Quitline	MD<br>Patient's choice	MD<br>MD
	n.748<br>Not PT	In-person counseling during hospitalization<br>Website resource	MD<br>MD	MD<br>MD
	n.527<br>PT	NRT<br>Quitline	MD<br>MD	MD
	n.527<br>PT	NRT<br>Quitline<br>Fax	MD<br>MD<br>MD	MD<br>MD<br>MD
	n.681<br>PT	In-person counseling<br>Pharmacological therapy<br>NRT<br>IVR	Patient's choice<br>MD<br>twice<br>at 2-12-28-58-88 days	MD<br>>90 days<br>MD<br>88 days
	n.678<br>PT	Pharmacological therapy<br>Quitline	MD<br>Patient's choice	MD<br>MD
	n.300<br>PT	In-person counseling<br><br>Pharmacological therapy	3 sessions<br><br>MD	During hospitalization and after 4 weeks,<br>and after 1 week and 28 days

				MD
		Printed support	MD	12 months
		Telephone counseling	1-6-12 months	MD
		Quitline	MD	
	n.300	Pharmacological therapy (bupropion/varenicline) or NRT	MD	>28 days
	PT	Telephone counseling	at 1-6-12 months	12 months
Eghdami S.,	n.285	In-person counseling	5 counseling	12 weeks (concurrent)
2023	PT	Mobile app (CureApp smoking cessation)	MD	24 weeks (concurrent)
		NRT	MD	MD
[43]		Chatbot	MD	24 weeks (concurrent)
		Mobile carbon monoxide checker	MD	24 weeks (concurrent)
	n.287	In-person counseling	5 counseling	12 weeks (concurrent)
	PT	NRT	MD	MD
		Mobile app (CureApp smoking cessation)	MD	24 weeks (concurrent)
	n.42	In-person counseling	MD	MD
	PT	NRT	MD	MD
		Mobile app and in app-messages	1/day	MD
		Mobile carbon monoxide checker	MD	MD
Fang Y.E.,	n.285	In-person counseling	5 counseling	12 weeks (concurrent)
2023	PT	Mobile app (CureApp smoking cessation)	MD	24 weeks (concurrent)
		NRT	MD	MD
[44]		Chatbot	MD	24 weeks (concurrent)
		Mobile carbon monoxide checker	MD	24 weeks (concurrent)
	n.287	In-person counseling	5 counseling	12 weeks (concurrent)
	PT	NRT	MD	MD
		Mobile app (CureApp smoking cessation)	MD	24 weeks (concurrent)
	n.304	In-person counseling	MD	MD
	Not PT	Printed materials	MD	MD
		Mobile phone tailored text-messages	MD	MD
		Telephone counseling	MD	MD
	n.375	In-person counseling	MD	MD
	Not PT	Printed materials	MD	MD
		Mobile phone not tailored text-messages	MD	MD
		Telephone counseling	MD	MD
	n.188	NRT	MD	MD
	PT	Mobile phone text-message	MD	MD
		Telephone counseling	MD	MD
	n.213	NRT	MD	MD
	PT	Mobile phone text-message	MD	MD

Gainsbury S., 2011 [45]	n.11143 Not PT	Printed materials	MD	MD
		Website resources	MD	MD
		Telephone counseling	5 counseling	MD
		Tailored email	20 emails	MD
	n.48 Not PT	In-person counseling	1 counseling of 15 minutes	MD
		Printed materials	MD	MD
		Email	20 emails	MD
	n.101 PT	In-person group counseling	7 counseling	MD
		NRT (patches)	MD	MD
		Printed materials	MD	MD
		Email (newsletters and support emails)	64 newsletters	MD
Graham A.L., 2016 [71]	n.197 PT	NRT	MD	MD
		Website resources	MD	54 weeks
		Mobile phone text-message	3 text-message/day	MD
		IVR	Daily interaction	MD
		Quitline	24h/day	From the 15th day
		Email	Daily	First 6 weeks
	n.67 PT	Pharmacological therapy for highly addicted smokers	MD	MD
		NRT for highly addicted smokers	MD	MD
		Interactive tailored website resources	MD	MD
		Telephone/e-mail counseling by a nurse	MD	>1 month
	n.78 PT	Pharmacological therapy for highly addicted smokers	MD	MD
		NRT for highly addicted smokers	MD	MD
		Telephone/e-mail counseling by a nurse	Up to 5 sessions	MD
	n.230 PT	Pharmacological therapy (bupropion)/NRT	MD	MD
		Website tailored resources from an oncologist	Patient choice	MD
	n.402 PT	Pharmacological therapy (varenicline)	MD	12 weeks
		Printed materials	MD	MD
		Telephone counseling	Up to 5 counseling	MD
		Quitline	MD	MD
	n.401 PT	Pharmacological therapy (varenicline)	MD	12 weeks
		Printed materials	MD	MD
		Website resources	MD	MD
		Website chat group	MD	MD
		Quitline	MD	MD
	n.399 PT	Pharmacological therapy (varenicline)	MD	12 weeks
		Printed materials	MD	MD
		Website resources	MD	MD
		Website chat group	MD	MD

		Telephone counseling<br>Quitline	Up to 5 counseling<br>MD	MD<br>MD
	n.58<br>PT	NRT (smokers of 5 or more cigarettes)<br>Computer-based internet counseling	MD<br>5 counseling	Up to 10 weeks<br>Up to 12 months
	n.1159<br>PT	Pharmacological therapy/NRT<br>Interactive internet-based resource<br>Internet-based chat group (peer-to-peer web forum and an expert-moderated forum)	MD<br>MD<br>MD	MD<br>MD<br>MD
	n.1159<br>PT	Pharmacological therapy/NRT<br>Interactive internet-based resource<br>Internet-based chat group (peer-to-peer web forum)<br>Physical exercise program	MD<br>MD<br>MD<br>MD	MD<br>MD<br>MD<br>MD
	n.140<br>PT	In person counseling<br>Pharmacological therapy (bupropion)<br>Website resources (CHESS SCRP a guided universe of information, problem solving and emotional support)	3 brief counseling<br>150 mg<br>MD	MD<br>9 weeks<br>12 weeks
Han M.,<br>2018	n.42<br>Not PT	Printed materials on smoking cessation and healthy diet<br>Social media chat group (WhatsApp)	MD<br>3 reminders/week	MD<br>MD
[73]	n.40<br>Not PT	Printed materials on smoking cessation and healthy diet<br>Social media chat group (Facebook)	MD<br>3 reminders/week	MD<br>MD
Harrogate S.,<br>2023<br><br>[46]	n.111<br>PT	In-person counseling by a nurse<br><br>NRT (gums)<br>Printed materials<br>Telephone counseling<br>Quitline	15 minutes<br><br>MD<br>MD<br>1 counseling/1 week in the first month, 1 counseling/2 weeks in the second and third month, 9th and 16th weeks post-discharge	1-3 weeks before surgery and after discharge<br><br>MD<br>MD<br>16th post-discharge
	n.48<br>PT	In-person/telephone counseling<br><br>NRT<br><br>Quitline	1 counseling/week for 4 weeks pre-surgery and 4 weeks after surgery<br>MD<br><br>MD	8 weeks<br><br>8 weeks (4 pre-surgery and 4 post-surgery)<br>MD
	n.81<br>PT	In-person counseling (pre-surgery)<br>NRT<br>Printed materials<br>Quitline	<5 minutes<br>MD<br>MD<br>MD	MD<br>6 weeks<br>MD<br>MD
	n.151<br>PT	In-person counseling (pre-surgery)<br>Pharmacological therapy (varenicline)<br>Printed materials	10-15 minutes<br>MD<br>MD	MD<br>3 months<br>MD

		Telephone counseling	MD	MD
		Quitline	MD	MD
		Fax	MD	MD
	n.145<br>Not PT	In-person counseling	3-5 minutes	MD
		Quitline	MD	MD
	n.40<br>Not PT	In-person counseling	60 minutes	MD
		Telephone counseling	MD	1 week post discharge
		Video counseling	60 minutes	MD
	n.14<br>Not PT	In-person counseling (post-surgery)	3 counseling	MD
		Printed materials	MD	MD
		Telephone counseling	At discharge, 2 weeks, 1 and 2 month	2 months
	n.137<br>PT	In-person counseling	1 counseling of 30-60 minutes	MD
		NRT (if needed)	MD	3 months
		Printed materials	MD	MD
		Telephone counseling	5 counseling of <30 minutes 1/week	2 months
		Video resources	10 minutes	MD
	n.71<br>Not PT	In-person counseling	2 hours prior to discharge	At discharge
		Telephone counseling	1 counseling/month	1 year
Hawes M.R., 2021<br><br>[47]	n.104<br>PT	In-person counseling in the hospital	1 counseling of 10-15 minutes	At the baseline
		NRT	MD	14 weeks
		Printed materials	MD	4 months
		Telephone counseling	15 counseling	4 months (concomitant)
		Quitline	MD	4 months (concomitant)
	n.101<br>PT	NRT	MD	During hospitalization and 3 days post-discharge
		Quitline	MD	4 months
	n.113<br>PT	In-person counseling	2 counselling 90 minutes each	MD
		NRT (patches)	MD	10 weeks
		Computer-delivered tailored printed materials	MD	At 3 and 6 months
		Telephone counseling	16 counseling 10 minutes each	At 3 and 6 months
	n.379<br>PT	In-person counseling	1 counseling of 10-15 minutes	MD
		NRT	MD	3 months
		Printed materials	MD	MD
		Telephone counseling	At least 11 counseling of 15 minutes	4 months
		Quitline	MD	4 months (concomitant)
	n.375<br>PT	NRT	MD	During hospitalization and 3 days post-discharge
		Quitline	MD	4 months
Hutton H.E.,	n.140	In-person counseling	3 brief counseling	MD

2011<br><br>[72]	PT	Pharmacological therapy (bupropion)<br>Website resources (CHESS SCRP a guided universe of information, problem solving and emotional support)	150 mg<br>MD	9 weeks<br>12 weeks
	n.1159<br>PT	Pharmacological therapy/NRT<br>Interactive internet-based resource<br>Internet-based chat group (peer-to-peer web forum)<br>Physical exercise program	MD<br>MD<br>MD<br>MD	MD<br>MD<br>MD<br>MD
	n.197<br>PT	NRT<br>Website resources<br>Mobile phone text-message<br>IVR<br>Quitline<br>Email	MD<br>MD<br>3 text-message/day<br>Daily interaction<br>24h/day<br>Daily	MD<br>54 weeks<br>MD<br>MD<br>From the 15th day<br>First 6 weeks
	n.1159<br>PT	Pharmacological therapy/NRT<br>Interactive internet-based resource<br>Internet-based chat group (peer-to-peer web forum and an expert-moderated forum)	MD<br>MD<br>MD	MD<br>MD<br>MD
Iaccarino J.M., 2019<br><br>[48]	n.27<br>Not PT	Not-tailored printed materials<br>Quitline	MD<br>MD	MD<br>MD
	n.28<br>Not PT	In-person counseling<br>Take home audio materials<br>Printed materials<br>Quitline	1 counseling<br>MD<br>MD<br>MD	MD<br>MD<br>MD<br>MD
Kant R., 2021<br><br>[74]	n.80<br>Not PT	In-person counseling by a pharmacist<br>Internet-based photo-aging software (APRIL)	1 counseling of 2 minutes<br>MD	MD<br>12 months
	n.140<br>PT	In person counseling<br>Pharmacological therapy (bupropion)<br>Website resources (CHESS SCRP a guided universe of information, problem solving and emotional support)	3 brief counseling<br>150 mg<br>MD	MD<br>9 weeks<br>12 weeks
	n.197<br>PT	NRT<br>Website resources<br>Mobile phone text-message<br>IVR<br>Quitline<br>Email	MD<br>MD<br>3 text-message/day<br>Daily interaction<br>24h/day<br>Daily	MD<br>54 weeks<br>MD<br>MD<br>From the 15th day<br>First 6 weeks
Krishnan N., 2021	n.114<br>Not PT	In-person counseling<br>Printed materials	MD<br>MD	MD<br>MD

		Mobile phone text-message	9500 text-messages	MD
[49]	n.44<br>PT	In-person counseling<br>Pharmacological therapy (bupropion/varenicline)<br>or NRT<br>Printed materials<br>Mobile phone not-tailored text-message (WhatsApp)	MD<br>MD<br>MD<br>MD<br>MD	3 months
	n.114<br>Not PT	In-person counseling<br>Printed materials<br>Mobile phone text-message	MD<br>MD<br>9500 text-messages	MD<br>MD<br>MD
Li S.,<br>2024<br><br>[50]	n.188<br>PT	NRT<br>Mobile phone text-message<br>Telephone counseling	MD<br>MD<br>MD	MD<br>MD<br>MD
	n.80<br>Not PT	In-person counseling by a pharmacist<br>Internet-based photo-aging software (APRIL)	1 counseling of 2 minutes<br>MD	MD<br>12 months
	n.42<br>Not PT	Printed materials on smoking cessation and healthy diet<br>Social media chat group (WhatsApp)	MD<br>3 reminders/week	MD<br>MD
	n.40<br>Not PT	Printed materials on smoking cessation and healthy diet<br>Social media chat group (Facebook)	MD<br>3 reminders/week	MD<br>MD
	n.58<br>PT	NRT (smokers of 5 or more cigarettes)<br>Computer-based internet counseling	MD<br>5 counseling	Up to 10 weeks<br>Up to 12 months
	n.197<br>PT	NRT<br>Website resources<br>Mobile phone text-message<br>IVR<br>Quitline<br>Email	MD<br>MD<br>3 text-message/day<br>Daily interaction<br>24h/day<br>Daily	NRT<br>54 weeks<br>MD<br>MD<br>From the 15th day<br>First 6 weeks
	n.49<br>PT	Pharmacological therapy (bupropion or varenicline)<br>Mobile phone App	MD<br>MD	MD<br>MD
Liu S.,<br>2017<br><br>[51]	n.197<br>PT	NRT<br>Website resources<br>Mobile phone text-message<br>IVR<br>Quitline<br>Email	MD<br>MD<br>3 text-message/day<br>Daily interaction<br>24h/day<br>Daily	MD<br>54 weeks<br>MD<br>MD<br>From the 15th day<br>First 6 weeks
Mann-Jackson L.,<br>2019<br><br>[52]	n.247<br>PT	Pharmacological therapy (varenicline) or NRT<br>Telephone counseling by a nurse	MD<br>1 session/week	MD<br>Over 12 weeks

Matkin W., 2019 [75]	n.463 Not PT	Tailored printed material<br>Telephone counseling<br>Quitline	MD<br>2 counseling of 10-15 minutes each<br>MD	MD<br>At 4, 8, 16, and 20 weeks<br>MD
	n.245 PT	NRT (patches)<br>Printed materials<br>Telephone counseling	MD<br>MD<br>6 counseling/week of 30 minutes for each session	MD<br>MD<br>12 weeks
	n.338 Not PT	Printed materials (8 pages)<br>Telephone counseling by a nurse	MD<br>4 counseling of 5 minutes each	MD<br>Within 7 days of enrolment, at 2, 6 and 12 months
	n.1690 PT	In-person counseling<br>NRT (patches)<br>Printed materials by the U.S. Public Health Service<br>Quitline	4 counseling of 3-10 minutes each<br>MD<br>MD<br>MD	12 months<br>MD<br>12 months (concomitant)<br>12 months (concomitant)
	n.209 Not PT	Printed materials<br>Telephone counseling	MD<br>4 counseling	MD<br>4-6 weeks
	n.85 Not PT	Printed materials (Clear Horizons)<br>Quitline<br>Email	MD<br>MD<br>MD	MD<br>MD<br>MD
	n.92 Not PT	Printed materials (Clear Horizons)<br>Telephone counseling<br>Quitline	MD<br>2 counseling<br>MD	MD<br>At 4 and 8 weeks<br>MD
	n.1124 Not PT	Printed materials<br>Telephone counseling	MD<br>1 counseling of 30-40 minutes<br>Up 5 counseling of 10-15 minutes each	MD<br>Pre-quit date<br>At baseline, 3, 7, 14, and 30 days after the quit date
	n.873 Not PT	Printed materials (ALA Freedom From Smoking in 20 Days)<br>Television series<br>Quitline<br>Email	MD<br>MD<br>At patient's choice<br>>10	MD<br>MD<br>MD<br>6 months
	n.438 Not PT	Printed materials (ALA Freedom From Smoking in 20 Days)<br>Television series	MD<br>MD	MD<br>MD
McCrabb S., 2019 [53]	n.197 PT	NRT<br>Website resources<br>Mobile phone text-message<br>IVR<br>Quitline<br>Email	MD<br>MD<br>3 text-message/day<br>Daily interaction<br>24h/day<br>Daily	MD<br>54 weeks<br>MD<br>MD<br>From the 15th day<br>First 6 weeks

	n.80<br>Not PT	In-person counseling by a pharmacist<br>Internet-based photo-aging software (APRIL)	1 counseling of 2 minutes<br>MD	MD<br>12 months
	n.203<br>PT	NRT<br>Website tailored resources<br>Website-based counseling	MD<br>MD<br>MD	MD<br>MD<br>MD
	n.205<br>PT	NRT/pharmacological therapy<br>In-person group counseling by a doctoral-level psychologist<br>Telephone counseling by a doctoral-level psychologist	MD<br>MD<br>MD	MD<br>MD<br>MD
	n.67<br>PT	Pharmacological therapy for highly addicted smokers<br>NRT for highly addicted smokers<br>Interactive tailored website resources<br>Telephone/e-mail counseling by a nurse	MD<br>MD<br>MD<br>MD	MD<br>MD<br>MD<br>>1 month
	n.78<br>PT	Pharmacological therapy for highly addicted smokers<br>NRT for highly addicted smokers<br>Telephone/e-mail counseling by a nurse	MD<br>MD<br>Up to 5 sessions	MD<br>MD<br>MD
	n.230<br>PT	Pharmacological therapy (bupropion)/NRT<br>Website tailored resources from an oncologist	MD<br>Patient choice	MD<br>6 months
	n.748<br>Not PT	In-person counseling during hospitalization<br>Website resource	MD<br>MD	MD<br>MD
	n.58<br>PT	NRT (smokers of 5 or more cigarettes)<br>Computer-based internet counseling	MD<br>5 counseling	Up to 10 weeks<br>Up to 12 months
	n.140<br>PT	In person counseling<br>Pharmacological therapy (bupropion)<br>Website resources (CHESS SCRP a guided universe of information, problem solving and emotional support)	3 brief counseling<br>150 mg<br>MD	MD<br>9 weeks<br>12 weeks
	n.1159<br>PT	Pharmacological therapy/NRT<br>Interactive internet-based resource<br>Internet-based chat group (peer-to-peer web forum and an expert-moderated forum)	MD<br>MD<br>MD	MD<br>MD<br>MD
	n.1159<br>PT	Pharmacological therapy/NRT<br>Interactive internet-based resource<br>Internet-based chat group (peer-to-peer web forum)<br>Physical exercise program	MD<br>MD<br>MD<br>MD	MD<br>MD<br>MD<br>MD
	n.552<br>Not PT	Tailored printed materials (4-5 page feedback letter)<br>Email (the same 4-5 page feedback letter)	At baseline and at 6 week<br>At baseline and at 6 week	6 weeks<br>6 weeks
	n.84<br>Not PT	Printed materials (of the website resources)<br>Creation of a group video message	MD<br>MD	MD<br>MD
Naslund J.A., 2017	n.42<br>Not PT	Printed materials on smoking cessation and healthy diet<br>Social media chat group (WhatsApp)	MD<br>3 reminders/week	MD<br>MD

	n.40	Printed materials on smoking cessation and healthy diet	MD	MD
[54]	Not PT	Social media chat group (Facebook)	3 reminders/week	MD
O'Logbon J., 2024	n.84	Printed materials (of the website resources)	MD	MD
	Not PT	Creation of a group video message	MD	MD
[55]				
Peckham E., 2017	n.113	In-person counseling	2 counselling 90 minutes each	MD
	PT	NRT (patches)	MD	10 weeks
		Computer-delivered tailored printed materials	MD	At 3 and 6 months
[76]		Telephone counseling	16 counseling 10 minutes each	At 3 and 6 months
Piñeiro B., 2016	n.187	Pharmacological therapy (varenicline)	MD	3 months
	PT	Telephone counseling	MD	MD
	n.9	In-person counseling by an oncologist before lung computer tomography	12 counseling	3 months
[56]	PT	Pharmacological therapy	MD	
		Telephone counseling by a nurse	1 counseling/week	
	n.9	In-person counseling by an oncologist after lung computer tomography	12 counseling	3 months
	PT	Pharmacological therapy	MD	
		Telephone counseling by a nurse	1 counseling/week	
Ricker A.B., 2024	n.89	In-person counseling by a surgeon and oncologist nurse	MD	MD
	PT	NRT	MD	MD
		Printed materials	MD	MD
[57]		Telephone counseling	MD	MD
	n.95	In-person counseling by a surgeon and oncologist nurse	MD	MD
	PT	NRT	MD	MD
		Printed materials	MD	MD
		Computer-based resource ("QuitPal")	MD	MD
		Telephone counseling	MD	MD
	n.134	In-person counseling during hospitalization	MD	MD
	PT	Pharmacological therapy (varenicline)	MD	MD
		Telephone counseling	MD	MD
	n.119	In-person counseling during hospitalization	MD	MD
	Not PT	Telephone counseling	MD	MD
Saroj S.K., 2022	n.1124	Printed materials	MD	MD
	Not PT	Telephone counseling	1 counseling of 30-40 minutes	Pre-quit date
			Up 5 counseling of 10-15 minutes each	At baseline, 3, 7, 14, and 30 days after the quit date
[58]				
Sawyer C.,	n.21	In-person counseling	MD	MD

2023	PT	Pharmacological therapy (bupropion) or NRT	1/day for 1 week, 2/day for 6 months	2 weeks before the quit date, for 6 months
[59]		Mobile phone App (iCOMMIT)	MD	MD
		Telephone counseling	5 counseling	MD
	n.13	In-person counseling	MD	MD
	PT	Pharmacological therapy (bupropion) or NRT	1/day for 1 week, 2/day for 6 months	2 weeks before the quit date, for 6 months
		Telephone counseling	5 counseling	MD
Setchoduk K., 2023 [60]	n.133 Not PT	In-person counseling	MD	MD
		Website materials	MD	MD
Shahab L., 2009 [61]	n.197 PT	NRT	MD	MD
		Website resources	MD	54 weeks
		Mobile phone text-message	3 text-message/day	MD
		IVR	Daily interaction	MD
		Quitline	24h/day	From the 15th day
		Email	Daily	First 6 weeks
Spanakis P., 2022 [62]	n.113 PT	In-person counseling	2 counselling 90 minutes each	MD
		NRT (patches)	MD	10 weeks
		Computer-delivered tailored printed materials	MD	At 3 and 6 months
		Telephone counseling	16 counseling 10 minutes each	At 3 and 6 months
Stead L.F., 2013 [78]	n.279 PT	NRT (gum)	MD	MD
		Printed materials	MD	MD
		Telephone counseling	MD	MD
	n.1425 Not PT	In-person counseling	1 counseling of 10 minutes	MD
		Printed materials	MD	MD
		Emails	MD	MD
Stead L.F., 2017 [77]	n.281 Not PT	Printed materials (ALA Freedom from Smoking in 20 days)	MD	MD
		Television series on smoking cessation	MD	MD
	n.283 Not PT	In-person group counseling	6 counseling<br>14 counseling	3 weeks<br>6 months
		Printed materials (ALA Lifetime of Freedom from Smoking)	MD	MD
		Television series on smoking cessation	MD	MD
	n.380 Not PT	In-person group counseling	4 counseling of 90 minutes each	MD
		Printed materials (ALA Freedom from Smoking in 20 days, Quitters Guide, and Buddy Guide)	MD	MD
		Telephone counseling	2 counseling	At 1 and 2 months
		Television series on smoking cessation	MD	MD

	n.109<br>Not PT	Printed materials (ALA Freedom from Smoking in 20 days, Quitters Guide, and Buddy Guide)	MD	MD
		Television series on smoking cessation	MD	MD
	n.675<br>Not PT	In-person group counseling	9 counseling	2 months
		Video resources	MD	MD
	n.675<br>Not PT	Printed materials	MD	MD
		Video resources	10 minutes	MD
		Telephone counseling	1 counseling	MD
		Emails	MD	MD
Tatnell P., 2022<br><br>[63]	n.50<br>Not PT	In-person group (5-6 subjects) counseling	60 minutes each	6 weeks
		Virtual reality eyewear for cue exposure in activities related to smoking	5 session	MD
Taylor G.M.J., 2017<br><br>[64]	n.80<br>Not PT	In-person counseling by a pharmacist	1 counseling of 2 minutes	MD
		Internet-based photo-aging software (APRIL)	MD	12 months
	n.58<br>Not PT	NRT (smokers of 5 or more cigarettes)	MD	Up to 10 weeks
		Computer-based internet counseling	5 counseling	Up to 12 months
	n.197<br>PT	NRT	MD	NRT
		Website resources	MD	54 weeks
		Mobile phone text-message	3 text-message/day	MD
		IVR	Daily interaction	MD
		Quitline	24h/day	From the 15th day
		Email	Daily	First 6 weeks
	n.203<br>PT	NRT	MD	MD
		Website tailored resources	MD	MD
		Website-based counseling	MD	MD
	n.205<br>PT	NRT/pharmacological therapy	MD	MD
		In-person group counseling by a doctoral-level psychologist	MD	MD
		Telephone counseling by a doctoral-level psychologist	MD	MD
	n.67<br>PT	Pharmacological therapy for highly addicted smokers	MD	MD
		NRT for highly addicted smokers	MD	MD
		Interactive tailored website resources	MD	MD
		Telephone/e-mail counseling by a nurse	MD	>1 month
	n.78<br>PT	Pharmacological therapy for highly addicted smokers	MD	MD
		NRT for highly addicted smokers	MD	MD
		Telephone/e-mail counseling by a nurse	Up to 5 sessions	MD
	n.230<br>PT	Pharmacological therapy (bupropion)/NRT	MD	MD
		Website tailored resources from an oncologist	Patient choice	MD
	n.42	Pharmacological therapy (varenicline)	MD	MD

	PT	NRT	MD	MD
		Social media chat group	MD	MD
	n.1159	Pharmacological therapy/NRT	MD	MD
	PT	Interactive internet-based resource	MD	MD
		Internet-based chat group (peer-to-peer web forum and an expert-moderated forum)	MD	MD
	n.1159	Pharmacological therapy/NRT	MD	MD
	PT	Interactive internet-based resource	MD	MD
		Internet-based chat group (peer-to-peer web forum)	MD	MD
		Physical exercise program	MD	MD
	n.402	Pharmacological therapy (varenicline)	MD	12 weeks
	PT	Printed materials	MD	MD
		Telephone counseling	Up to 5 counseling	MD
		Quitline	MD	MD
	n.401	Pharmacological therapy (varenicline)	MD	12 weeks
	PT	Printed materials	MD	MD
		Website resources	MD	MD
		Website chat group	MD	MD
		Quitline	MD	MD
	n.399	Pharmacological therapy (varenicline)	MD	12 weeks
	PT	Printed materials	MD	MD
		Website resources	MD	MD
		Website chat group	MD	MD
		Telephone counseling	Up to 5 counseling	MD
		Quitline	MD	MD
	n.132	In-person counseling by a nurse	At 6 weeks	MD
	Not PT	Computer-based tailored and interactive message	MD	MD
		Telephone counseling by a nurse	At 6 months	MD
	n.84	Printed materials (of the website resources)	MD	MD
	Not PT	Creation of a group video message	MD	MD
	n.748	In-person counseling during hospitalization	MD	MD
	Not PT	Website resource	MD	MD
	n.140	In person counseling	3 brief counseling	MD
	PT	Pharmacological therapy (bupropion)	150 mg	9 weeks
		Website resources (CHESS SCRP a guided universe of information, problem solving and emotional support)	MD	12 weeks
Thomsen T., 2014	n.48<br>PT	In-person/telephone counseling	1 counseling/week for 4 weeks pre-surgery and 4 weeks after surgery	8 weeks
		NRT	MD	

[65]		Quitline	MD	8 weeks (4 pre-surgery and 4 post-surgery)<br>MD
	n.111<br>PT	In-person counseling by a nurse<br><br>NRT (gums)<br>Printed materials<br>Telephone counseling<br>Quitline	15 minutes<br><br>MD<br>MD<br>1 counseling/1 week in the first month, 1 counseling/2 weeks in the second and third month, 9th and 16th weeks post-discharge	1-3 weeks before surgery and after discharge<br><br>MD<br>MD<br>16th post-discharge
Tzelepis F., 2019<br><br>[79]	n.21<br>PT	NRT (patches)<br>Video counseling delivered via telephone	MD<br>10-30 minutes for each counseling	8 weeks<br>8 weeks (concurrent)
	n.21<br>PT	NRT (patches)<br>Telephone counseling	MD<br>10-30 minutes for each counseling	8 weeks<br>8 weeks (concurrent)
	n.280<br>PT	NRT<br>Printed materials<br>Computer-based internet video counseling (Polycom PVX)	MD<br>MD<br>4 counseling at baseline, 1, 4 and 8 week	MD<br>MD<br>8 weeks
	n.286<br>PT	NRT<br>Printed materials<br>Telephone counseling	MD<br>MD<br>4 counseling at baseline, 1, 4 and 8 week	MD<br>MD<br>8 weeks
Villanti A.C., 2020<br><br>[66]	n.615<br>PT	NRT<br>Internet-based multiple contests<br>Telephone counseling	MD<br>MD<br>6 counseling	2 weeks<br>4 months<br>MD
	n.602<br>PT	NRT<br>Internet-based single contest<br>Telephone counseling	MD<br>MD<br>6 counseling	2 weeks<br>4 months<br>MD
	n.615<br>PT	NRT<br>Internet-based multiple contest	MD<br>MD	2 weeks<br>4 months
	n.602<br>PT	NRT<br>Internet-based single contest	MD<br>MD	2 weeks<br>4 months
	n.48<br>Not PT	In-person counseling<br>Printed materials<br>Email	1 counseling of 15 minutes<br>MD<br>20 emails	MD<br>MD<br>MD
	n.204<br>Not PT	Printed materials<br>Telephone counseling	MD<br>MD	MD<br>MD
	n.209<br>Not PT	Printed materials<br>Telephone counseling	MD<br>4 counseling	MD<br>4-6 weeks
	n.84<br>Not PT	Printed materials (of the website resources)<br>Creation of a group video message	MD<br>MD	MD<br>MD

Whittaker R., 2016 [68]	n.30 Not PT	In-person counseling	1 counseling of 30 minutes	MD
		Mobile phone tailored text-message (TXT-2-Quit program)	1-4 text-message/day	2 months
	n.142 Not PT	Printed material	MD	MD
		Mobile phone text-message	4-5 text message/day	MD
	n.236 PT	NRT (if needed, patches)	MD	MD
		Printed material	MD	MD
		Telephone counseling	11 counseling	>3 months
		Quitline	MD	MD
	n.238 PT	NRT (if needed, patches)	MD	MD
		Printed material	MD	MD
		Computer-assisted audio self-interview	MD	MD
	n.54 PT	Pharmacological therapy (varenicline)	MD	12 weeks
		Printed material (a sheet tailored to HIV-positive subjects)	MD	MD
		Mobile phone text-message	2 text-messages/day (1 as the remainder of the medication and 1 motivational to quit)	12 weeks (concurrent)
		Quitline	MD	MD
	n.53 PT	Pharmacological therapy (varenicline)	MD	12 weeks
		Printed material (a sheet tailored to HIV-positive subjects)	MD	MD
		Mobile phone text-message as the remainder of the medication	MD	MD
		Quitline	MD	MD
Whittaker R., 2019 [67]	n.54 PT	Pharmacological therapy (varenicline)	MD	12 weeks
		Printed material (a sheet tailored to HIV-positive subjects)	MD	MD
		Mobile phone text-message	2 text-messages/day (1 as the remainder of the medication and 1 motivational to quit)	12 weeks (concurrent)
		Quitline	MD	MD
	n.338 Not PT	Printed materials (8 pages)	MD	MD
		Telephone counseling by a nurse	4 counseling of 5 minutes each	Within 7 days of enrolment, at 2, 6 and 12 months
	n.335 Not PT	Printed materials (8 pages)	MD	MD
		Mobile phone text-message	8 text-messages	Within 7 days of enrolment
	n.142 Not PT	Printed material	MD	MD
		Mobile phone text-message	4-5 text message/day	MD
	n.53 PT	Pharmacological therapy (varenicline)	MD	12 weeks
		Printed material (a sheet tailored to HIV-positive subjects)	MD	MD
		Mobile phone text-message as the remainder of the medication	MD	MD
		Quitline	MD	MD
	n.156 PT	NRT	MD	MD
		Mobile phone app	MD	MD
		Telephone counseling	MD	MD

		Mobile carbon monoxide checker	MD	MD
	n.154<br>PT	NRT<br>Telephone counseling	MD	MD
	n.90<br>PT	NRT (patches)	21 mg for 4 weeks, 14 mg for 2 weeks, 7 mg for 2 weeks	8 weeks
		Telephone counseling	2 counseling/week of 10 minutes each	4 weeks
		IVR	3 prompts/day	4 weeks
		Video clips to record the carbon monoxide test	3 prompts/day	4 weeks
		Mobile carbon monoxide checker	3 prompts/day	4 weeks
	n.114<br>Not PT	In-person counseling	MD	MD
		Printed materials	MD	MD
		Mobile phone text-message	9500 text-messages	MD
	n.30<br>Not PT	In-person counseling	1 counseling of 30 minutes	MD
		Mobile phone tailored text-message (TXT-2-Quit program)	1-4 text-message/day	2 months
Williams P.J., 2023<br><br>[69]	n.9<br>PT	In-person counseling by an oncologist before lung computer tomography	12 counseling	3 months
		Pharmacological therapy	MD	
		Telephone counseling by a nurse	1 counseling/week	
	n.9<br>PT	In-person counseling by an oncologist after lung computer tomography	12 counseling	3 months
		Pharmacological therapy	MD	
		Telephone counseling by a nurse	1 counseling/week	
	n.27<br>Not PT	Not-tailored printed materials	MD	MD
		Quitline	MD	MD
	n.28<br>Not PT	In-person counseling	1 counseling	MD
		Take home audio materials	MD	MD
		Printed materials	MD	MD
		Quitline	MD	MD
Zbikowski S.M., 2012<br><br>[70]	n.22<br>PT	In-person counseling	12 counseling of 90 minutes each	3 months
		NRT (gums)	MD	3 months (concomitant) and others 4 months (if needed)
		Printed materials	MD	3 months (concomitant)
		Telephone counseling	MD	3 months (concomitant)
	n.22<br>Not PT	In-person counseling	12 counseling of 90 minutes each	3 months
		Printed materials	MD	3 months (concomitant)
		Telephone counseling	MD	3 months (concomitant)
	n.18<br>Not PT	In-person counseling	12 counseling of 90 minutes each	3 months
		Printed materials	MD	3 months (concomitant)
		Telephone counseling	MD	3 months (concomitant)

		Physical exercise (graduated walking and exercise)	MD	3 months (concomitant)
	n.1690	In-person counseling	4 counseling of 3-10 minutes each	12 months
	PT	NRT (patches)	MD	MD
		Printed materials by the U.S. Public Health Service	MD	12 months (concomitant)
		Quitline	MD	12 months (concomitant)
	n.85	Printed materials (Clear Horizons)	MD	MD
	Not PT	Quitline	MD	MD
		Email	MD	MD
	n.92	Printed materials (Clear Horizons)	MD	MD
	Not PT	Telephone counseling	2 counseling	At 4 and 6 weeks
		Quitline	MD	MD
	n.279	NRT (gum)	MD	MD
	PT	Printed materials	MD	MD
		Telephone counseling	MD	MD
	n.505	Printed materials (Clear Horizons)	MD	MD
	PT	Telephone counseling	2 counseling	MD
