# Supplementary material for: Effectiveness and Adherence of Pharmacological vs. Non-Pharmacological Technology-Supported Smoking Cessation Interventions: An Umbrella Review
Source: Healthcare (Basel). 2025 Apr 21;13(8):953. doi: 10.3390/healthcare13080953 (PMC12027406; doi:10.3390/healthcare13080953)
Supplement: Supplementary file 1 [file healthcare-13-00953-s001.zip › Supplementary File S3 - Pharmacological and Non-Pharmacological Technology-supported Smoking Cessation Interventions.pdf]

Supplementary File S3: Pharmacological and Non-Pharmacological Technology-supported Smoking Cessation Interventions

3.3 Pharmacological Therapy Smoking Cessation Interventions

3.3.1. In-person counseling plus Pharmacological therapy plus Video counseling

The smoking cessation strategy based on in-person counseling plus pharmacological therapy plus video counseling was reported by three studies [36,37,79].

It was conducted on 63 smokers, all of whom had HIV, and were female (0 male to 63 female), with a range age of 18-75 years old, smoking more than five cigarettes per day for at least 6 months [36,37,79].

The smoking cessation programs had a duration of 8 weeks [36,37,79].

The 5.5 months CARs verified by cotinine test at 6 months amounted to 21 (33.33%) former smokers [36,37,79].

The 7 days PPA verified by cotinine test at 6 months amounted to 24 (38.10%) former smokers [36,37,79].

The adherence rate was 66.67% at 6 months (42 smokers completed all counseling session sessions at 6 months follow-up) [36,37,79].

The satisfaction rate was assessed using the Client Satisfaction Questionnaire, obtaining a mean score of 29.6 [36,37,79].

No other outcomes are available for this type of smoking cessation program.

3.3.2. In-person counseling plus Pharmacological therapy plus IVR

The smoking cessation strategy based on in-person counseling plus pharmacologicaltherapy plus Interactive Voice Response (IVR) was reported by one study [42].

It was conducted on 1278 smokers, all of them were hospitalized patients for not defined diseases [42].

The smoking cessation programs had a duration major of 49 days for 597 smokers and a major of 88 days for the other 681 smokers [42].

The 6 months CARs self-reported at 6 months were recorded in 681 smokers and amounted to 211 (31%) former smokers [42].

The 7 days PPA verified by CO and cotinine test at 6 months was recorded in 681 smokers and amounted to 116 (17%) former smokers [42].

The 30 days PPA self-reported at 6 months was recorded in 597 smokers and amounted to 143 (24%) former smokers [42].

No other outcomes are available for this type of smoking cessation program.

3.3.3. In-person counseling plus Pharmacological therapy plus Computer-based materials

The smoking cessation strategy based on in-person counseling plus pharmacological therapy plus computer-based materials was reported by one study [34].

It was conducted on 325 smokers, 163 of them with unipolar depression, and 162 hospitalized for not-defined psychiatric disorders; the mean age was reported only for 113 smokers and was 39.9 years old; the gender ratio was reported only for 113 smokers and was of 1.26 male to 1 female (63 male and 50 female) [34].

The 7 days PPA verified by CO test at 6 months amounted to 44 (13.54%) former smokers [34].

The 7 days PPA verified by CO test at 12 months amounted to 52 (16.00%) former smokers [34].

The 7 days PPA verified by CO test at 18 months was recorded in 276 smokers and amounted to 48 (17.39%) former smokers [34].

No other outcomes are available for this type of smoking cessation program.

3.3.4. In-person counseling plus Pharmacological therapy plus Website resources

The smoking cessation strategy based on in-person counseling plus pharmacological therapy plus website resources was reported by five studies [53,64,71,72,74].

It was conducted on 700 smokers with a mean age of 40.68 years old and a gender ratio of 1 male to 1.22 female (315 male and 385 female) [53,64,71,72,74], who smoked at least ten cigarettes per day and the mean of cigarettes smoked per day was 21.1 [53,64,71,72,74].

The smoking cessation program had a duration of 3 months [53,64,71,72,74].

The 7 days PPA verified by CO test at 6 months was 105 (15.00%) former smokers [53,64,71,72,74].

The adherence rate was 85.00% at 6 months (the characteristics of smoking behaviour of 595 subjects were assessed at 6 months follow-up) [53,64,71,72,74].

No other outcomes are available for this type of smoking cessation program.

3.3.5. In-person counseling plus Pharmacological therapy plus Telephone counseling

The smoking cessation strategy based on in-person counseling plus pharmacolog-ical therapy plus telephone counseling was reported by thirteen studies [31,36,37,53,56,57,59,62,64,69,76,79,80].

It was conducted on 1253 smokers [31,36,37,53,56,57,59,62,64,69,76,79,80], 134 of them were hospitalized patients for pre-surgery not cardiac intervention [57], 63 were affected by HIV [36,37,79], 11 by schizoaffective disorders [59], and 2 by schizophrenia [59]; the mean age was recorded for 36 subjects and was 56.4 years old [56,69]; the range age was reported for 63 subjects between 18-75 years old, which were all female (gender ratio 0 male to 63 female) [36,37,79], and for 13 subjects were between 18-70 years old [59].

The characteristics of smoking behaviour before the intervention were recorded for 226 subjects who smoked more than fifteen cigarettes per day [62,76], 147 more than ten cigarettes per day for at least one year [57,59], 134 more than five per day for at least one year [31], 63 more than five per day for at least 6 months [36,37,79], 105 more than ten packs per year [31], and 32 more than twenty packs per year [56,69]. The FTND of 105 smokers was registered as higher than 4 [31], while the mean FTND was assessed for 239 smokers and was of 6.79 [59,62,76]. The smoking cessation status after the intervention was evaluated in 226 smokers and the number of smoked cigarettes per day was reduced by more than 50% at 12 and 18 months, 16% at 24 months, 24% at 30 months, and 23% at 36 months [62,76].

The smoking cessation had a duration of higher than 7 weeks for 134 smokers [31], 2 months for 294 [31,36,37,79], 3 months for 36 [56,69], 4 months for 154 [80], and 6 months for 105 [31], while for the other 530, the duration was not specified.

The 5.5 months CARs verified by cotinine test at 6 months were recorded in 63 smokers and amounted to 3 (4.76%) former smokers [36,37,79].

The 6 months CARs verified by CO test at 6 months were recorded in 105 smokers and amounted to 38 (36.19%) former smokers [31].

The 12 months CARs verified by cotinine test at 12 months were recorded in 134 smokers and amounted to 17 (12.69%) former smokers [31]

The 7 days PPA verified by CO test at 6 months was recorded in 190 smokers and amounted to 22 (11.58%) former smokers [56,69,80].

The 7 days PPA verified by cotinine test at 6 months was recorded in 76 smokers and amounted to 4 (5.26%) former smokers [36,37,59,79].

The 7 days PPA verified by CO and cotinine test at 6 months was recorded in 134 smokers and amounted to 48 (35.82%) former smokers [57].

The 30 days PPA verified by CO test at 6 months was recorded in 231 smokers and amounted to 143 (61.90%) former smokers [31].
The 7 days PPA self-reported at 6 months was recorded in 13 smokers and amounted to 2 (15.38%) former smokers [59].
The 30 days PPA self-reported at 6 months was recorded in 169 smokers and amounted to 20 (11.83%) former smokers [53,59,64].
The 7 days PPA self-reported at 12 months was recorded in 226 smokers and amounted to 14 (6.19%) former smokers [62,76].
The 7 days PPA self-reported at 18 months was recorded in 226 smokers and amounted to 18 (7.96%) former smokers [62,76].
The 7 days PPA self-reported at 24 months was recorded in 226 smokers and amounted to 18 (7.96%) former smokers [62,76].
The 7 days PPA self-reported at 30 months was recorded in 226 smokers and amounted to 18 (7.96%) former smokers [62,76].
The 7 days PPA self-reported at 36 months was recorded in 226 smokers and amounted to 18 (7.96%) former smokers [62,76].
The adherence rate was reported for 156 subjects and was 75.64% at 6 months (the characteristics of smoking behaviour of 116 subjects were assessed at 6 months follow-up) [53,64], while for 63 subjects was assessed as adherence to all counseling sessions and was 61.90% (39 subjects) [36,37,79].
The satisfaction rate was assessed in 63 subjects using the Client Satisfaction Questionnaire, obtaining a mean score of 28.9 [36,37,79].
The Beck Depression Inventory and the Brief Psychiatric Rating Scale were administered to 226 smokers and were significantly improved from baseline to 36 months, while the short Form survey on general functioning and the Global Assessment of Functioning were not significantly improved at any time points [62,76].
No other outcomes are available for this type of smoking cessation program.

3.3.6. In-person counseling plus Pharmacological therapy plus Mobile phone App
The smoking cessation strategy based on in-person counseling plus pharmacologicaltherapy plus mobile phone App was reported by four studies [32,33,43,44].
It was conducted on 1148 smokers with a mean age of 45 years old and a gender ratio of 2.73 male to 1 female (840 male and 308 female), smoking more than ten packs per year for at least ten years and with a TDS higher than five [32,33,43,44].
The smoking cessation program had a duration of 6 months [32,33,43,44].
The 4 months CARs verified by CO test at 6 months were 580 (50.52%) former smokers [32,33,43,44].
The 10 months CARs verified by CO test at 12 months were 476 (41.46%) former smokers [32,33,43,44].
No other outcomes are available for this type of smoking cessation program.

3.3.7. In-person counseling plus Pharmacological therapy plus Mobile phone App plus Mobile CO checker
The smoking cessation strategy based on in-person counseling plus pharmacologicaltherapy plus mobile phone App plus mobile CO checker was reported by one study [43].
It was conducted on 42 smokers, all of them with HIV [43].
The 6 months CARs verified by CO test at 6 months amounted to 6 (14.29%) former smokers [43].
No other outcomes are available for this type of smoking cessation program.

3.3.8. In-person counseling plus Pharmacological therapy plus Mobile phone App Chatbot plus Mobile CO checker

The smoking cessation strategy based on in-person counseling plus pharmacolog-ical therapy plus mobile phone App plus chatbot plus mobile CO checker was reported by four studies [32,33,43,44].

It was conducted on 1140 smokers with a mean age of 47 years old with a gender ratio of 3.13 male to 1 female (864 male and 276 female), smoking more than ten packs per year for at least ten years and with a TDS higher than five [32,33,43,44].

The smoking cessation program had a duration of 6 months [32,33,43,44].

The 4 months CARs verified by CO test at 6 months were 728 (63.86%) former smokers [32,33,43,44].

The 10 months CARs verified by CO test at 12 months were 596 (52.28%) former smokers [32,33,43,44].

No other outcomes are available for this type of smoking cessation program.

3.3.9. In-person counseling plus Pharmacological therapy plus Mobile phone App plus Telephone counseling

The smoking cessation strategy based on in-person counseling plus pharmacological therapy plus mobile phone App plus telephone counseling was reported by two studies [32,59].

It was conducted on 41 smokers with a mean age of 51.54 years old [32,59], 13 of them were affected by schizophrenia, 7 by schizoaffective disorders, and 1 by not-defined psychosis [59]; the gender ratio was reported only for 20 smokers and was of 9 male to 1 female (18 male and 2 female) [32]; all the 41 smokers smoked more than 10 cigarettes per day [32,59], and for 21 of them, a mean FTND of 5.8 [59].

The 7 days PPA self-reported at 6 months was recorded in 21 smokers and amounted to 4 (19%) former smokers [59].

The 7 days PPA verified by cotinine test at 6 months was recorded in 21 smokers and amounted to 3 (14.3%) former smokers [59].

The 7 days PPA verified by CO test at 6 months was recorded in 20 smokers and amounted to 6 (30.00%) former smokers [32].

The 30 days PPA self-reported at 6 months was recorded in 21 smokers and amounted to 1 (4.80%) former smoker [59].

No other outcomes are available for this type of smoking cessation program.

3.3.10. In-person counseling plus Pharmacological therapy plus Telephone counseling plus Quitline

The smoking cessation strategy based on in-person counseling plus pharmacolog-ical therapy plus telephone counseling plus quitline was reported by two studies [46,65].

It was conducted on 96 smokers all of them were hospitalized patients for pre-orthopedic or pre-general surgery [46,65].

The smoking cessation program had a duration of 8 weeks [46,65].

The PPA self-reported at 12 months amounted to 36 (37.50%) former smokers, but the time of verification was not specified [46,65].

After surgery, 2% of patients who stop to smoke pre-surgery had cardiovascular com-plications, while 0% recorded pulmonary complications [46,65].

No other outcomes are available for this type of smoking cessation program.

3.3.11. In-person counseling plus Pharmacological therapy plus Telephone counseling plus Computer-based materials

The smoking cessation strategy based on in-person counseling plus pharmacolog-ical therapy plus telephone counseling plus computer-based printed materials was re-ported by one study [47].

It was conducted on 113 smokers, 53 of them were affected by unipolar disorders, 29 by bipolar disorders, and 13 by schizophrenia, 18 by other not-defined mental health dis-orders, with a mean age of 39.9 years old, a gender ratio of 1.26 male to 1 female (63 male and 50 female), and a mean FTND of 4.8 [47].
The smoking cessation program had a duration of 6 months [47].
The 7 days PPA verified by CO test at 6 months amounted to 16 (14.4%) former smokers [47].
The 7 days PPA verified by CO test amounted at 12 months to 22 (19.4%) former smokers [47].
The 7 days PPA verified by CO test at 18 months amounted to 23 (20.35%) former smokers [47].
No other outcomes are available for this type of smoking cessation program.

3.3.12 In-person counseling plus Pharmacological therapy plus Printed materials plus Mobile phone Text-messages

The smoking cessation strategy based on in-person counseling plus printed mate-rials plus mobile phone text messages was reported by one study [49].
It was conducted on 44 smokers [49].
The smoking cessation program had a duration of 3 months [49].
The 6 months CARs self-reported at 6 months amounted to 18 (40.91%) former smokers [49].
No other outcomes are available for this type of smoking cessation program.

3.3.13. In-person counseling plus Pharmacological therapy plus Printed materials plus Email

The smoking cessation strategy based on in-person counseling plus printed materials plus email was reported by one study [45].
It was conducted on 101 smokers with a mean age of 45 years old and a gender ratio of 49.5 male to 1 female (99 male and 2 female) [45].
The 12 months CARs self-reported at 12 months amounted to 54 (53.47%) former smokers [45].
No other outcomes are available for this type of smoking cessation program.

3.3.14. In-person counseling plus Pharmacological therapy plus Printed materials plus Telephone counseling

The smoking cessation strategy based on in-person counseling plus pharmacolog-ical therapy plus telephone counseling was reported by two studies [57,70].
It was conducted on 111 smokers [57,70], 89 of them were cancer patients hospitalized for pre-resective surgery, smoking more than eight cigarettes per day [57].
The smoking cessation programs had a duration of 3 months for 22 smokers [70], while for the other 89, the duration was not specified [57].
The 7 days PPA verified by cotinine test at 6 months was recorded in 89 smokers and amounted to 28 (31.46%) former smokers [57].
The PPA at 7 months was recorded in 22 smokers and amounted to 11 (50.00%) former smokers, but time and methods of verification were not defined [70].
The PPA at 12 months was recorded in 22 smokers and amounted to 8 (36.36%) former smokers, but time and methods of verification were not defined [70].
No other outcomes are available for this type of smoking cessation program.

3.3.15. In-person counseling plus Pharmacological therapy plus Printed materials plus Quitline

The smoking cessation strategy based on in-person counseling plus pharmacolog-ical therapy plus printed materials plus quitline was reported by three studies [46,70,75].

It was conducted on 3461 smokers [46,70,75], 81 of them were hospitalized, pre-surgery patients [46]; the range age was reported for 3380 subjects and was higher than 65 years old [70,75].
The smoking cessation program duration of 12 months for 3380 smokers [70,75], while for the other 81, the duration was not specified.
The 7 days PPA self-reported at 6 months was recorded in 3380 smokers and amounted to 716 (21.18%) former smokers [70,75].
The 7 days PPA self-reported at 12 months was recorded in 3380 smokers and amounted to 652 (19.29%) former smokers [70,75].
The PPA at 12 months was recorded in 81 smokers and amounted to 17 (20.99%) former smokers, but time and methods of verification were not defined [46].
No other outcomes are available for this type of smoking cessation program.

3.3.16. In-person counseling plus Pharmacological therapy plus Printed materials plus Telephone counseling plus Quitline

The smoking cessation strategy based on in-person counseling plus pharmacolog-ical therapy plus printed materials plus telephone counseling plus quitline was reported by four studies [42,46,47,65].
It was conducted on 1005 smokers [42,46,47,65], 522 of them were hospitalized patients for not defined diseases [42,46,65], and in particular, 222 were hospitalized for pre-surgery [46,65], while the other 483 smokers had not defined psychiatric diseases [47]; the mean age was reported only for 104 smokers and was 38.1 years old [47]; the gender ratio was reported only for 104 smokers and was 1.17 male to 1 female (56 male and 48 female) [47].
The characteristics of smoking behaviour before the intervention were recorded for 104 subjects who smoked a mean of 22 cigarettes per day and with a mean FTND of 5.7 reduced to 10.3 cigarettes per day and 1.5 respectively after the program [47]. The characteristics of smoking behaviour after the programs were recorded in total for 483 subjects who smoked a mean of 7.28 cigarettes per day [47].
The smoking cessation programs had a duration of 4 months for 483 smokers [47], 12 months for 300 smokers [42], while for the other 222, the duration was not specified.
The 7 days PPA verified by CO test at 6 months was recorded in 483 smokers and amounted to 59 (12.21%) former smokers [47].
The 30 days PPA self-reported at 6 months was recorded in 300 smokers and amounted to 86 (28.8%) former smokers [42].
The 30 days PPA verified by CO test at 6 months was recorded in 300 smokers and amounted to 35 (11.6%) former smokers [42].
The 30 days PPA self-reported at 12 months was recorded in 300 smokers and amounted to 39 (13.0%) former smokers [42].
The 30 days PPA verified by CO test at 12 months was recorded in 300 smokers and amounted to 35 (11.6% former smokers [42].
The PPA verified by CO test or cotinine test at 12 months was recorded in 222 smokers and amounted to 44 (19.82%) former smokers, but the time of verification was not spec-ified [46,65].
The adherence rate was reported for 222 subjects and was 69.2% at 12 months (the characteristics of smoking behaviour of 154 subjects were assessed at 12 months follow-up) [46,65].
No other outcomes are available for this type of smoking cessation program.

3.3.17. In-person counseling plus Pharmacological therapy plus Printed materials plus Telephone counseling plus Quitline plus Fax

The smoking cessation strategy based on in-person counseling plus pharmacolog-ical therapy plus printed materials plus telephone counseling plus quitline plus fax was reported by one study [46].
It was conducted on 151 smokers, all of them were hospitalized for pre-surgery (not cardiac) [46].
The 7 days PPA at 12 months amounted to 64 (42.38%) former smokers, but the methods of verification were not defined [46].
No other outcomes are available for this type of smoking cessation program.

3.3.18. In-person counseling plus Pharmacological therapy plus Printed materials plus Telephone counseling plus Computer-based resource

The smoking cessation strategy based on in-person counseling plus pharmacolog-ical therapy plus printed materials plus telephone counseling plus computer-based re-sources was reported by one study [57].
It was conducted on 95 smokers, all of them were cancer patients hospitalized for pre-resective surgery, smoking more than 8 cigarettes per day [57].
The 7 days PPA verified by cotinine test at 6 months amounted to 30 (31.58%) former smokers [57].
No other outcomes are available for this type of smoking cessation program.

3.3.19. In-person counseling plus Pharmacological therapy plus Printed materials plus Telephone counseling plus Video counseling

The smoking cessation strategy based on in-person counseling plus pharmacolog-ical therapy plus printed materials plus telephone counseling plus video counseling was reported by one study [46].
It was conducted on 137 smokers, all of them were hospitalized pre-surgery patients [46].
The PPA at 12 months amounted to 23 (16.79%) former smokers, but time and methods of verification were not defined [46].
No other outcomes are available for this type of smoking cessation program.

3.3.20. Pharmacological therapy plus Chatbot

The smoking cessation strategy based on pharmacological therapy plus chatbot was reported by one study [33].
It was conducted on 242 smokers with a mean age of 49.01 years old with a gender ratio of 1 male to 1.53 female (96 male and 146 female), with a mean HSI of 2.71 [33].
The 6 months CARs verified by CO or cotinine test at 6 months were 63 (26.0%%) former smokers [33].
No other outcomes are available for this type of smoking cessation program.

3.3.21. Pharmacological therapy plus Computer-based Internet counseling

The smoking cessation strategy based on pharmacological therapy plus comput-er-based internet counseling was reported by five studies [34,50,53,64,71].
It was conducted on 290 smokers, all of them affected by HIV [34,50,53,64,71].
The characteristics of smoking behaviour after the intervention were a mean of 11.0 cigarettes per day at 6 months [34,50,53,64,71].
The smoking cessation programs had a duration longer than 12 months [34,50,53,64,71].
The 7 days PPA verified by CO test at 6 months amounted to 80 (27.58%) former smokers [34,50,53,64,71].
The 7 days PPA verified by CO test at 9 months amounted to 60 (20.69%) former smokers [34,50,53,64,71].
The 7 days PPA verified by CO test at 12 months amounted to 75 (25.86%) former smokers [34,50,53,64,71].
No other outcomes are available for this type of smoking cessation program.

3.3.22. Pharmacological therapy plus Mobile phone App

The smoking cessation strategy based on pharmacological therapy plus mobile phone App was reported by one study [50].

It was conducted on 49 smokers, with a mean FTND of 5.1 [50].

The 7 days PPA verified by CO or cotinine test at 6 months was 32 (65.31%) former smokers [50].

No other outcomes are available for this type of smoking cessation program.

3.3.23. Pharmacological therapy plus Social media Text-message

The smoking cessation strategy based on pharmacological therapy plus social media text-messages was reported by one study [64].

It was conducted on 42 smokers, all of them affected by chronic obstructive pulmonary disease, smoking more than five cigarettes per day for at least 2 years [64].

The 6 months CARs self-reported at 6 months amounted to 19 (45.24%) former smokers [64].

The mean ratio between the forced expiratory volume in one second (FEV1) and the forced vital capacity (FVC) registered at the end of the interventions was FEV1/FVC <70% [64].

No other outcomes are available for this type of smoking cessation program.

3.3.24. Pharmacological therapy plus Mobile phone Text-message

The smoking cessation strategy based on pharmacological therapy plus mobile phone text-messages was reported by two studies [42,44].

It was conducted on 530 smokers [42,44], 317 of them were hospitalized patients for not defined diseases [42].

The 30 days PPA self-reported at 6 months was recorded in 317 smokers and amounted to 72 (22.8%) former smokers [42].

The 30 days PPA verified by cotinine test at 6 months was recorded in 213 smokers and amounted to 19 (8.92%) former smokers [42].

No other outcomes are available for this type of smoking cessation program.

3.3.25. Pharmacological therapy plus Telephone counseling

The smoking cessation strategy based on pharmacological therapy and telephone counseling was reported by nine studies [34,42,52,53,56,64,67,71,80].

It was conducted on 1456 smokers [34,35,42,52,53,56,64,67,71], 247 of them had HIV [52], and 300 were hospitalized patients for not defined multiple diseases [42]; the range age was reported only for 187 smokers (49-75 years old), who smoked more than 20 packs per year [56].

The smoking cessation status after the intervention was recorded for 256 subjects who smoked a mean of 11.1 cigarettes per day at 12 months [80].

The smoking cessation programs had a duration higher than 3 months for 503 smokers [52,80], and 12 months for 300 smokers [42], while for the other 653, the duration was not specified.

The 6 months CARs self-reported at 6 months were recorded in 154 smokers and amounted to 40 (25.97%) former smokers [67].

The 12 months CARs verified by CO test at 12 months were recorded in 187 subjects and amounted to 37 (19.79%) former smokers [56].

The 7 days PPA self-reported at 12 months was recorded in 256 smokers and amounted to 87 (33.98%) former smokers [80].

The 7 days PPA verified by CO or cotinine test at 12 months was recorded in 503 smokers and amounted to 94 (18.69%) former smokers [80].

The 30 days PPA self-reported at 6 months was recorded in 612 smokers and amounted to 106 (17.32%) former smokers [34,42,53,64,71].
The 30 days PPA verified by CO test at 6 months was recorded in 300 smokers and amounted to 38 (12.67%) former smokers [42].
The 30 days PPA self-reported by 12 months was recorded in 300 smokers and amounted to 37 (12.33%) former smokers [42].
The 30 days PPA verified by CO test by 12 months was recorded in 300 smokers and amounted to 34 (11.33%) former smokers [42].
The adherence rate was reported for 466 subjects and was 78.97% at 6 months (the characteristics of smoking behaviour of 368 subjects were assessed at 6 months follow-up) [34,53,64,67,71].
No other outcomes are available for this type of smoking cessation program.

3.3.26. Pharmacological therapy plus Mobile phone Text-messages plus Telephone counseling

The smoking cessation strategy based on pharmacological therapy plus mobile phone text-messages plus telephone counseling was reported by two studies [44,50].
It was conducted on 376 smokers [44,50].
The 30 days PPA verified by cotinine test at 6 months amounted to 56 (14.89%) former smokers [44,50].
The adherence rate was 58.51% at 6 months (220 subjects completed the programs and agreed to perform the cotinine test at 6 months follow-up) [44,50].
No other outcomes are available for this type of smoking cessation program.

3.3.27. Pharmacological therapy plus Mobile phone App plus Telephone counseling plus Mobile CO checker

The smoking cessation strategy based on pharmacological therapy plus mobile phone App plus telephone counseling plus mobile CO checker was reported by one study [67].
It was conducted on 156 smokers [67].
The 6 months CARs self-reported at 6 months amounted to 38 (24.36%) former smokers [67].
The adherence rate was 75.64% at 6 months (the characteristics of smoking behaviour of 118 subjects were assessed at 6 months follow-up) [67].
No other outcomes are available for this type of smoking cessation program.

3.3.28. Pharmacological therapy plus Quitline

The smoking cessation strategy based on pharmacological therapy plus quitline was reported by two studies [42,47].
It was conducted on 1880 smokers [42,47], 476 of them had not defined psychiatric diseases [47], while the remaining 1440 were hospitalized patients for not-defined multiple diseases [42]. The mean age was reported only for 101 smokers and was 37.2 years old [47]; the gender ratio was reported only for 101 smokers and was 1.15 male to 1 female (54 male and 47 female) [47].
The characteristics of smoking behaviour before the intervention were recorded for 101 subjects who smoked a mean of 23.7 cigarettes per day and with a mean FTND of 5.6 reduced to 0.1 after the program [47], while the characteristics of smoking behaviour before the intervention were recorded for 476 subjects who smoked a mean of 4.27 ciga-rettes per day [47].
The smoking cessation programs had a duration of 4 months for 476 smokers [47], while for the other 1440, the duration was not specified.
The 6 months CARs self-reported at 6 months were recorded in 877 smokers and amounted to 215 (26.51%) former smokers [42].

The 7 days PPA self-reported at 6 months was recorded in 726 smokers and amounted to 190 (26.17%) former smokers [42].
The 7 days PPA verified by CO and/or cotinine tests at 6 months was recorded in 1880 smokers and amounted to 307 (16.33%) former smokers [42,47].
No other outcomes are available for this type of smoking cessation program.

3.3.29. Pharmacological therapy plus Quitline plus Fax

The smoking cessation strategy based on pharmacological therapy plus quitline plus fax was reported by one study [42].
It was conducted on 527 smokers, all of them hospitalized with not specified multiple diseases, and smoking more than five cigarettes per day [42].
The 7 days PPA self-reported at 6 months amounted to 133 (25.3%) former smokers [42].
The 7 days PPA verified by CO test at 6 months amounted to 114 (21.6%) former smokers [42].
No other outcomes are available for this type of smoking cessation program.

3.3.30 Pharmacological therapy plus IVR plus Fax

The smoking cessation strategy based on pharmacological therapy plus fax was reported by one study [42].
It was conducted on 198 smokers, all of them hospitalized with not specified multiple diseases [42].
The smoking cessation programs had a duration longer than three months [42].
The 6 months CARs self-reported at 6 months amounted to 54 (27.3%) former smokers [42].
The 7 days PPA self-reported at 6 months amounted to 81 (40.9%) former smokers [42].
The 7 days PPA verified by CO or cotinine test at 6 months amounted to 51 (25.77%) former smokers [42].
No other outcomes are available for this type of smoking cessation program.

3.3.31. Pharmacological therapy plus Website resources

The smoking cessation strategy based on pharmacological therapy plus website resources was reported by three studies [53,64,71].
It was conducted on 690 smokers, all of them were cancer survivors, in a range age of 18-55 years old, and a gender ratio of 1.19 male to 1 female (375 female and 315 female) [53,64,71].
The smoking cessation programs had a duration of 6 months [53,64,71].
The 30 days PPA self-reported at 15 months amounted to 66 (9.57%) former smokers [53,64,71].
The adherence rate was 55.39% at 15 months (the characteristics of smoking behaviour of 382 subjects were assessed at 15 months follow-up) [53,64,71].
No other outcomes are available for this type of smoking cessation program.

3.3.32. Pharmacological therapy plus Website-based counseling plus Website resources

The smoking cessation strategy based on pharmacological therapy plus website resource plus website counseling was recorded by two studies [53,64].
It was conducted on 406 smokers [53,64].
The 7 days PPA self-reported at 12 months amounted to 14 (3.45%) former smokers [53,64].
The adherence rate was 74.88% at 12 months (the characteristics of smoking behaviour of 304 subjects were assessed at 12 months follow-up) [53,64].
No other outcomes are available for this type of smoking cessation program.

3.3.33. Pharmacological therapy plus Website resource plus Telephone or Email or Internet-based counseling

The smoking cessation strategy based on pharmacological therapy plus website resources plus telephone or email or internet-based counseling was reported by four studies [34,53,64,71].
It was conducted on 268 smokers [34,53,64,71].
The smoking cessation programs had a duration of more than one month [34,53,64,71].
The 30 days PPA self-reported at 6 months amounted to 32 (11.94%) former smokers [34,53,64,71].
The adherence rate was 68.66% at 6 months (the characteristics of smoking behaviour of 184 subjects were assessed at 6 months follow-up) [34,53,64,71].
No other outcomes are available for this type of smoking cessation program.

3.3.34. Pharmacological therapy plus Website resource plus Mobile phone Text-messages plus IVR plus Quitline plus Email

The smoking cessation strategy based on pharmacological therapy plus website resources plus mobile phone text-messages plus IVR plus quitline plus email was re-ported by nine studies [41,50,51,53,61,64,71,72,74].
It was conducted on 1773 smokers with a mean age of 35.9 years old and a gender ratio of 1 male to 1.03 female (873 male and 900 female), who smoked more than ten cigarettes per day with a mean of 18.3 cigarettes per day and a mean FTND of 4.8 [41,50,51,53,61,64,71,72,74].
The 7 days PPA self-reported at 6 months amounted to 657 (37.06%) former smokers [41,50,51,53,61,64,71,72,74].
The 7 days PPA self-reported at 12 months amounted to 666 (37.56%) former smokers [41,50,51,53,61,64,71,72,74].
The adherence rate to pharmacological therapy was 91.99% (1631 smokers completed the pharmacological therapy prescribed) and 87.81% at 12 months (the smoking cessation status of 1557 was assessed at 12 months) [41,50,51,53,61,64,71,72,74].

3.3.35. Pharmacological therapy plus Internet-based contest

The smoking cessation strategy based on pharmacological therapy plus inter-net-based contest was reported by one study [66].
It was conducted on 1217 smokers with a mean age of 26.30 years old and a gender ratio of 1 male to 1.22 female (549 male and 668 female), who smoked more than ten cigarettes per week for at least one month with a mean of 11.55 cigarettes per day [66].
The smoking cessation program had a duration of 4 months [66].
The 6 months CARs verified by cotinine test at 6 months amounted to 71 (5.83%) former smokers [66].
The 30 days PPA verified by cotinine at 6 months amounted to 153 (12.57%) former smokers [66].
No other outcomes are available for this type of smoking cessation program.

3.3.36. Pharmacological therapy plus Internet-based contest plus Telephone counseling

The smoking cessation strategy based on pharmacological therapy plus inter-net-based contest plus telephone counseling was reported by one study [66].
It was conducted on 1217 smokers with a mean age of 26.25 years old and a gender ratio of 1 male to 1.22 female (549 male and 668 female), who smoked more than ten cigarettes per week for at least one month with a mean of 11.49 cigarettes per day [66].
The smoking cessation program had a duration of 4 months [66].
The 6 months CARs verified by cotinine test at 6 months amounted to 71 (5.83%) former smokers [66].
The 30 days PPA verified by cotinine at 6 months amounted to 153 (12.57%) former smokers [66].

No other outcomes are available for this type of smoking cessation program.

3.3.37. Pharmacological therapy plus Telephone counseling plus IVR plus Video materials plus Mobile CO checker

The smoking cessation strategy based on pharmacological therapy plus telephone counseling plus IVR plus video materials plus CO checker was reported by one study [67].

It was conducted on 90 smokers with a mean age of 45 years old and a gender ratio of 1 male to 1.43 female (37 male and 53 female), smoking more than ten cigarettes per day [67].

The 6 months CARs verified by CO test at 6 months amounted to 15 (16.67%) former smokers [67].

The adherence rate was 90.00% at 6 months (the characteristics of smoking behaviour of 81 subjects were assessed at 6 months follow-up) [67].

No other outcomes are available for this type of smoking cessation program.

3.3.38. Pharmacological therapy plus Internet-based resources plus Internet-based Text-message

The smoking cessation strategy based on pharmacological therapy plus internet-based resources plus internet-based text-messages was reported by five studies [41,53,64,71,72].

It was conducted on 5795 smokers with an age range of 30-50 years old and a gender ratio of 1 male to 2.27 female (1770 male and 4025 female) [41,53,64,71,72].

The 6 months CARs self-reported at 6 months amounted to 225 (3.80%) former smokers [41,53,64,71,72].

The 7 days PPA self-reported at 6 months amounted to 560 (9.66%) former smokers [41,53,64,71,72].

The adherence rate was 27.35% at 6 months (the characteristics of smoking behaviour of 1585 were assessed at 6 months follow-up) [41,53,64,71,72].

No other outcomes are available for this type of smoking cessation program.

3.3.39. Pharmacological therapy plus Physical exercises program plus Internet-based re-sources plus Internet-based Text-message

The smoking cessation strategy based on pharmacological therapy plus physical exercises program plus internet-based resources plus internet-based text-messages was reported by five studies [41,53,64,71,72].

It was conducted on 5795 smokers with an age range of 30-50 years old and a gender ratio of 1 male to 2.51 female (1650 male and 4145 female) [41,53,64,71,72].

The 6 months CARs self-reported at 6 months amounted to 225 (3.88%) former smokers [41,53,64,71,72].

The 7 days PPA self-reported at 6 months amounted to 600 (10.35%) former smokers [41,53,64,71,72].

The adherence rate was 27.09% at 6 months (the characteristics of smoking behaviour of 1569 were assessed at 6 months follow-up) [41,53,64,71,72].

No other outcomes are available for this type of smoking cessation program.

3.3.40 Pharmacological therapy plus Printed materials plus Telephone counseling

The smoking cessation strategy based on pharmacological therapy plus printed materials plus telephone counseling was reported by six studies [34,37,41,70,78,79].

It was conducted on 848 smokers [34,37,41,70,78,79]; the age range was reported only for 558 smokers and was 50-74 years old [70,78].

The characteristics of smoking behaviour before the intervention were recorded for 1144 subjects who smoked more than five cigarettes per day for at least one year [34,37,41,79].

The 12 months CARs verified by cotinine test at 12 months were recorded in 1144 smokers and amounted to 84 (29.37%) former smokers [34,37,41,79].

The 7 days PPA self-reported at 12 months was recorded in 558 smokers and amounted to 86 (15.41%) former smokers [70,78].
The 7 days PPA verified by cotinine test at 12 months was recorded in 1144 smokers and amounted to 136 (11.89%) former smokers [34,37,41,79].
The adherence rate was reported for 1144 subjects and was 37.3% (427 subjects completed all four counseling sessions, while the adherence at one counseling session was 83.5%, 76.1% at two, and 60.6% at three) [34,37,41,79].
No other outcomes are available for this type of smoking cessation program.

3.3.41. Pharmacological therapy plus Printed materials plus Telephone counseling plus Quitline

The smoking cessation strategy based on pharmacological therapy plus printed materials plus telephone counseling plus quitline was reported by three studies [64,68,71].
It was conducted on 1040 smokers [64,68,71], 236 of them with HIV [68], smoking more than 5 cigarettes per day [64,68,71].
The smoking cessation programs had a duration of major of 3 months for 236 smokers [68], while for the other 804, the duration was not specified [64,71].
The 7 days PPA self-reported at 6 months was recorded in 236 smokers and amounted to 3 (1.27%) former smokers [68].
The 30 days PPA self-reported at 6 months was recorded in 804 smokers and amounted to 246 (30.60%) former smokers [64,71].
The adherence rate was reported for 804 subjects and was 73.38% at 6 months (the characteristics of smoking behaviour of 590 subjects were assessed at 6 months follow-up) [64,71].
No other outcomes are available for this type of smoking cessation program.

3.3.42. Pharmacological therapy plus Printed materials plus Mobile phone Text-messages plus Quitline

The smoking cessation strategy based on pharmacological therapy plus printed materials plus mobile text-messages plus quitline was reported by two studies [67,68].
It was conducted on 214 smokers, all of them affected by HIV, and smoking more than five cigarettes per day [67,68].
The 7 days PPA verified by CO test at 6 months amounted to 8 (3.74%) former smokers [67,68].
The adherence rate was 71.96% at 6 months (the characteristics of smoking behaviour of 154 subjects were assessed at 6 months follow-up) [67,68].
No other outcomes are available for this type of smoking cessation program.

3.3.43. Pharmacological therapy plus Printed materials plus Computer-based Video counseling

The smoking cessation strategy based on pharmacological therapy plus printed materials plus computer-based video counseling was reported by four studies [34,37,41,79].
It was conducted on 1120 smokers, who smoked more than five cigarettes per day for at least one year [34,37,41,79].
The 12 months CARs verified by cotinine test at 12 months amounted to 82 (8.2%) former smokers [34,37,41,79].
The 7 days PPA verified by cotinine test at 12 months amounted to 110 (9.8%) former smokers [34,37,41,79].
The adherence rate was 32.3% (362 subjects completed all four counseling sessions, while the adherence at one counseling session was 79.7%, 68.5% at two, and 55.1% at three) [34,37,41,79].
No other outcomes are available for this type of smoking cessation program.

3.3.44. Pharmacological therapy plus Printed materials plus Computer-based Audio self-interview

The smoking cessation strategy based on pharmacological therapy plus printed materials plus computer-based audio self-interview was reported by one study [68].

It was conducted on 238 smokers, all of them had HIV and smoked more than five cigarettes per day [68].

The 7 days PPA self-reported at 6 months amounted to 5 (2.10%) former smokers [68].

No other outcomes are available for this type of smoking cessation program [68].

3.3.45. Pharmacological therapy plus Printed materials plus Website resources plus Website Text-messages plus Quitline

The smoking cessation strategy based on pharmacological therapy plus printed materials plus website resources plus website text-messages plus quitline was reported by two studies [64,71].

It was conducted on 802 smokers, who smoked more than ten cigarettes per day for at least one year and more than five cigarettes per day for at least one week [64,71].

The 30 days PPA self-reported at 6 months amounted to 220 (27.43%) former smokes [64,71].

The adherence rate was 74.31% at 6 months (the characteristics of smoking behaviour of 596 subjects were assessed at 6 months follow-up) [64,71].

No other outcomes are available for this type of smoking cessation program.

3.3.46. Pharmacological therapy plus Printed materials plus Website resources plus Website Text-messages plus Telephone counseling plus Quitline

The smoking cessation strategy based on pharmacological therapy plus printed materials plus website resources plus website text-messages plus telephone counseling plus quitline was reported by two studies [64,71].

It was conducted on 804 smokers, who smoked more than ten cigarettes per day for at least one year and more than five cigarettes per day for at least one week [64,71].

The 30 days PPA self-reported at 6 months amounted to 246 (30.60%) former smokes [64,71].

The adherence rate was 73.38% at 6 months (the characteristics of smoking behaviour of 590 subjects were assessed at 6 months follow-up) [64,71].

No other outcomes are available for this type of smoking cessation program.

3.4 Non-Pharmacological Therapy Smoking Cessation Interventions

3.4.1. In-person counseling plus Mobile phone Text-messages

The smoking cessation strategy based on in-person counseling plus mobile phone text-messages by one study [67].

It was conducted on 30 smokers [67].

The smoking cessation program had a duration of 4 months [67].

The 7 days PPA self-reported at 6 months amounted to 6 (20.00%) former smokers [67].

The adherence rate was 90.00% at 6 months (the characteristics of smoking behaviour of 27 subjects were assessed at 6 months follow-up) [67].

No other outcomes are available for this type of smoking cessation program.

3.4.2. In-person counseling plus IVR

The smoking cessation strategy based on in-person counseling plus IVR by one study [42].

It was conducted on 50 smokers, all of them were hospitalized patients for coronary diseases [42].

The 7 days PPA self-reported at 12 months amounted to 23 (46.00%) former smokers [42].

No other outcomes are available for this type of smoking cessation program.

3.4.3. In-person counseling plus Quitline

The smoking cessation strategy based on in-person counseling plus quitline was reported by two studies [42,46].

It was conducted on 446 smokers [42,46], all of them were hospitalized [42,46], and in particular, 145 were hospitalized patients pre-surgery (not cardiac surgery) [46].

The 30 days PPA self-reported at 6 months was recorded in 301 smokers and amounted to 66 (22%) former smokers [42].

The 7 days PPA at 12 months was recorded in 145 smokers and amounted to 38 (26.21%) former smokers, but methods of verification were not specified [46].

No other outcomes are available for this type of smoking cessation program.

3.4.4. In-person counseling plus Website resources

The smoking cessation strategy based on in-person counseling plus website resources was reported by four studies [42,53,60,64].

It was conducted on 2377 smokers [42,53,60,64], 2244 of them were hospitalized patients for not defined diseases [42,53,64]; the age range was specified only for 133 smokers, who were 18-24 years old [60].

The 7 days PPA self-reported at 6 months was recorded in 2244 smokers and amounted to 570 (25.40%) former smokers [42,53,64].

The 30 days PPA self-reported at 6 months was recorded in 133 smokers and amounted to 30 (22.56%) former smokers [60].

The adherence rate was reported for 2244 subjects and was 81.15% at 6 months (the characteristics of smoking behaviour of 1821 subjects were assessed at 6 months follow-up) [42,53,64].

No other outcomes are available for this type of smoking cessation program.

3.4.5. In-person counseling plus Video resources

The smoking cessation strategy based on in-person counseling plus video resources was reported by one study [15]
It was conducted on 675 smokers [77].
The 7 days PPA verified by cotinine test at 12 months amounted to 31 (4.59%) former smokers [15]
No other outcomes are available for this type of smoking cessation program.

3.4.6. In-person counseling plus Internet-based photo-aging software

The smoking cessation strategy based on in-person counseling plus internet-based photo-aging software was reported by five studies [40,50,53,64,74].
It was conducted on 400 smokers with a mean age of 24.2 years old and a gender ratio of 1 male to 2.20 female (125 male and 275 female), with a mean FTND of 2.87 [40,50,53,64,74].
The smoking cessation programs had a duration of 12 months [40,50,53,64,74].
The 2 days PPA verified by CO test at 6 months amounted to 55 (13.75%) former smokers [40,50,53,64,74].
No other outcomes are available for this type of smoking cessation program.

3.4.7. In-person counseling plus Virtual reality eyewear

The smoking cessation strategy based on in-person counseling plus virtual reality eyewear was reported by one study [63].
It was conducted on 50 smokers with a mean age of 39.68 years old and a gender ratio of 1 male to 2.57 female (14 male and 36 female), smoking a mean of 18.7 cigarettes per day and with a mean FTND of 5.1 [63].
The 6 months CARs verified by CO test at 6 months amounted to 9 (18%) former smokers [63].
The 12 months CARs verified by CO test at 12 months amounted to 8 (16%) former smokers [63].
The 7 days PPA verified by CO test at 6 months amounted to 12 (24%) former smokers [63].
The 7 days PPA verified by CO test at 12 months amounted to 11 (22%) former smokers [63].
The adherence rate was 78%, but the timing was not specified (39 smokers completed all the programmed in-person counseling sessions) [63].
No other outcomes are available for this type of smoking cessation program.

3.4.8. In-person counseling plus Mobile phone App

The smoking cessation started based on in-person counseling plus mobile phone App was reported by two studies [32,39].
It was conducted on 155 smokers with a mean age of 37.35 years and a gender ratio of 1.5 male to 1 female (93 male and 62 female) [32,39].
The characteristics of smoking behaviour before the intervention were recorded for 100 subjects who smoked more than ten cigarettes per day for at least 12 months [32,39]. The nicotine addiction severity was registered for 55 subjects through the Tobacco Dependence Scale, which was higher than five [32].
The smoking cessation programs had a duration of 6 weeks for 100 smokers [32,39], while the other 55 [32], the duration was not specified.
The 4 months CARs verified by CO test at 6 months were recorded in 55 smokers and amounted to 35 (64%) former smokers [32].
The 10 months CARs verified by CO test at 12 months were recorded in 55 smokers and amounted to 32 (58%) former smokers [32].
The 7 days PPA verified by CO test at 7 months were recorded in 100 smokers and amounted to 24 (24.00%) former smokers [32,39].
No other outcomes are available for this type of smoking cessation program.

3.4.9. In-person counseling plus Mobile phone App plus Mobile CO checker

The smoking cessation strategy based on in-person counseling plus mobile phone App plus mobile CO checker was reported by one study [37].

It was conducted on 57 smokers [37].

The 4 months CARs verified by CO test at 6 months amounted to 41 (71.9%) former smokers [37].

The adherence rate was 95% at 6 months (the characteristics of smoking behaviour of 54 subjects were assessed at 6 months follow-up), 96% at 2 weeks, 1, 2, and 3 months (the characteristics of smoking behaviour of 55 subjects were assessed at 1,2, and 3 months follow-up) [37].

No other outcomes are available for this type of smoking cessation program.

3.4.10. In-person counseling plus Telephone counseling

The smoking cessation strategy based on in-person counseling plus telephone counseling was reported by five studies [31,38,42,46,57].

It was conducted on 2003 smokers [31,38,42,46,57], 1542 of them were hospitalized patients, in particular, 320 were hospitalized for acute coronary syndrome [31], 119 were hospitalized for not cardiac pre-surgery [57], 71 were hospitalized for pre-cardiac surgery [46], and 54 were hospitalized for acute myocardial infarction [42].

The characteristics of smoking behaviour before the intervention were recorded for 51 subjects who smoked at least ten packs per year and had an FTND higher than 4 [31], 135 smoked more than five cigarettes per day for at least one year [31], 94 smoked more than one cigarette per day for at least one hundred days [31], 320 smoked more than one cigarette per day for at least six months, 119 smoked at least ten cigarettes per day for at least one year [31], and 181 smoked more than ten cigarettes per day for at least one month [31].

The smoking cessation programs had a duration of more than 7 weeks for 135 subjects [31], 6 months for 387 subjects [31,42], 12 months for 252 subjects [42,46], while for the other 1229, the duration was not specified.

The 4 months CARs verified by CO test at 6 months were recorded in 320 smokers and amounted to 62 (19.38%) former smokers [31].

The 5 months CARs verified by CO test at 6 months were recorded in 94 smokers and amounted to 22 (23.40%) former smokers [31].

The 6 months CARs verified by CO test at 6 months were recorded in 51 smokers and amounted to 6 (11.76%) former smokers [31].

The 12 months CARs verified by CO test at 12 months were recorded in 135 smokers and amounted to 16 (11.85%) former smokers [31].

The 7 days PPA self-reported at 6 months was recorded in 336 smokers and amounted to 132 (39.29%) former smokers [31,38,42].

The 7 days PPA verified by CO or cotinine test at 6 months was recorded in 119 smokers and amounted to 31 (26.05%) former smokers [57].

The 7 days PPA self-reported at 12 months was recorded in 750 smokers and amounted to 168 (22.40%) former smokers [42].

The 7 days PPA verified by cotinine test at 12 months was recorded in 696 smokers and amounted to 69 (9.91%) former smokers [42].

The PPA at 12 months was recorded in 71 smokers and amounted to 24 (33.80%) former smokers, but time and methods of verification were not defined [46].

No other outcomes are available for this type of smoking cessation program.

3.4.11. In-person counseling plus Telephone counseling plus Video counseling

The smoking cessation strategy based on in-person counseling plus telephone counseling plus video counseling was reported by one study [46].
It was conducted on 40 smokers, all of them were hospitalized for pre-coronary artery bypass [46].
The PPA at 12 months amounted to 24 (60.00%) former smokers, but the time and methods of verification were not defined [46].
No other outcomes are available for this type of smoking cessation program.

3.4.12. In-person counseling plus Telephone counseling plus Computer-based Text-messages

The smoking cessation strategy based on in-person counseling plus telephone counseling plus computer-based text-messages was reported by one study [64].
It was conducted on 132 smokers [64].
The 6 months CARs verified by CO test at 12 months amounted to 20 (15.15%) former smokers [64].
The adherence rate was 56.82% at 12 months (the characteristics of smoking behaviour of 75 subjects were assessed at 12 months follow-up) [64].
No other outcomes are available for this type of smoking cessation program.

3.4.13. In-person counseling plus Printed materials plus Email

The smoking cessation strategy based on in-person counseling plus printed materials plus email was reported by four studies [35,45,66,78].
It was conducted on 1569 smokers [35,45,66,78]; the mean age was reported for 144 smokers and was 19.8 years (range 18-23) [35,45,66]; the gender ratio was reported for 144 smokers and was 1.09 male to 1 female (75 male and 69 female) [35,45,66].
The characteristics of smoking behaviour before the intervention were recorded for 144 subjects who smoked a mean of 9.9 cigarettes per day [35,45,66].
The 7 days PPA self-reported at 6 months was recorded in 144 smokers and amounted to 36 (25.00%) former smokers [35,45,66].
The 7 days PPA verified by cotinine test at 6 months was recorded in 144 smokers and amounted to 15 (10.42%) former smokers [35,45,66].
The 30 days PPA self-reported at 12 months were recorded in 1425 smokers and amounted to 95 (6.67%) former smokers [78].
The adherence rate was reported for 144 subjects and was 91.6% (132 subjects read all/most emails, furthermore, 48% responded to emails) [35,45,66].
No other outcomes are available for this type of smoking cessation program.

3.4.14. In-person counseling plus Printed materials plus Mobile phone Text-messages

The smoking cessation strategy based on in-person counseling plus printed materials plus mobile phone text-messages was reported by three studies [49,67,80].
It was conducted on 260 smokers [49,67,80]; the gender ratio was reported for 228 smokers and was 228 male to 0 female (228 male and 0 female) [49,67].
The smoking cessation programs had a duration major of 6 months for 32 smokers [80], while for the other 228, the duration was not specified.
The 7 days PPA self-reported at 6 months was recorded in 32 smokers and amounted to 11 (34%%) former smokers [80].
The PPA self-reported at 6 months was recorded in 228 smokers and amounted to 40 (17.54%) former smokers, but the time of verification was not specified [49,67].

The PPA self-reported at 12 months was recorded in 228 smokers and amounted to 44 (19.30%) former smokers, but the time of verification was not specified [49,67].
The adherence rate was reported for 228 subjects and was 85.09% at 12 months (the characteristics of smoking behaviour of 194 subjects were assessed at 12 months follow-up) [49,67].
No other outcomes are available for this type of smoking cessation program.

3.4.15. In-person counseling plus Printed materials plus Telephone counseling

The smoking cessation strategy based on in-person counseling plus printed materials plus telephone counseling by two studies [46,70].
It was conducted on 36 smokers [46,70], 14 of them were hospitalized patients for pre-coronary artery bypass surgery and all these 14 patients were female [46]; the age range was recorded only for 22 subjects and was higher than 50 years old [70].
The smoking cessation had a duration of 3 months for 22 smokers [70], while for the other 14, the duration was not specified.
The PPA at 7 months was recorded in 22 smokers and amounted to 6 (27.27%) former smokers, but time and methods of verification were not defined [70].
The PPA at 12 months amounted to 16 (44.44%) former smokers, but time and methods of verification were not defined [46,70].
No other outcomes are available for this type of smoking cessation program.

3.4.16. In-person counseling plus Printed materials plus Mobile phone Text-messages plus Telephone counseling

The smoking cessation strategy based on in-person counseling plus printed materials plus mobile phone text-messages plus telephone counseling was reported by one study [44].
It was conducted on 679 smokers [44].
The 7 days PPA self-reported at 6 months amounted to 138 (20.32%) former smokers [44].
The 7 days PPA self-reported at 12 months amounted to 155 (22.83%) former smokers [44].
No other outcomes are available for this type of smoking cessation program.

3.4.17. In-person counseling plus Printed materials plus Television series

The smoking cessation strategy based on in-person counseling plus printed materials plus television series [77].
It was conducted on 283 smokers [77].
The 12 months CARs verified by cotinine test at 12 months amounted to 34 (12.01%) former smokers [77].
No other outcomes are available for this type of smoking cessation program.

3.4.18. In-person counseling plus Printed materials plus Television series plus Telephone counseling

The smoking cessation strategy based on in-person counseling plus printed materials plus television series plus telephone counseling was reported by one study [77].
It was conducted on 380 smokers [77].
The PPA self-reported at 12 months amounted to 26 (6.84%) former smokers, but the time of verification were not specified [77].
No other outcomes are available for this type of smoking cessation program.

3.4.19. In-person counseling plus Printed materials plus Audio materials plus Quitline

The smoking cessation strategy based on in-person counseling plus printed materials plus audio materials plus quitline was reported by two studies [48,69].
It was conducted on 56 smokers with a mean age of 63 years old and a gender ratio of 1.8 male to 1 female (36 male and 20 female) [48,69].
The PPA self-reported at 12 months amounted to 8 (14.29%) former smokers, but the time of verification was not defined [48,69].
The PPA verified by CO test at 12 months amounted to 2 (3.57%) former smokers, but the time of verification was not defined [48,69].
No other outcomes are available for this type of smoking cessation program.

3.4.20. In-person counseling plus Printed materials plus Physical exercises program plus Telephone counseling

The smoking cessation strategy based on in-person counseling plus printed materials plus physical exercise program plus telephone counseling was reported by one study [70].
It was conducted on 30 smokers [70].
The smoking cessation program had a duration of 3 months [70].
The PPA at 7 months amounted to 4 (22.22%) former smokers, but time and methods of verification were not defined [70].
The PPA at 12 months amounted to 5 (16.67%) former smokers, but time and methods of verification were not defined [70].
No other outcomes are available for this type of smoking cessation program.

3.4.21. Printed materials plus Television series

The smoking cessation strategy based on printed materials plus television series was reported by two study [75,77].
It was conducted on 828 smokers [75,77].
The 12 months CARs verified by cotinine test at 12 months were recorded in 281 smokers and amounted to 10 (3.56%) former smokers [77].
The PPA self-reported at 12 months was recorded in 109 smokers and amounted to 4 (3.36%) former smokers, but the time of verification was not specified [77].
The 7 days PPA self-reported at 24 months was recorded in 438 smokers and amounted to 105 (23.97%) former smokers [75].
No other outcomes are available for this type of smoking cessation program.

3.4.22. Printed materials plus the Creation of a group video message

The smoking cessation strategy based on printed materials plus the creation of a group video message was reported by four studies [53,55,64,66].
It was conducted on 336 smokers with a mean age of 20.35 years old (range 18-24) and a gender ratio of 1.90 male to 1 female (220 male and 116 female) [53,55,64,66].
The 7 days PPA verified by CO test at 6 months amounted to 76 (22.62%) former smokers [53,55,64,66].
The 30 days PPA verified by CO test at 6 months amounted to 52 (15.48%) former smokers [53,55,64,66].
The adherence rate was 95.2% at 6 months (the characteristics of smoking behaviour of 320 subjects were assessed at 6 months follow-up) [53,55,64,66].
No other outcomes are available for this type of smoking cessation program.

3.4.23. Printed materials plus Mobile phone Text-messages

The smoking cessation strategy based on printed materials plus mobile phone text-messages was reported by three study [50,54,67,68,73].
It was conducted on 410 smokers with a mean age of 40.75 years old [50,54,67,68,73]; the gender ratio was reported for 284 subjects and was 1 male to 1.12 female (134 male and 150 female), who smoked more than ten cigarettes per day for at least three months with a mean of 18.3 cigarettes per day and a mean FTND 4.8 [67,68].
The smoking cessation programs had a duration of 2 months for 126 smokers [50,54,73], while for the other 284, the duration was not specified.
The 7 days PPA self-reported at 6 months was recorded in 126 smokers and amounted to 51 (40.48%) former smokers [50,54,73].
The 7 days PPA verified by CO test at 6 months was recorded in 284 smokers and amounted to 26 (9.15%) former smokers [67,68].
The adherence rate was reported for 284 subjects and was 84.51% at 6 months (the characteristics of smoking behaviour of 240 subjects were assessed at 6 months follow-up) [67,68].
No other outcomes are available for this type of smoking cessation program.

3.4.24. Printed materials plus Social media Text-messages

The smoking cessation strategy based on printed materials plus social media text-messages was reported by three studies [50,54,73].
It was conducted on 120 smokers with a mean age of 37.6 years old [50,54,73].
The smoking cessation programs had a duration of 2 months [50,54,73].
The 7 days PPA self-reported at 6 months amounted to 63 (52.50%) former smokers [50,54,73].
No other outcomes are available for this type of smoking cessation program.

3.4.25. Printed materials plus Telephone counseling

The smoking cessation strategy based on printed materials plus telephone counseling was reported by five studies [58,66,67,70,75].
It was conducted on 4386 smokers [58,66,67,70,75]; the mean age was reported only for 505 smokers and was 61 years (age range 50-74 years) [70], 622 smokers had an age range of 18-25 years old [66,75], while for the other 3259, it was not specified; the gender ratio was reported only for 923 smokers and was of 1 male to 1.68 female (344 male and 579 female) [66,70,75].
The characteristics of smoking behaviour before the intervention were recorded for 920 subjects who smoked a mean of 22.14 cigarettes per day [66,70,75].
The smoking cessation programs had a duration of 4-6 weeks for 418 smokers [66,75], while for the other 3968, the duration was not specified.
The 6 months CARs self-reported at 6 months were recorded in 2248 smokers and amounted to 368 (16.37%) former smokers [58,75].
The 2 days PPA self-reported at 6 months was recorded in 204 smokers and amounted to 20 (9.80%) former smokers [66].
The 7 days PPA self-reported at 6 months was recorded in 418 smokers and amounted to 28 (6.70%) former smokers [66,75].
The 7 days PPA verified by CO and cotinine test at 12 months was recorded in 1011 smokers and amounted to 42 (4.15%) former smokers [67,75].
The PPA at 12 months was recorded in 505 smokers and amounted to 96 (19.01%) former smokers, but the time and methods of verification were not specified [70].

The adherence rate was reported for 1011 subjects and was 65.68% at 12 months (the characteristics of smoking behaviour of 664 subjects were assessed at 12 months follow-up) [67,75].
No other outcomes are available for this type of smoking cessation program.

3.4.26. Printed materials plus Quitline
The smoking cessation strategy based on printed materials plus quitline was reported by two studies [48,69].
It was conducted on 54 smokers with a mean age of 63 years old and a gender ratio of 1.7 male to 1 female (34 male and 20 female) [48,69].
The PPA self-reported at 12 months amounted to 10 (18.52%) former smokers, but the time of verification was not defined [48,69].
The PPA verified by CO test at 12 months amounted to 6 (11.11%) former smokers, but the time of verification was not defined [48,69].
No other outcomes are available for this type of smoking cessation program.

3.4.27. Printed materials plus Email
The smoking cessation strategy based on printed materials plus email was reported by one study [53].
It was conducted on 552 smokers [53].
The smoking cessation programs had a duration of 6 weeks [53].
The 12 months CARs self-reported at 12 months amounted to 23 (4.17%) former smokers [53].
No other outcomes are available for this type of smoking cessation program.

3.4.28. Printed materials plus Quitline plus Email
The smoking cessation strategy based on printed materials plus quitline plus email was reported by two studies [70,75].
It was conducted on 170 smokers, all of them were older than 60 years [70,75].
The 7 days PPA self-reported at 6 months amounted to 34 (20.00%) former smokers [70,75].
The adherence rate was evaluated as smokers who called the quitline and was 42% (71 smokers) at 6 months [70,75].
No other outcomes are available for this type of smoking cessation program.

3.4.29. Printed materials plus Telephone counseling plus Quitline
The smoking cessation strategy based on printed materials plus telephone counseling plus quitline was reported by two studies [70,75].
It was conducted on 647 smokers, all of them were older than 50 years [70,75].
The 7 days PPA self-reported at 6 months was recorded in 184 smokers and amounted to 36 (19.57%) former smokers [70,75].
The 7 days PPA self-reported at 12 months was recorded in 463 smokers and amounted to 88 (19.00%) former smokers [75].
The adherence rate was recorded for 184 smokers and evaluated as smokers who spoke with the counselor through telephone counseling and was 17.5% (32 smokers) at 6 months [70,75].
No other outcomes are available for this type of smoking cessation program.

3.4.30 Printed materials plus Telephone counseling plus Video resources plus Email
The smoking cessation strategy based on printed materials plus telephone counseling plus video resources plus email was reported by one study [77].

It was conducted on 675 smokers [77].
The 7 days PPA verified by cotinine test at 12 months amounted to 22 (3.26%) former smokers [77].
No other outcomes are available for this type of smoking cessation program.

3.4.31. Printed materials plus Television series plus Quitline plus Email

The smoking cessation strategy based on printed materials plus television series plus quitline plus email was reported by one study [75].
It was conducted on 873 smokers [75].
The 7 days PPA self-reported at 24 months amounted to 180 (20.62%) former smokers [75].
The adherence rate was evaluated as subjects who called to quitline and amounted to 7% [75].
No other outcomes are available for this type of smoking cessation program.

3.4.32. Printed materials plus Website resources plus Telephone counseling plus Email

The smoking cessation strategy based on printed materials plus website resources plus telephone counseling plus email was reported by one study [45].
It was conducted on 11143 smokers with a mean age of 43 years old and a gender ratio of 1 male to 1.17 female (5126 male and 6017 female) [45].
The 30 days PPA self-reported at 6 months amounted to 2340 (21%) former smokers [45].
No other outcomes are available for this type of smoking cessation program.
