# Supplementary material for: Effectiveness and Adherence of Pharmacological vs. Non-Pharmacological Technology-Supported Smoking Cessation Interventions: An Umbrella Review
Source: Healthcare (Basel). 2025 Apr 21;13(8):953. doi: 10.3390/healthcare13080953 (PMC12027406; doi:10.3390/healthcare13080953)
Supplement: Supplementary file 1 [file healthcare-13-00953-s001.zip › Supplementary File S4 Pharmacological vs. Non-Pharmacological.pdf]

Supplementary File S4: Quality Assessment

The Table S3 shows the risk of bias and quality assessment for each included systematic review (alphabetically ordered).

A total of 17 (34.00% of the included systematic reviews) systematic reviews [33,36,37,46,53-55,62,64,65,67,68,75-79] were judged as "high quality", 5 (10.00%) systematic reviews [31,39,42,44,63] as "moderate quality", 12 (24.00%) systematic reviews [32,34,40,41,47-50,60,66,71,74] as "low quality", 16 (32.00%) systematic reviews [35,38,43,45,51,52,56-59,61,69,70,72,73,80] as "critically low quality" using the AMSTAR-2 tool.

Table S3. Quality assessment of the included systematic reviews based on the AMSTAR-2 (the AMSTAR-2 critical domains are highlighted in grey).
Abbreviations: Yes, "Y"; No, "N"; Partially Yes, "PY"; No Meta-Analysis, "NMA".

Study	1	2	3	4	5	6	7	8	9	10	11	12	13	14	15	16	Quality
Akanbi M.O., 2019 [31]	Y	Y	Y	Y	Y	Y	Y	N	Y	N	Y	Y	Y	Y	Y	Y	Moderate
Barroso-Hurtado M., 2021 [32]	Y	Y	Y	Y	Y	Y	PY	Y	Y	N	NMA	NMA	N	Y	NMA	Y	Low
Bendotti H., 2023 [33]	Y	Y	Y	Y	Y	Y	Y	Y	Y	N	Y	Y	Y	Y	Y	Y	High
Boland V.C., 2018 [34]	Y	N	Y	PY	Y	Y	PY	PY	Y	N	Y	Y	Y	Y	Y	Y	Low
Brown J., 2013 [35]	Y	N	Y	Y	N	N	N	Y	Y	N	NMA	NMA	Y	N	NMA	N	Critically Low
Brown N., 2017 [80]	Y	N	Y	PY	N	Y	N	Y	Y	N	NMA	NMA	Y	N	NMA	Y	Critically Low
Byambasuren O., 2023 [36]	Y	Y	Y	Y	Y	Y	Y	PY	Y	N	Y	Y	Y	Y	Y	Y	High
Byaruhanga J., 2020 [37]	Y	Y	Y	Y	Y	Y	PY	Y	Y	Y	Y	Y	Y	N	Y	Y	High
Cartujano-Barrera F., 2022 [38]	N	Y	Y	Y	Y	Y	N	Y	N	N	NMA	NMA	N	N	NMA	Y	Critically Low
Chhabra D., 2023 [39]	Y	Y	Y	Y	Y	Y	PY	Y	Y	N	NMA	NMA	Y	N	NMA	Y	Moderate
Crilly P., 2020 [40]	Y	N	Y	PY	Y	Y	PY	Y	Y	N	NMA	NMA	Y	N	NMA	Y	Low
da Silva Teixeira R., 2023 [41]	Y	Y	Y	Y	Y	Y	N	PY	Y	N	Y	Y	Y	Y	Y	Y	Low
do Amaral L.M,	Y	Y	Y	Y	N	Y	Y	Y	PY	N	NMA	NMA	Y	N	NMA	N	Moderate

2020 [42]																
Eghdami S.,<br>2023 [43]	Y	N	Y	Y	Y	Y	N	N	Y	N	Y	Y	Y	Y	Y	Y	Critically low
Fang Y.E.,<br>2023 [44]	Y	Y	Y	Y	Y	Y	Y	N	Y	Y	Y	N	Y	Y	Y	Y	Moderate
Gainsbury S.,<br>2011 [45]	Y	N	Y	PY	N	N	N	N	PY	N	NMA	NMA	N	N	NMA	Y	Critically Low
Graham A.L.,<br>2016 [71]	Y	N	Y	PY	Y	Y	Y	Y	Y	Y	Y	Y	Y	Y	Y	N	Low
Han M.,<br>2018 [73]	Y	N	Y	N	Y	N	PY	PY	Y	N	NMA	NMA	N	N	NMA	N	Critically Low
Harrogate S.,<br>2023 [46]	Y	Y	Y	Y	Y	Y	Y	Y	Y	Y	Y	Y	Y	Y	Y	Y	High
Hawes M.R.,<br>2021 [47]	Y	N	Y	Y	Y	Y	PY	PY	Y	N	NMA	NMA	Y	Y	NMA	Y	Low
Hutton H.E.,<br>2011 [72]	Y	N	Y	N	Y	N	N	Y	Y	N	Y	Y	Y	Y	Y	Y	Critically Low
Iaccarino J.M.,<br>2019 [48]	Y	N	Y	Y	N	Y	Y	PY	Y	N	NMA	NMA	Y	Y	NMA	Y	Low
Kant R.,<br>2021 [74]	Y	N	Y	PY	Y	N	PY	N	Y	N	Y	Y	Y	Y	Y	Y	Low
Krishnan N.,<br>2021 [49]	Y	N	Y	Y	Y	Y	PY	Y	Y	N	NMA	NMA	Y	N	NMA	Y	Low
Li S.,<br>2024 [50]	Y	N	Y	Y	Y	Y	PY	PY	Y	N	Y	Y	Y	Y	Y	Y	Low
Liu S.,<br>2017 [51]	Y	N	Y	N	N	N	N	N	PY	N	Y	N	N	Y	N	Y	Critically Low
Mann-Jackson L.,<br>2019 [52]	N	N	N	PY	N	N	N	N	N	N	NMA	NMA	N	N	NMA	Y	Critically Low
Matkin W.,<br>2019 [75]	Y	Y	Y	Y	Y	Y	Y	Y	Y	Y	Y	Y	Y	Y	Y	Y	High
McCrabb S.,<br>2019 [53]	Y	Y	Y	Y	Y	Y	PY	Y	Y	N	Y	Y	Y	Y	Y	Y	High
Naslund J.A.,<br>2017 [54]	Y	Y	Y	Y	Y	Y	Y	Y	PY	N	NMA	NMA	Y	Y	NMA	Y	High
O'Logbon J.,<br>2024 [55]	Y	Y	Y	PY	Y	Y	PY	Y	Y	Y	Y	Y	Y	Y	Y	Y	High
Peckham E.,	Y	Y	Y	Y	Y	Y	PY	PY	Y	N	Y	Y	Y	Y	Y	Y	High

2017 [76]																	
Piñeiro B., 2016 [56]	N	N	Y	PY	Y	N	PY	Y	N	N	NMA	NMA	N	N	NMA	Y	Critically Low
Ricker A.B., 2024 [57]	Y	Y	Y	N	Y	N	Y	PY	Y	N	NMA	NMA	N	N	NMA	N	Critically Low
Saroj S.K., 2022 [58]	N	N	N	PY	N	Y	PY	Y	N	N	NMA	NMA	N	N	NMA	N	Critically Low
Sawyer C. 2023 [59]	Y	Y	Y	PY	Y	Y	PY	PY	N	N	NMA	NMA	N	Y	NMA	Y	Critically Low
Setchoduk K., 2023 [60]	Y	Y	Y	N	Y	Y	PY	PY	Y	N	Y	Y	Y	Y	Y	Y	Low
Shahab L., 2009 [61]	Y	N	Y	PY	N	N	N	PY	N	N	Y	Y	Y	Y	N	Y	Critically Low
Spanakis P., 2022 [62]	Y	Y	Y	PY	Y	Y	PY	Y	Y	N	Y	Y	Y	Y	Y	Y	High
Stead L.F., 2013 [78]	Y	Y	Y	Y	Y	Y	Y	Y	Y	Y	Y	Y	Y	Y	Y	Y	High
Stead L.F., 2017 [77]	Y	Y	Y	Y	Y	Y	Y	Y	Y	Y	Y	Y	Y	Y	Y	Y	High
Tatnell P., 2022 [63]	Y	Y	Y	Y	Y	Y	PY	Y	Y	N	NMA	NMA	Y	N	NMA	Y	Moderate
Taylor G.M.J, 2017 [64]	Y	Y	Y	Y	Y	Y	Y	Y	Y	Y	Y	Y	Y	Y	Y	Y	High
Thomsen T., 2014 [65]	Y	Y	Y	Y	Y	Y	Y	Y	Y	N	Y	Y	Y	Y	Y	Y	High
Tzelepis F., 2019 [79]	Y	Y	Y	Y	Y	Y	Y	Y	Y	Y	Y	Y	Y	Y	Y	Y	High
Villanti A.C., 2020 [66]	Y	N	Y	Y	Y	Y	Y	Y	Y	Y	NMA	NMA	Y	Y	NMA	Y	Low
Whittaker R., 2016 [68]	Y	Y	Y	Y	Y	Y	Y	Y	Y	N	Y	Y	Y	Y	Y	Y	High
Whittaker R., 2019 [67]	Y	Y	Y	Y	Y	Y	Y	Y	Y	Y	Y	Y	Y	Y	Y	Y	High
Williams P.J., 2023 [69]	Y	Y	Y	Y	Y	Y	PY	PY	Y	N	Y	Y	N	Y	N	Y	Critically Low
Zbikowski S.M., 2012 [70]	N	N	Y	N	Y	Y	PY	Y	N	N	NMA	NMA	N	N	NMA	Y	Critically Low
